# *Scorzonera* sensu lato (Asteraceae, Cichorieae) – taxonomic reassessment in the light of new molecular phylogenetic and carpological analyses

**DOI:** 10.3897/phytokeys.137.46544

**Published:** 2020-01-08

**Authors:** Maxim A. Zaika, Norbert Kilian, Katy Jones, Anastasiya A. Krinitsina, Maya V. Nilova, Anna S. Speranskaya, Alexander P. Sukhorukov

**Affiliations:** 1 Department of Higher Plants, Biological Faculty, Lomonosov Moscow State University, 119234, Moscow, Russia Lomonosov Moscow State University Moscow Russia; 2 Botanischer Garten und Botanisches Museum Berlin, Freie Universität Berlin, Königin-Luise-Str. 6–8, 14195 Berlin, Germany Freie Universität Berlin Berlin Germany; 3 I.M. Sechenov First Moscow State Medical University, Pharmaceutical Natural Science Department, Izmailovsky Boulevard, 8, 105043, Moscow, Russia I.M. Sechenov First Moscow State Medical University Moscow Russia

**Keywords:** phylogeny, carpology, Asteraceae, systematics, *
Scorzonera
*, Scorzonerinae

## Abstract

*Scorzonera* comprises 180–190 species and belongs to the subtribe Scorzonerinae. Its circumscription has long been the subject of debate and available molecular phylogenetic analyses affirmed the polyphyly of *Scorzonera* in its wide sense. We provide a re-evaluation of *Scorzonera* and other related genera, based on carpological (including anatomical) and extended molecular phylogenetic analyses. We present, for the first time, a comprehensive sampling, including *Scorzonera* in its widest sense and all other genera recognised in the Scorzonerinae. We conducted phylogenetic analyses using Maximum Parsimony, Maximum Likelihood and Bayesian analyses, based on sequences of the nuclear ribosomal ITS and of two plastid markers (partial *rbcL* and *matK*) and Maximum Parsimony for reconstructing the carpological character states at ancestral nodes. Achene characters, especially related to pericarp anatomy, such as general topography of the tissue types, disposition of the mechanical tissue and direction of its fibres, presence or absence of air cavities, provide, in certain cases, support for the phylogenetic lineages revealed. Confirming the polyphyly of *Scorzonera*, we propose a revised classification of the subtribe, accepting the genera *Scorzonera* (including four major clades: *Scorzonera* s. str., *S. purpurea*, *S. albicaulis* and *Podospermum*), *Gelasia*, *Lipschitzia***gen. nov.** (for the *Scorzonera divaricata* clade), *Pseudopodospermum*, *Pterachaenia* (also including *Scorzonera codringtonii*), *Ramaliella***gen. nov.** (for the *S. polyclada* clade) and *Takhtajaniantha*. A key to the revised genera and a characterisation of the genera and major clades are provided.

## Introduction

*Scorzonera* L. with some 180–190 species is the largest and name-giving genus of the subtribe Scorzonerinae of the Cichorieae (Kamelin and Tagaev 1986). It is widespread in the temperate and subtropical regions of Eurasia and N Africa, with the major centre of diversity in the arid and montane Irano-Turanian region. The genus is represented by perennial herbs often having a caudex or tuber, rarely by biennials or dwarf subshrubs, with linear to oblong, entire to pinnatisect leaves. The capitula have an involucre of imbricate phyllaries in several series. The achenes are cylindrical, with or without a tubular carpopodium and apically truncate or more rarely attenuate to beaked, ribbed or terete, hairy or glabrous, smooth or sculptured with stout conglomerations and their pappus is almost always of softly plumous bristles.

The Scorzonerinae are a morphologically clearly delimited subtribe, characterised by a unique plumose pappus (very rarely absent or non-plumose) of bristles with long soft and often intertwining fimbriae (Kilian et al. 2009a) and a pollen type of its own having colpori with only two (in all other members of the tribe with three) lacunae (Blackmore 1986) and forms a well-supported clade in phylogenetic analyses, based on morphological and molecular data (Bremer 1994; Whitton et al. 1995; Mavrodiev et al. 2004; Kilian et al. 2009a; Tremetsberger et al. 2013). The circumscription of *Scorzonera*, in contrast, has long been the subject of debate. Differences in morphological characters, such as leaf shape or achene shape and pubescence were, soon after the establishment of the genus, used to separate some taxa into the genera *Podospermum* (Candolle 1805), *Gelasia* (Cassini 1818) and *Lasiospora* (Cassini 1822), but only *Scorzonera* and *Podospermum* were accepted in the first comprehensive works on Asteraceae systematics by Lessing (1832) and Candolle (1838). Subsequent classification systems accepted *Scorzonera* again in a broader sense, with more or less elaborated infrageneric classification (Bentham in Bentham and Hooker 1873; Boissier 1875; Hoffmann 1890–1894; Lipschitz 1935, 1939, 1964). Three major entities were mostly accepted in the 20^th^ century: *Scorzonera* s.str., *Podospermum* and *Pseudopodospermum*, either at subgeneric rank (Lipschitz 1964; Chamberlain 1975; Chater 1976; Rechinger 1977) or, more rarely, at generic rank (Kuthatheladze 1978). First molecular phylogenetic analyses, using the nuclear ribosomal Internal Transcribed Spacer (nrITS: Mavrodiev et al. 2004; Kilian et al. 2009a) and nrITS + the External Transcribed Spacer (ETS) sequences (Winfield et al. 2006), revealed that *Scorzonera* is actually a polyphyletic assemblage. Its different clades were resolved intermixed with the clades formed by the traditionally accepted genera *Epilasia*, *Koelpinia*, *Pterachaenia*, *Tourneuxia* and *Tragopogon*, as well as by *Geropogon*. However, the number of *Scorzonera* species included in molecular analyses is so far fairly limited and the deeper nodes in these analyses frequently received only weak or no statistical support. Molecular phylogenetic analyses with a denser sampling of *Scorzonera* s.l. and also including plastid DNA markers is, therefore, inevitable for shedding more light on the phylogeny of this genus.

Whereas the traditionally recognised genera of the Scorzonerinae are morphologically well delimited, characterisation of the phylogenetic lineages of *Scorzonera* s.l., so far resolved, appears difficult. There is strong indication that the Scorzonerinae (as well as *Scorzonera* s.l.) are separated into lineages with different basic chromosome numbers (x = 6 and 7: Mavrodiev et al. 2004; Winfield et al. 2006; Altinordu et al. 2015) and that the *Scorzonera* clades differ in pollen morphology (Askerova 1970; Blackmore 1981, 1982, 1986; Díaz de la Guardia and Blanca 1985; Nazarova 1997; Mavrodiev et al. 2004; Türkmen et al. 2010; Pinar et al. 2016), but both features seem to be homoplastic. Achene and pappus morphology, but also achene anatomy, are significant in the systematics of the Asteraceae in general and the Cichorieae in particular (e.g. Becker 1913; Pak and Kawano 1990a, b; Kilian 1997; Sennikov and Illarionova 2001, 2002; Zarembo and Boyko 2008; Sukhorukov and Nilova 2015). A detailed recent survey of the achene morphology for 59 species of *Scorzonera* s.l. in Turkey, representing almost one third of all species, has been provided by Coşkunçelebı et al. (2016) but achene anatomy is known so far from five different members only (Boyko 2000; Makbul et al. 2012). Carpological studies, including anatomy and broad taxonomic sampling could, therefore, be informative for delimiting lineages within *Scorzonera* s.l.

The aims of the present study are (1) to provide a molecular phylogenetic analysis, using both nuclear and plastid DNA markers and a sampling that spans the entire subtribe, as well as the various groups of *Scorzonera* s.l.; (2) to investigate the variation in achene and pappus morphology and anatomy across the entire subtribe, to define carpological characters and states and to optimise them on to the new molecular phylogenetic tree, in order to assess the correspondence of carpological features with the principal clades and to gain insights into the evolution of carpological characters in the subtribe; (3) to characterise clades using carpological and, where available, further morphological and cytological characters, to review and, where sufficient evidence is provided, revise the current generic classification of the subtribe.

## Material and methods

### Sampling and material studied

In our carpological and molecular phylogenetic analyses, we included samples of the genus *Scorzonera* in its widest sense, as well as representatives of all other genera of the subtribe Scorzonerinae (Kilian et al. 2009b), to account for the previous findings that *Scorzonera* in all traditional circumscriptions is apparently polyphyletic. Leaf samples for DNA isolation and achene samples for carpological analysis were taken from live plants documented by herbarium specimens deposited in MW and from herbarium specimens of the herbaria B, BM, BR, E, FRU, G, HUJ, LE, MHA and MW with the permission of the curators. All samples used are listed in Table 1.

**Table 1.** Voucher material. Column three: presence of ** indicates that the specimen was sampled for both DNA sequence data and carpological characters. Column four gives the INSDC (International Nucleotide Sequence Database Collaboration) accession numbers for ITS, *matK* and *rbcL*, respectively; newly generated sequences are preceded by an asterisk (*), missing sequences are indicated with “NA”.

Taxon name	Sample designation	Voucher data (country, region, collecting date, collector and collecting number, herbarium code)	INSDC sequence accession numbers (ITS/matK/rbcL)
**Outgroup**			
*Avellara fistulosa* (Brot.) Blanca & C.Díaz	_1	Spain, Andalucía, Huelva prov., Almonte. 15 May 1981, *S.Talavera & B.Valdes s.n.* (MHA)	MN294710*/MN539152*/MN539153*
	_2	Portugal, Santa Luzia, 22 Jul 1961, *S. Paova & al*. *7936* (BR)	MN306173*/NA/NA
*Cichorium bottae* Deflers			AF118919.1/NA/NA
*C. intybus* L.			NA/JN895731.1/NA
			NA/NA/JQ230997.1
*Helianthus tuberosus* L.			NA/KT176589.1/NA
			NA/NA/KT178109.1
*Lactuca sativa* L.			NA/KT249996.1/NA
			NA/NA/KF613083.1
*L. serriola* L.			NA/KT250113.1/NA
			NA/NA/KM360838.1

*Nabalus tatarinowii* (Maxim.) Nakai			FJ980321.1/NA/NA under *Prenanthes macrophylla* Franch.
*Scolymus maculatus* L.			AJ633469.1/NA/NA
*Silybum marianum* (L.) Gaertn.			AY914831.1/NA/NA
** Scorzonerinae **			
*Epilasia acrolasia* (Bunge) C.B.Clarke ex Lipsch.	_1	Uzbekistan, Bukhara prov., 17 May 1982, *R. Lukashevich & V. Nikitin. s.n.* (MW0891688)	
	_3	Uzbekistan, Samarkand, 22 Jun 1958, *Yu. Gatkay s.n.* (FRU)	
	_2		AY508204.1/NA/NA
*E. hemilasia* (Bunge) Kuntze	_1	[Kyrgyzstan] Osh prov., 10 Jun 1978, *Aidarova s.n.* (FRU)	
	_2		AY508207.1/NA/NA
*E. mirabilis* Lipsch.		**Afghanistan, Doshi, 14 May 1964, *P. Furse 5920* (LE)	MN364398*/MN539154*/MN539155*
*Geropogon hybridus* (L.) Sch.Bip.		**Cyprus, Division 4, 18 Apr 2006, *A. Seregin & I. Privalova A-692* (MW0752412)	MN311752*/MN562544*/MN562550*
*Koelpinia linearis* Pall.	_1	[Turkmenistan] Western Kopet-Dag, alt. 1130 m, 23 May 1962, *I.A. Gubanov s.n.* (MW0891177) as *K. latifolia*	MN364399*/MN584908*/MN636825*
	_2		AY508203.1/NA/NA
	_3		AY508201.1/NA/NA
	_4	Uzbekistan, Western Tian-Shan, alt. 1340 m, 17 May 1963, *N. Pavlov s.n.* (MW0891202)	NA/MN584909*/MN636826*
*K. macrantha* C.Winkl.	_1	[Uzbekistan] Surkhandarya prov., 05 Jun 1979, *M. Pimenov et al. 447* (MW0891267)	NA/MN562548*/MN737477*
	_2		AY508200.1/NA/NA
*K. tenuissima* Pavlov & Lipsch.		[Uzbekistan] Karakalpakstan, 8 May 1983, *I.I. Rusanovich s.n.* (MW0891271)	
*K. turanica* Vassilcz.		** [Turkmenistan], South Kara Kum, 26 Apr 1963, *I.A. Gubanov 203* (MW0891280)	MN364404*/NA/NA
*Pterachaenia stewartii* (Hook. f.) R.R.Stewart	_1	**Afghanistan, Kandahar, 26 Apr 1967, *H. Freitag s.n.* (LE)	MN310887*/MN562545*/MN562551*
	_2		AY508211/NA/NA
	_3		AY508206.1/NA/NA
	_4	[Afghanistan] Paktia prov., alt. 1200 m, 13 May 1972, *O. Anders 8990* (W0032145)	
*Scorzonera acanthoclada* Franch.		**[Tajikistan] Magian-darya, 26 Jul 1964, *I. Schukin s.n.* (MW0891723)	MN306152*/MN584912*/MN636829*
*S. acantholimon* Hand.-Mazz.		Turkey, Turkish Kurdistan, alt. 1800-2450 m, 29 Jul 1910, *H. Handel-Mazzetti 539* (B100097180)	NA/NA/NA
*S. albicans* Coss.		[Spain, Albacete prov.] alt. 1600-2000 m, 28 Jun 1891, *J. Bornmüller 245* (B)	
*S. albicaulis* Bunge	_1	**Eastern Mongolia, western spurs of the Greater Hishan, 7 Jul 1991, *I.A. Gubanov & Sh. Dariymaa 1048* (MW0194319)	MN294988*/MN726384*/MN726410*
	_2	Russia, Amur region, Zavitinsky district, 25 Jul 1955, *I.A. Gubanov 2548* (MW0149686)	
*S. alpigena* (K.Koch) Grossh.	_1	**Syria, Hermon Mt., Jun 1974, *A. Shmida & Y. Lev-Ari s.n.* (HUJ) as S. cana var. alpina	MN322803* /MN726370*/MN726396*
	_2	**Cyprus, Division 5, alt. 300 m, 16 Mar 2005, *R. Hand 4343* (B100206142) as *S. subintegra*	MN322790* /MN726371*/MN726397*
*S. angustifolia* L.	_1		AJ633488.1/NA/NA
	_2	Spain, Granada prov., Galera, 22 Jun 1988, *D. Jeanmonod 1563/88* (B)	
*S. araneosa* Sm.		Greece, Santorini, alt. 500 m, 20 Apr 1985, *T. Raus s.n.* (B-100326685)	
*S. aristata* Ramond ex DC.	_3	[Italy, Trentino prov.] Bolzano, 28 Aug 1903, *J. Bornmüller s.n*. (B)	
	_1		AM117046.1/NA/NA
	_2		AY508192 1/NA/NA
*S. armeniaca* (Boiss. & A.Huet) Boiss.	_1	**Azerbaijan, Nakhichevan, 4 Jun 1968, *Sh. Kuthatheladze s.n.* (LE)	MN326304* /MN726379*/MN726405*
	_3	[Azerbaijan] Lerik region, 17 Jul 1963, *A.E. Bobrov & N.N. Tsvelev s.n.* (LE)	
	_4	[Armenia] Ararat district, 18 Jun 1977, *E. Nazarova s.n.* (B)	
	_2	**Azerbaijan, Nakhichevan, 31 May 1973, *N. Shvedchikova s.n.* (MW0635924) as *S. calcitrapifolia*	MN318329*/MN726380*/MN726406*
*S. aucheriana* DC.			HM802299.1/NA/NA
S. austriaca subsp. curvata (Popl.) Lipsch.		[Mongolia] Central region, 15 Jun 1944, *S.Yu. Lipshits s.n.* (LE)	
S. austriaca var. tenuifolia Lipsch. & Krasch.		[Austria] South Tyrol, 24 May 1905, *J. Bornmüller s.n.* (B)	
S. austriaca var. verrucosa Lipsch. & Krasch.		[Switzerland] near Lugano, alt. 850 m, 7 Jun 1895, *J. Bornmüller s.n.* (B)	
*S. austriaca* Willd.	_1	**[Russia] nr Krasnoyarsk, 23 Jun 1932, *N. Shmelev 32* (MW0149672)	MN296087*/NA /NA
	_2	[Russia] nr Krasnoyarsk, 25 Jun 1932 *N. Shmelev 6* (MW0149661)	
	_7	**Russia, Buryatia, Alkhanay National park, 10 Jul 2006, *Safronova & Golovin s.n.* (LE)	NA /MN726366*/MN726392*
	_3		GU724301.1/NA/NA
	_4		AM117047.1/NA/NA
	_5		KC968059 1/NA/NA
	_6		AY508216.1/NA/NA
*S. boetica* (DC.) Boiss.		Spain, Granada, Sierra de Cazorla, Jul 1972, *Fernandes s.n.* (B)	
*S. baldschuanica* Lipsch.		**[Kazakhstan] Shiki Valley, Jul 1963, *Shchukin s.n.* (MW0891755).	MN306194*/MN726386*/MN726412*
*S. biebersteinii* Lipsch.		[Azerbaijan] nr Kirovabad [Gyanja], 28 May 1968, *Sh. Kuthatheladze s.n.* (LE)	
*S. bracteosa* C.Winkl.		**Uzbekistan, Samarkand prov., 18 Jul 1913, *J. Bornmüller 51* (B)	MN306201*/NA/NA
*S. brevicaulis* Vahl	_1	**Algeria, Kabylie prov., Bouira, 22 Jun 1984, *A. Dubuis 12704* (B)	MN307902*/MN661240*/MN661228*
	_2	Algeria, Ghardaia, 26 Aug 1937, *R. Maire s.n*. (LE)	
*S. caespitosa* Pomel		**Morocco, above Imilchil on road to Lake Tizlite, alt. 2220 m, 7 Jun 1997, *A. Abaouz & al. 17578* (B100326682) as *S. pseudopygmaea*	NA/MN653932*/MN653931*
*S. callosa* Moris			AM117048.1/NA /NA
*S. calyculata* Boiss.	_1	**Iran, Hamadan, alt. 1900 m, Jun 1974, *K.H. Rechinger s.n*. (G267192)	MN307383*/MN661239*/MN661227*
	_2		AY508187.1/NA /NA
*S. cana* (C.A.Mey.) Griseb.	_1	**Iran, Khamseh, Jun 1975, *K.H. Rechinger 53825* (G266706)	MN317391*/MN726367*/MN726393*
	_5	Turkey, province Kars, 25 Jul 1981, *T. Raus 4596* (B) as S. cana var. jacquinina	
	_2	**Turkey, prov. Erzerum, Horasan, 13 Jun 1957, *P. Davis & I. Hedge 29379* (LE) as *S. floccosa*	MN319540*/MN726368*/MN726394*
	_6	Ukraine, Dnepropetrovsk region, 27 Jun 1982, *Kovalev s.n.* (MW0550690) as *S. jacquiniana*	
	_3		AY508193.1/NA/NA
	_4		AJ633480.1/NA/NA
	_7		AM117044.1/NA/NA
*S. capito* Maxim.	_1	[Mongolia] Alashan Gobi, Haldzan-Ula, 31 Jul 1989, *G.N. Ogureeva s.n.* (MW0194339)	
	_2	**[Mongolia] Toast-Ula Mts., 6 Aug 1975, *V.I. Grubov s.n.* (LE)	MN307485*/NA/NA
	_3	[Mongolia] East-Gobi prov., 7 Jul 1975, *Zhurba 622* (MW0194341)	
	_4	**[Mongolia], Bayan-Haucharsk prov., 27 Jul 1978, *G. Ogureeva s.n.* (MW0194339)	NA/MN695369*/MN695370*
*S. chantavica* Pavlov		[Kazakhstan] Karatau Mts., 30 May 1932, *P. Ignatova s.n.* (MW)	
*S. cinerea* Boiss.	_1	Turkey, Bulgardagh, Aug 1931, *A. Eig & M. Zohary s.n.* (HUJ)	
	_2		HM802289.1/NA/NA
*S. circumflexa* Krasch. & Lipsch.	_1	[Kyrgyzstan] Fergana Ridge, 20 Jun 1960, *Bylbaeva s.n.* (MW0891781)	
	_2		AY508214.1/NA/NA
*S. codringtonii* Rech.f.	_1	**Afghanistan, Bamian prov., alt. 3400 m, 25 Jul 1974, *I. Gubanov & al. 773* (MW0752232)	MN311479*/MN562547*/MN562553*
	_2	Afghanistan, Gardez prov., alt. 2850-3200 m, 5 Jun 1965, *K.H. Rechinger 31828* (B100326680)	
*S. crassicaulis* Rech.f.		Afghanistan, Bamian prov., 24 Jul 1962, *K.H. Rechinger 18747* (B)	
*S. cretica* Willd.	_1	Greece, Peloponnese, 13 Apr 1991, *W. Rosher s.n.* (B100417615)	
	_2		AJ633481.1/NA/NA
*S. crispa* M.Bieb.		[Ukraine] Nikita, 20 Jun 1960, *Belyanina s.n.* (B)	
*S. crispatula* (DC.) Boiss.	_1	Spain, Almeria, 16 Aug 1988, *P. Minissale & D. Valdes 847* (B)	
	_2		AJ633486.1/NA/NA
*S. crocifolia* Sm.		Greece, Arkadhia, 14 Jun 2010, *R. & E. Willing 201.321* (B100370148)	
*S. czerepanovii* Kamelin		Greece, Peloponnese, Lakonia, alt. 450 m, 10 Apr 1979, *W. Greuter 17084* (B) as *S. lanata*	
*S. davisii* Lipsch.		**Turkey, Hakkari to Başkale, 3 Aug 1954, *Davis & Polunin 2388* (LE)	MN307888*/MN661232*/MN661220*
*S. divaricata* Turcz.	**_1**	**[Mongolia], East Gobi, alt. 1150 m, 8 Jul 1982, *I.A. Gubanov 8585* (MW0194347)	MN312200*/NA/NA
	_2	**[Mongolia], East Gobi, 7 Jul 1971, *E.A. Isaichenko 37* (LE)	MN311251*/MN737473*/MN737475*
	_3	[Mongolia], East Gobi, 6 Jul 1981, *I.A. Gubanov 8581* (MW0194353)	
*S. doriae* Degen & Bald.	_1	**Montenegro, Niksic Grahovo, 22 Jul 2010, *R. Vogt s.n.* (B100346520)	NA/MN584913*/MN636830*
	_2		AJ633478.1/NA/NA as *S. doria*
*S. dzhawakhetica* Grossh.	_1	Georgia, Javakheti, 17 Oct 1967, *N. Grigorashvili s.n.* (LE)	
	_2	Georgia, Javakheti, 4 Aug 1948, *J. Sosnowski & S. Lipschitz 324* (LE) as *S. sosnovskyi*	
*S. elata* Boiss.		**Turkey, Antalya prov., 28 May 2011, *N. Kilian 10283* (B100426925)	MN307901*/MN661233*/MN661221*
*S. ensifolia* M.Bieb.		**Russia, Voronezh region, 18 Jul 1987, *V.N. Tikhomirov et al. s.n.* (MW0550595)	NA/MN636831*/MN636832*
*S. eriophora* DC.	_1	Turkey, Antalya prov., 19 Jun 2010, *R. Ulrich s.n.* (B100478256)	
	_2		HM802285.1/NA/NA
*S. filifolia* Boiss.		[Russia] Daghestan, alt. 3800 m, 2 Jul 1898, *T. Alexeenko s.n.* (LE)	
*S. franchetii* Lipsch.	_1	[Kazakhstan] Karatau, Mashat, 1 Aug 1934, *Pavlov 420* (MW0891823)	
	_2	[Kazakhstan] Kızıl-Orda, 13 Aug 1934, *N. Pavlov 373* (MW0891828)	
	_3	[Kazakhstan] Karatau, Mashat, 1 Sep 1934, *N. Pavlov s.n.* (MW0891822)	
*S. gracilis* Lipsch.		[Kyrgyzstan] Osh region, 1 May 1966, *Takaev s.n.* (FRU)	
*S. graminifolia* L.		Spain, Salas, Jun 1966, *P. Sotiaux s.n.* (BR813244)	
*S. grossheimii* Lipsch. & Vassilcz.	_1	**[Azerbaijan] Talysh, 29 Jun 1970, *Yu.Menitsky s.n.* (LE)	MN319579*/MN726378*/MN726404*
	_2	[Iran] Goran, alt. 1800 m, 8 Jun 1975, *K.H*. *Rechinger 53108* (B)	
*S. hieraciifolia* Hayek	_1	**Turkey, Niğde prov., alt. 1150 m, 27 Sep 1984, *I. Kageman & al*. *2151* (B)	MN317562*/NA/NA
	_2		HM802296.1/NA/NA
*S. hirsuta* L.	_1	Italy, Sicily prov., alt. 650 m, 30 May 1990, *F.M. Raimondo & al. 232* (B100485471)	
	_2		AM117051.1/NA/NA
	_3		AJ633479.1/NA/NA
	_4		AY508197.1/NA/NA under *Lasiospora hirsuta* (Gouan) Cass.
S. hirsuta var. villosaeformis Fiori		Italy, Matera prov., alt. 450 m, 7 Jun 1913, *A. Fiori 1994* (MW0795019)	
*S. hispanica* L.	_4	Russia, Krasnodar region, Novorossiysk district, 7 Jun 2008, *M.N. Kojin et al*. *Kr-341* (MW0636095)	
	_5	France, Montpellier, Fontfroide, 16 Jun 1944, *K.H. Rechinger 1590* (B100047704) as *S. glastifolia*	
	_1		AM117052.1/NA/NA
	_2		AY508186/NA/NA
	_3		AJ633472.1/NA/NA
	_6		AM117050.1/NA/NA under *S. glastifolia*
	_7	Czech Rep., Moravia, Karpati, Hodonin distr., Jun 1971, *S.Vicherek 1575* (MW)	NA/MN661231*/MN661219*
	_8	Russia, Voronezh region, 18 Jul 1988, *V.N*. *Tikhomirov* et al. *s.n.* (MW0550670)	
S. hispanica var. asphodeloides (Wallr.) Arcang.		Greece, Etolia-Akarnania prov., alt. 465 m, 20 May 2013, *R. & E. Willing 758* (B100564350)	
S. hispanica subsp. trachysperma (Fiori) Maire & Weiller			AM117055.1/NA/NA under *S. trachysperma* Günther ex Spreng.
*S. hissarica* C.Winkl.		**[Uzbekistan] Hissar Range, Boysun Mountains, 26 May 1935, *Gordienko s.n.* (MW0891836)	MN307486*/NA/NA
S. humilis f. alpina		[Austria] South Tyrol, alt. 1800 m, 23 Jul 1903, *J. Bornmüller s.n.* (B)	
*S. humilis* L.	_4	[Russian] Kursk prov., May 1919, *Alekhin s.n.* (LE)	
	_1		AM117053.1/NA/NA
	_2		AJ633476 1/NA/NA
	_3	**Russia, Lipetsk prov., Izmalkovsky distr., Jun 1994, *V.N. Tikhomirov* & al. *s.n.* (MW0550805)	MN295032* /MN726383*/MN726409*
*S. idaea* (Gand.) Lipsch.		**Greece, Crete, alt. 1550 m, 27 May 1983, *W. Greuter & H. Risse 19831* (B)	MN307892*/MN695363* MN695364*
*S. ikonnikovii* Krasch. & Lipsch.		**[Mongolia] Mongolian Altai, 27 Aug 1988, *I.A. Gubanov s.n.* (MW0194380)	MN307487*/MN695371*/MN695372*
*S. iliensis* Krasch.		[Kazakhstan] Almaty region, 20 Sep 1943, *N. Pavlov 45* (LE)	
*S. inaequiscapa* Boiss.		**Turkey, Mustafapaşa (Baraji), 19 May 2001, *F. Verloove 5329* (BR)	MN307489*/NA/NA
*S. incisa* DC.		**Azerbaijan, Nakhichevan, 26 Jul 1930, *A. Grossheim s.n.* (LE) as *S. bicolor*	MN310602*/MN695359*/MN695360*
*S. inconspicua* Lipsch.	_1	[Kyrgyzstan] Tian-Shan region, 12 Jun 1958, *Takaev s.n.* (FRU)	
	_2	[Kyrgyzstan] Kefgyzsky ridge, Kara-Archa, 7 Jun 1967, *Nikitin s.n.* (FRU)	
*S. intricata* Boiss.	_1	[Iran] W: Esfahan prov., alt. 1700-2000 m, 29 May 1974, *K.H. Rechinger 46707* (B)	
	_2		KF805090.1/NA/NA
*S. kandavanica* Rech.f.		Iran, Marandaraw, 21 Jun 1974, *K.H. Rechinger 48310* (B100097174)	
*S. ketzkhovelii* Grossh.		Georgia, Javakheti, alt. 1800 m, 30 Jul 1969, *L.Hiktibidze & Sh.Kuthatheladze s.n.* (LE)	
*S. kirpicznikovii* Lipsch.		**Georgia, Artvin district, 13 May 1907, *Yu.Voronov 1425* (LE)	MN322824*/MN726377*/MN726403*
*S. koelpinioides* Rech. f.		Pakistan, Quetta prov., alt. 1750-1900 m, 22 May 1965, *K.H. Rechinger 29987* (LE)	
*S. kotschyi* Boiss.	_2	Syria, Palestinian Territory, alt 650 m, 19 May 1933, *G. Samuelsson 5153* (B100355009)	
	_1		HM802297.1/NA/NA
*S. lachnostegia* (Woronow) Lipsch.		**Russia, Krasnodar region, Novorossiysk, 6 May 2015, *Popovich s.n.* (MW)	MN322804* /MN726369*/MN726395*
*S. laciniata* L.	_1	** Russia, Tambov region, 15 Jul 2008, *A.P. Sukhorukov s.n.* (MW)	MN317363*/MN726390*/MN726416*
	_6	[Ukraine] Kharkov region, May 1914, *Zalesskiy 391* (MW)	
	_7	[Russia] Dagestan, 1 Jun 1932, *N. Samsel s.n.* (MW)	
	_2	**USA, Colorado, Boulder, 9 Aug 1977, *Nekrasov s.n.* (MHA)	MN322791* /MN726391*/MN726417*
	_3		AY508194.1/NA/NA
	_4		AJ633473.1/NA/NA
	_5		AM117045.1/NA/NA
	_8		AJ633475.1/NA/NA
	_9	Hungary, Aug 1864, *Holuby s.n.* (MW) as *S. muricata*	
*S. lamellata* Krasch.		**Azerbaijan, Nakhichevan, 11 Jun 1947, *A.A. Grossheim et al*. *s.n.* (LE)	MN307886*/MN661237*/MN661225*
*Scorzonera lasiocarpa* D.F.Chamb.			JF781592.1/NA/NA
*S. latifolia* (Fisch. & C.A.Mey.) DC.	_1	**[Armenia] Gegamski Khrebet, Abovyan distr., alt. 1800 m, 20 Jul 1975, *V. Vasak s.n.* (B100356370)	MN339983*/NA/NA
	_2	**Iran, Azerbaijan prov., Ahar, Jul 1965, *A. Danin & U. Plitmann 65-1350* (HUJ)	NA/MN652618*/MN652617*
	_3		AY508190.1/NA/NA as *Lasiospora latifolia*.
*S. leptophylla* (DC.) Grossh.		Azerbaijan, Nakhichevan, 18 May 1933, *A.Grossheim s.n.* (LE)	
*S. libanotica* Boiss.		Lebanon, Bsherra, Aug 1931, *A. Eig & M. Zohary s.n.* (HUJ)	
*S. litwinowii* Krasch. & Lipsch.	_1	Iran, Shuturunkuh, 30 Apr 1963, *H. Freitag 14583* (B100356371)	MN339997*/NA/NA
	_2		AY508189.1/NA/NA
*S. longiana* Sümbül		**Turkey, Anatolia, Gazipapa, 2 Jul 1983, *H.Sumbul s.n.* (LE)	NA/MN584910*/MN636827*
*S. longipapposa* Rech. f.	_1	Afghanistan, Khost, 8 Jun 1967, *K.H. Rechinger 35772* (B)	
	_2	**Afghanistan, Khost, 3 Jun 1967, *K.H. Rechinger 35489* (G181688)	MN311477*/MN584911*/MN636828*
*S. luristanica* Rech.f.		**Iran, Kurdistan, Saqqez, alt. 2100 m, 20 Jun 1974, *K.H. Rechinger 48619* (G267216)	MN322805* /MN726375*/MN726401*
*S. mackmeliana* Boiss.		**Syria, Hermon, Ukkaf Sion, alt. 2150 m, Jun 1974, *A. Shmida s.n.* (HUJ)	MN364403*/NA/NA
*S. meshhedensis* (Rech.f.) Rech.f.		**Iran, Danjan, alt. 1700 m, 24 Jun 1960, *H. Pabot 3888* (G)	MN334193* /MN726373*/MN726399*
*S. meyeri* (K.Koch) Lipsch.		**Russia, Karachaevo-Cherkessia, alt. 2800 m, 4 Aug 2015, *M.A. Zaika s.n.* (MW)	MN334195* /MN726376*/MN726402*
*S. mirabilis* Lipsch.		Turkey, Kayseri prov., alt. 1300 m, 11 Aug 2002, *Karabulut s.n.* (B100173606)	
*S. mollis* M.Bieb.	_1	**Feodosia, 15 Jun 1993, *I. Volkovskaya s.n*. (MW0630360)	MN305796*/MN661238*/MN661226*
	_2	**Syria, Hermon Peak, alt. 2000 m, May 1984, *A. Liston 7-84-221/2* (HUJ)	MN305997*/NA/NA
	_3	East Crimea, 15 May 1993, *I. Volkovskaya* & N. *Belyanina s.n.* (MW0630360)	
S. mollis var. platyphylla Boiss.		**Iran, Shuturunkuh, Apr 1963, *Bot. Exp*. *721* (HUJ)	MN307890*/NA/NA
*S. mongolica* Maxim.	_3	[Mongolia] Dzhungaria, 3 Aug 1988, *S. Dariymaa et al. 2818* (MW0194397)	
	_4	Mongolia, SW East Gobi prov., alt. 1000 m, 5 Aug 1989, *I.A. Gubanov & V.I. Grubov 361* (MW0194396)	
	_1		KF454421.1/NA/NA
	_2		KF454420.1/NA/NA
*S. mucida* Rech.f., Aellen & Esfand.		Iran, Kerman prov., 24 May 1962, *J. Sojak 539* (B100475187)	MN307889*/NA/NA
*S. musilii* Velen.		Yemen, Rada town, alt. 2000 m, 12 Apr 1977, *N. Kilian & al. 4900* (B100220796)	
*S. ovata* Trautv.		[Turkmenistan] Ashkhabad region, 7 May 1985, *Rusanovich s.n.* (MW0891889)	
*S. pachycephala* Podlech & Rech.f.		**Afghanistan, Kandahar prov., 22 Apr 1972, *O. Anders 8780* (LE)	MN733278*/MN661236*/MN661224*
*S. papposa* DC.	_1	**Israel, Upper Galilee, Apr 1962, *U. Plitmann s.n.* (HUJ) as *S. kurdica*	MN306528*/NA/NA
	_2	**Nakhichevan, 27 Jun 1972, *O. Lovelius s.n.* (LE)	NA/MN661241*/MN661229*
	_3		AY508210.1/NA/NA
	_4	[Iran] Kerman prov., alt. 2000 m, 28 May 1892, *J. Bornmüller 4131* (B) as *S. picridioides*	
*S. parviflora* Jacq.	_1	Russia, Belgorod region, 12 Jun 2008, *A. Gusev s.n.* (MW0550933)	
	_2	[Kyrgyzstan] Issyk-Kul Basin, 5 Jul 1963, *Inchin s.n.* (FRU)	
	_3		KF454416.1/NA/NA
*S. persepolitana* Boiss.		[Iran] Kashan prov., 3 May 1974, *K.H. Rechinger 46913* (B100355005)	
*S. petrovii* Lipsch.		[Kazakhstan] Syr-Darya prov., 2 Jul 1934, *N. Pavlov 22* (MW0891932)	
*S. phaeopappa* (Boiss.) Boiss.	_1	**Iran, alt. 1700 m, 10 May 1937, *J. Bornmüller 1094* (B)	MN307887*/NA/NA
	_2	[Syria] Hermon, Namneman ridge, alt. 1600 m, May 1984, *A. Liston 7-84-219/8* (HUJ) as *S. multiscapa* Boiss.	
*S. pisidica* Hub.-Mor.		**Turkey, Burdur Dirmil, 17 Jun 1981, alt. 1270 m, *A. Huber-Morath 16274* (LE)	NA/MN652619*/MN653930*
*S. polyclada* Rech.f. & Köie	_1	**Afghanistan, Ghazni prov., 1 Jul 1974, *I. Gubanov & al*. *362* (MW0752240)	MN311478*/MN562546*/MN562552*
	_2	Afghanistan, Kabul prov., alt. 2000 m, 21 Jun 1965, *K.H. Rechinger 31240* (B)	
*S. pseudodivaricata* Lipsch.	_1	**[Mongolia] Gobi-Altai prov., alt. 1500 m, 10 Jul 1984, *I.A. Gubanov 8816* (MW0194378)	MN296016*/MN695365*/MN695366*
	_3	[Mongolia] Mid-Gobi prov., 4 Jul 1979, *N.P. Guricheva & V.I. Grubov s.n.* (LE)	
	-4	[China] Dzhungaria, Urumqi region, 5 Sep 1929, *M. Popov & S. Lipschitz s.n.* (LE)	
	_2		KF454418.1/NA/NA
*S. psychrophila* Boiss. & Hausskn.	_3	Israel, Negev desert, Mar 1945, *N. Tadmor s.n.* (HUJ) as *S. judaica*	
	_1	Azerbaijan, Nakhichevan, 30 May 1933, *A.Grossheim s.n.* (LE) as *S. pseudolanata*	MN339969*/NA/NA
	_2		HM802294.1/NA/NA
*S. pubescens* DC.		**[Kyrgyzstan] Suzamyr, 22 Jun 1973, *Aidarova s.n.* (FRU)	MN307900*/NA/NA
*S. purpurea* L.	_1	** Kazakhstan, Kostanai region, 22 Jun 1940, *Schroeter s.n.* (MW0896165)	MN313187*/NA/NA
	_2	**Russia, Tambov prov., Kirsanov distr., Jun 1996, *A. Sukhorukov s.n.* (MW0551082)	MN313257*/MN726381*/MN726407*
	_3		AJ633477.1/NA/NA
S. purpurea subsp. peristerica Formanek.		Greece, [Phthiotis] Ghiona Mt., alt. 1900-2000 m, 27 Jul 1906, *R. Maire & M. Petitmengin* (B)	
*S. pusilla* Pall.	_1	[Uzbekistan] Bukhara region, 15 Apr 1986, *M. Pimenov et al. 21* (MW0892018)	
	_2	Israel, Negev Highlands, near Har Hemet, 14 Apr 1992, *A.Danin s.n.* (B)	
	_3	**[Kyrgyzstan] Osh region, 10 May 1978, *Aidarova s.n.* (FRU)	MN295460*/MN695373*/MN695374*
	_4		AY508205.1/NA/NA under *Takhtajaniantha pusilla* (Pall.) Nazarova
	_5	[Iran] Kasandschik, 28 Apr 1901, *J. Bornmüller 1614* (B100001063)	
*S. pygmaea* Sm.	_1	[Morocco] alt. 1800 m, 7 Jun 1927, *F. Quer 726* (B)	
	_2		KF925533.1/NA/NA
*S. racemosa* Franch.		** [Kazakhstan] Heptapotamia, Taldy-Kurgan region, 13 Sep 1928, *Bykov 365* (MW0892043)	MN306153*/NA/NA
*S. raddeana* C.Winkl.	_1	[Iran] Khoragan prov., 13 Jun 1975, *K.H*. *Rechinger 53301* (B)	
	_2		AY508212.1/NA/NA
*S. radiata* Fisch. ex Ledeb.		[Russia] Tyva Rep., alt. 2400 m, 3 Sep 1946, *A. Schroeter s.n.* (MW0149627)	
*S. radicosa* Boiss.		**Turkey, C4 Karaman, alt. 2320 m, 22 Jul 1999, *D. Tolimir s.n.* (B)	MN322806* /MN726374*/MN726400*
*S. ramosissima* DC.		Iraq, Erbil, alt. 2000 m, 10-14 Aug 1957, *K.H. Rechinger 11351* (B)	
*S. rawii* Rech.f. & Guest		Iraq, alt. 700 m, 7–8 Jun 1957, *K. H. Rechinger 9950* (B)	
*S. renzii* Rech.f.		Iran, W Azerbaijan, alt. 1800-2400 m, 1 Jul 1974 *S. Renz s.n.* (LE)	MN307893*/MN726388*/MN726414*
*S. reverchonii* Debeaux & Hervier		Spain, Sierra de Gazorla, alt. 1700 m, May 1901, *E. Reverchon 1229* (B)	
*S. rhodantha* Hausskn.		Greece, Epirus prov., alt. 1600 m, 2 Aug 1956, *K.H. Rechinger 18566* (B)	
*S. rigida* Aucher ex DC.	_1	[Armenia] nr Yehegnadzor, 21 Jul 1950, *J. Mulkidzhanyan s.n*. (LE)	
	_2		AY508191.1/NA/NA
*S. rosea* Waldst. & Kit.	_3	Slovenia, Creina, Jul 1894, *Korta s.n.* (B)	
	_1	**[Austria] Carpathian mountains, alt. 900 m, 6 Aug 1928, *Schwarz s.n.* (B)	MN313575*/MN726382*/MN726408*
	_2		IKM262852.1/NA/NA as *Podospermum roseum*
*S. rupicola* Hausskn.		**Iran, 20 Apr 1964, *T.H. Strauss 286* (B)	MN306200*/NA/NA
*S. sandrasica* Hartvig & Strid			JF781591.1/NA/NA
*S. schischkinii* Lipsch. & Vassilcz.		Russia, Krasnodar region, Novorossiysk, 6 May 2015, *Popovich s.n.* (MW)	
*S. seidlitzii* Boiss.	_1	**Georgia, Ajaria, Zekadsky pass, 24 Jul 1969, *Yu. Menitsky s.n.* (LE)	NA/MN661230*/MN661218*
	_2	[Armenia] Spitak district, 15 Jun 1977, *E. Gabrielyan s.n.* (B)	
	_3		AY508188.1/NA/NA
*S. sericea* DC.		Turkey, C5 Adana prov., alt. 2700-3000 m, 6 Aug 1999, *M. Doring & al*. *7074* (B10020487)	
*S. songorica* (Kar. & Kir.) Lipsch. & Vassilcz.	_1	**[Kyrgyzstan] Osh Region, 20 Jun 1968, *Utuleeva s.n.* (FRU)	MN322823*/MN726372*/MN726398*
	_2	[Kazakhstan] Karatau Mts., 13 Jun 1931, *N. Pavlov 190* (MW0892063)	
	_3	Afghanistan, 26 May 1950, *Dainet 335* (B) as S. cana var. praticola	
*S. stricta* Hornem.		[Russia] Chechnya, Sunzha Ridge, 28 May 1988, *V. Prima 485* (LE)	
*S. subacaulis* (Regel) Lipsch.		Kyrgyzstan, Issyk-Kul region, Sary Jaz river, 14 Jun 1984, *Aidarova s.n.* (FRU)	MN307484*/NA/NA
*S. suberosa* K.Koch	_1	**[Armenia] nr. Yerevan, 29 May 1896, *A.Shorzh s.n.* (LE)	MN305801*/MN661235*/MN661223*
	_2		AY508199.1/NA/NA
*S. syriaca* Boiss. & Blanche		**Israel, Upper Galilee, Apr 1976, *A. Shmida & al. s.n.* (HUJ)	MN307891*/MN661234*/MN661222*
*S. szowitzii* DC.		[Armenia] nr Sisuana, 4 Jun 1972, *S. Lipschitz 633* (LE)	
*S. tau-saghyz* Lipsch. & G.G.Bosse	_1	**[Kazakhstan], Zhambyl region, 19 Jun 1948, *N. Pavlov s.n.* (MW0892092)	MN307488*/MN695367*/MN695368*
	_2	[Kazakhstan] NW Karatau, 2 Jul 1935, *N. Pavlov s.n.* (MW) as *S. mariae*	
	_3	Kazakhstan, Kyzyl-Orda region, Karatau Mts., 19 May 1985, *M. Pimenov & S. Mehibaev 70* (MW0892095) as *S. vavilovii*	
*S. tomentosa* L.		Turkey, Sivas prov., 4 Aug 1997, *P. Hein 4414* (B100326699)	
*S. tortuosissima* Boiss.		[Iran] Kerman prov., alt. 1800 m, 6 May 1977, *K.H. Rechinger 55224* (B)	
*S. tragopogonoides* Regel & Schmalh.	_1	[Kazakhstan] Mount Kara-Ray, 19 May 1932, *Kryltsov s.n*. (MW0891887)	
	_2	[Kyrgyzstan] Chatkal valley, Sandalash, 6 Jun 1977, *Gorbulova s.n.* (FRU)	
	_3	[Kyrgyzstan] Chatkal river, 15 Jun 2001, *G. Lazkov s.n.* (FRU)	
	_4	**[Uzbekistan, Samarkand prov.] Urgut, 31 May 1978, *Pimenov & al. s.n.* (MW0892117)	MN306193*/MN726389*/MN726415*
*S. transiliensis* Popov	_1	[Kazakhstan] Prov. Heptapotamia, distr. Kegen, 9 Jul 1932, *S. Lipschitz 271* (MW0892149)	
	_2	[Kyrgyzstan] Issyk-Kul lake, 28 Jul 1968, *Aidarova s.n.* (FRU)	
*S. troodea* Boiss.		Cyprus, Agios Filippos, alt. 800 m, 10 Jun 2011, *R. Hand 5810* (B)	
*S. turkestanica* Franch.		**[Kyrgyzstan] Osh region, 25 Sep 1983, *Aidarova s.n.* (FRU)	MN306202*/MN726385*/MN726411*
*S. semicana* DC.		[Russia] Krasnodar region, Novorossiysk distr., 30 May 1966, *E. Gogina & G. Proskuryakova 57* (MHA) as *S. turkeviczii*	
*S. tuberosa* Pall.		[Kazakhstan] Kostanay region, 2 Jun 1946, *S.D. Erpert s.n.* (MW0892134)	
*S. turcomanica* Krasch. & Lipsch.		[Turkmenistan] E Kopet-Dag, 50 km SE of Ashgabat, 8 May 1963, *I.A. Gubanov s.n.* (MW0892046)	
*S. ulrichii* Parolly & N.Kilian		Turkey, Antalya, 18 Jun 2002, *R. Ulrich s.n.* (B100086256)	
*S. undulata* Vahl	_1	**Algeria, Chellala, 6 May 1965, *V.P. Bochantsev 1912* (LE) as *S. alexandrina*	MN306573*/MN695361*/MN695362*
	_2		AJ633485.1/NA/NA
S. undulata subsp. deliciosa (Guss. ex DC.) Maire			AM117049.1/NA/NA under *S. deliciosa*
*S. veresczaginii* Kamelin & S May Smirn.		Kazakhstan, Altai Mountains, Azutan range, 13 Jun 1984, *Yu.A. Kotukhov s.n.* (B100001002)	
*S. villosa* Scop.	_1	**Italia, Puglia prov., 16 May 1961, *K.H. Rechinger 23425* (B)	NA/MN636833*/MN652616*
	_2		AJ633482.1/NA/NA
	_3		AM117056.1/NA/NA
*S. violacea* D.F.Chamb.		Turkey, C4 Konya prov., alt. 1700 m, 10 Jul 2000, *O. Eren & G. Parolly 7887* (B100204872)	
*S. virgata* DC.		India, Himachal Bradesh, Lahul, Sep 2000, *Pimenov & Klukov 141* (MW0752430) under *S. divaricata*	MN306199*/MN726387*/MN726413*
*Tourneuxia variifolia* Coss.	_3	Algeria, Ghardaia, 30 Mar 1965, *V.P. Bochantsev 862* (LE)	
	_1	**Algeria, Oasis Diamae, 30 Apr 1963, *L. Faurel 5041* (BR)	MN294711*/MN737474*/MN737476*
	_2		AY508217.1/NA/NA
	_4		AY508202.1/NA/NA
*Tragopogon brevirostris* DC.			AY508174.1/NA/NA
*T. crocifolius* L.			AY508180.1/NA/NA
*T. dubius* Scop.			NA/KP210437.1/NA
			NA/NA/JX848433.1
*T. kotschyi* Boiss.			AY508181.1/NA/NA
*T. longirostris* Sch.Bip.			AY508185.1/NA/NA
*T. porrifolius* L.			AY508169.1/NA/NA
*T. pratensis* L.			NA/JN895071.1/NA
			NA/NA/KJ841627.1

### DNA extraction, amplification and sequencing

Total DNA was extracted from 30 mg of dried plant material. Samples were manually homogenised in a paper envelope and a modification of the CTAB-method of Doyle and Doyle (1987) by Krinitsyna et al. (2015) was used. After lysis with CTAB-lysis buffer (2% CTAB, 0.1 M Tris-HCl (pH 8.0), 1.4 M NaCl, 20 mM EDTA (pH 8.0), 1% PVP, 0.2% 2-mercaptoethanol, 0.1 mM DTT) for 1 hour at 60 °C, the mixture was centrifuged for 5 min at 13,400 rpm (12.080 g) and the supernatant was transferred to a new tube. An equal volume of propyl alcohol was added to the lysate and the samples were incubated at room temperature for 10 min and centrifuged again for 10 min as above. The supernatant was discarded and the precipitate was washed twice using 80% ethanol, dried for 3–5 min and dissolved in 30 µl of deionised water. The stock solution of magnetic particles (Agencourt AMPure XP, Beckman Coulter) was diluted and thoroughly mixed with the buffer (18% PEG-8000, 1M NaCl, 10mM Tris-HCl ph 8.0, 1mM EDTA pH 8.0) in the ratio of 1 to 3 (v/v). Then DNA samples were purified with the suspension of magnetic beads at a ratio of 1:1 (v/v). Concentration and purity of DNA samples were assessed by OD 260/280 and OD 260/230 ratios on the NanoPhotometer N60-Touch (Implen, Germany). DNA samples were normalised to 10 ng/µl before sample preparation. Then 5 µl (50 ng) of each normalised sample was used for PCR and library preparation.

Sequences of three markers were used for the molecular phylogenetic analyses: (1) the nrITS region (including ITS1, 5.8S rRNA gene and ITS2); (2) a ~570 bp fragment of the plastid DNA ribulose 1,5-bisphosphate carboxylase/oxygenase large subunit (*rbcL*) gene; (3) a 750 bp fragment of the plastid DNA maturase K gene (*matK*).

The nrITS region was sequenced on the MiSeq (Illumina, USA). A two-step PCR method was used for library preparation: first-stage PCR using fusion primers containing the primer sequences from Baldwin (1992) and White et al. (1990) and Illumina adaptor tails: ITS5-Illu-F and ITS2-Illu-R; ITS3-Illu-F and ITS4-Illu-R. PCRs were carried out in Thermal Cycler T100 (Bio-Rad, USA) under the programme: 95° – 10 min; 30 cycles each for 95° – 5 s, 50° – 20 s, 72° – 20 s; finally 72° – 5 min. PCR products (expected size of 400 bp) were checked on 1.2% agarose gels and purified with the suspension of magnetic beads Agencourt AMPure XP (Beckman Coulter, USA) in the ratio of 1:1.4 (v/v) and mixed in equimolar proportions for each mix. Second-stage PCR performed with 50 ng of mixed products and Nextera index primers (Illumina, USA), according to manufacturer’s instructions. Both PCR stages were conducted using Taq DNA polymerase (New England Biolabs, USA). Illumina libraries were sequenced on the MiSeq with MiSeq Reagent Kit v2 for 500 cycles (Illumina, USA).

The *rbcL* marker was sequenced on the 454 platform (GS Junior, Roche, Switzerland). The three-step PCR method was used for library preparation. All PCR stages were conducted using Phusion high fidelity DNA-polymerase (New England Biolabs, USA). The first-stage PCR was performed using a reaction mixture of a total volume of 20 µl: 4 μl 5× buffer for Phusion high fidelity DNA-polymerase, 250 µM dNTP (Thermo Scientific, USA), 0.2 μl Phusion high fidelity DNA-polymerase and 0.8 pM each primers (rbcLa-F and rbcLa-R, Hollingsworth et al. 2009).

The PCR conditions were as follows: 98 °C – 3 min; 7 cycles of 98 °C – 5 s, 50 °C – 30 s, and 72 °C – 30 s; finally 72 °C – 5 min. Amplification products were used without purification for the second round of PCR which was performed using primers rbcLa-454-F and rbcLa-454-R. PCR was performed using a reaction mixture of a total volume of 20 µl: 4 μl 5× buffer of Phusion high fidelity DNA-polymerase, 250 μM dNTP (Thermo Scientific, USA), 0.2 μl of Phusion high fidelity DNA-polymerase, 0.3 pM of each primer, 4 μl of amplification products of the previous stage. PCR conditions: 98 °C – 1 min; 18 cycles each for 98 °C – 5 s, 55 °C – 30 s and 72 °C – 30 s; finally 72 °C – 5 min. PCR products (expected size of 600 bp) were checked on 1.2% agarose gels and without purification used for third-stage PCR with primers MIDh-454-F and MIDh-454-R, containing barcodes (MIDx) and adapter sequences for sequencing on the 454 platform (GS Junior Systems, Roche, Switzerland). Adapter sequences are described in Gholami et al. (2012). Sample preparation was carried out with a protocol for the sequencing of amplicons Lib-A. Sequencing of the 454 platform (GS Junior, Roche, Switzerland) was performed according to the manufacturer’s instructions.

The *matK* marker was amplified using the primers matK_f (KIM3F) and matK_r (KIM1R) (Hollingsworth et al. 2009). A total of 50 ng DNA was used for a total volume of 25 μl reaction mix, containing 1 μM of each primer, 200 μM of each dNTP and 0.5 U Taq DNA polymerase (“New England Biolabs”, USA). PCRs were carried out in Thermal Cycler T100 (Bio-Rad, USA) under the programme 95 °C – 10 min; 30 cycles each for 95 °C – 5 s, 57 °C – 30 s, 72 °C – 30 s; finally 72 °C – 5 min. PCR products (expected size of 900 bp) were checked on 1.2% agarose gels and purified with the suspension of magnetic beads Agencourt AMPure XP (“Beckman Coulter”, USA) in the ratio of 1:1 (v/v). Sanger sequencing was performed on an Applied Biosystems 3730 DNA Analyzer using ABI PRISM BigDye Terminator v. 3.1 reagents. The reads were assembled using CLC Genomics Workbench ver. 10.0.1 (CLC Bio, Aarhus, Denmark; https://www.qiagenbioinformatics.com).

In addition to the 72 nrITS, 65 *matK* and 65 *rbcL* sequences generated in the present study, some published sequences (see Table 1) were taken from the INSDC (International Nucleotide Sequence Database Collaboration).

### Sequence alignment and phylogenetic analyses

Sequences were aligned with MAFFT version 7 using default parameters (Katoh et al. 2017) and the alignment was adjusted manually using PhyDe (version 0.9971; Müller et al. 2010). Simple indel coding, according to Simmons and Ochoterena (2000) implemented in SeqState v.1.40 (Müller 2005), was applied to the ITS matrix which showed length mutational variation in contrast to the plastid DNA matrix. Phylogenetic relationships were reconstructed separately for the nuclear and plastid DNA datasets using maximum parsimony (MP), Bayesian inference (BI) and maximum likelihood (ML). The MP analyses were performed applying the parsimony ratchet (Nixon 1999) with PRAP v.2.0 (Müller 2004) in combination with PAUP v.4.0b10 (Swofford 2003), using 200 ratchet iterations with 25% of the positions randomly upweighted (weight = 2) during each replicate and 10 random addition cycles. Jackknife (JK) support values were calculated in PAUP with 10,000 jackknife replicates and the TBR branch swapping algorithm with 36.788% of characters deleted and one tree held during each replicate. The ML analyses were done with RaxML (Stamatakis 2014) in the version of RAxMLHPC v.8 on XSEDE on the CIPRES Science Gateway (Miller et al. 2010). Four partitions were separated in the nrITS dataset (three DNA: ITS1, 5.8s, ITS2, one binary), whereas the plastid DNA dataset with exclusively coding sequences was left unpartitioned. A rapid bootstrap analysis and search for the best-scoring ML tree in one single programme run with 100 bootstrap replicates was carried out using the general time-reversible (GTR) + Γ model as the standard predefined substitution model in RAxML. The BI analyses were performed with the MPI version of MrBayes (Ronquist et al. 2012) on the high-performance computing system of the Freie Universität Berlin. Instead of *a priori* testing of the optimal substitution model, sampling across the entire general time reversible (GTR) model space in the Bayesian MCMC analysis was applied (Huelsenbeck et al. 2004). Four partitions were separated in the nrITS dataset (as in ML) and two partitions in the plastid DNA dataset (*matK*, *rbcL*). Two simultaneous runs of four parallel chains each were performed for 3 × 10^7^ generations with a sample frequency of 1 tree per 2000 generations. Convergence of the runs was checked by making sure that the average standard deviation of split frequencies of the post-burn-in runs was below 0.01 and the effective sampling size (ESS) well above 200 in either run for all parameters. TreeGraph v.2 (Stöver and Müller 2010) was used to visualise the trees with statistical node support.

### Carpological analysis

In total, 149 species of Scorzonerinae including *Scorzonera* s.l. (141 sp.), *Koelpinia* (3 sp.), *Pterachaenia* (1 sp.), *Epilasia* (3 sp.) and *Tourneuxia* (1 sp.) were carpologically studied. Carpological data for the genera *Tragopogon* and *Geropogon* were taken from a previous study by Sukhorukov and Nilova (2015).

Only marginal achenes of a capitulum were selected for the analysis. The cross-sections were made by hand in three topographical zones: in the basal third of the achene body, in the median third and in the apical third (or beak if present). The medium portion of the outer achenes is the most informative part, where all features are fully developed and was used for the carpological descriptions and phylogenetic reconstruction. Longitudinal sections were prepared in some cases to detect the peculiarities in the parenchymatous structures of the mesocarp. Prior to sectioning, the achenes were soaked in a mixture of ethyl alcohol, water and glycerol in equal proportions for a few days at 30–40^o^C. The sections were stained with 2% carbol fuchsin, then with 0.4% picroindigocarmine and 100% ethanol (preparation of the reagents after Barykina et al. 2004).

The morphology of the achenes was documented using an Olympus SZ61 camera; the cross-sections were studied using Nikon Eclipse Ci microscopes and documented with a Nikon DS-Vi1 camera (Nikon Corporation, Japan) at the Department of Higher Plants (Moscow State University). The ultrasculpture of the achene surface was investigated using an SEM (JSM-6380 LA) at the Laboratory of Electron Microscopy Center at Moscow M.V. Lomonosov State University.

### Characterisation of diaspore morphology and anatomy, character coding and analysis of character distribution and evolution

Describing carpological features, we applied the tripartite descriptive model (see, for example, Henning et al. 2018), where a feature comprises a character, which is, for its definition, decomposed into a “structure” (a physical component of an organism, for example, a carpophore) and a “property” (e.g. presence) and they were coded with binary or multiple unordered categorical states. Based on our carpological analysis, we selected and defined 16 achene characters potentially relevant for the reconstruction of character evolution. The character data matrix was built and analysed with Mesquite version 3.5 (Maddison and Maddison 2014; Mesquite Project Team 2014). Reconstruction of the character states at ancestral nodes was analysed with MP using the nrDNA tree as hypothesis of phylogenetic relationships and the ‘Trace character history’ and ‘Trace all characters’ tools as display methods. The character definitions and their state coding are provided under Results.

## Results

### Molecular phylogenetic analyses

Phylogenetic reconstruction based on the nrITS region

**Figure 1.**
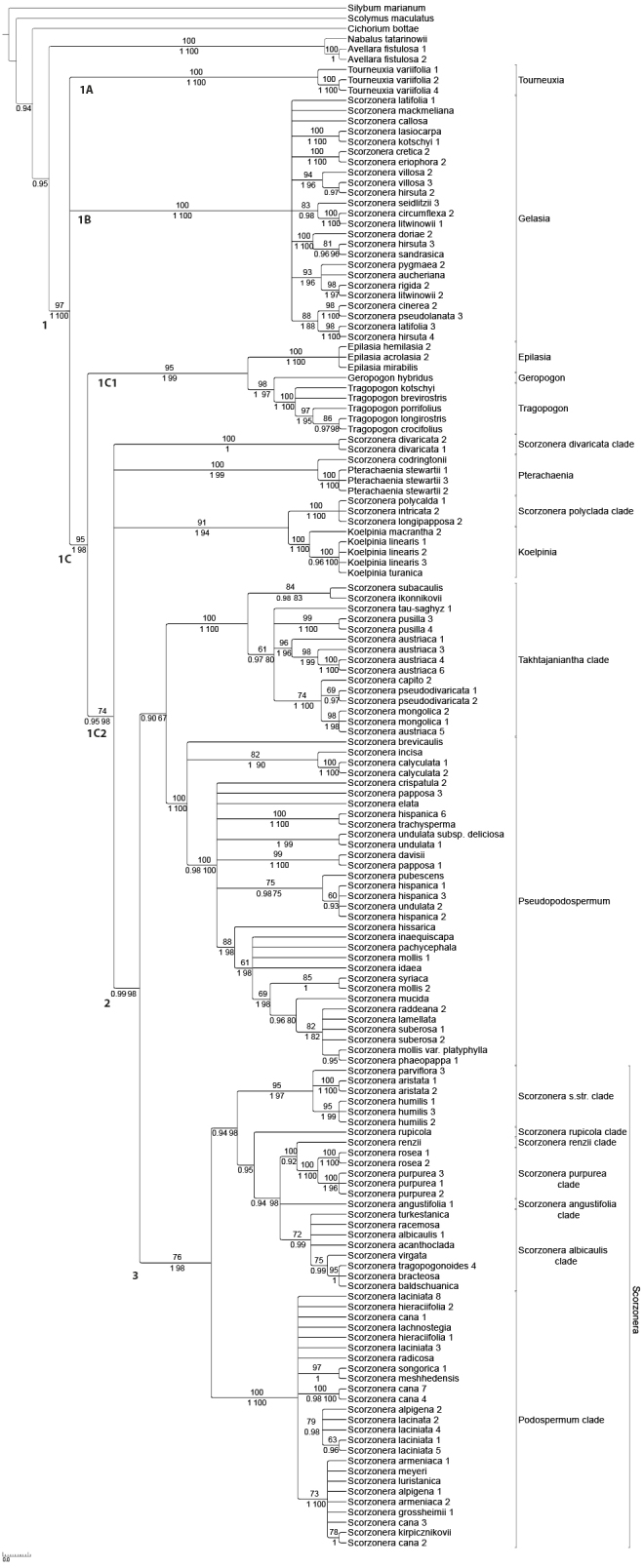
Majority consensus cladogram of the *Scorzonera* s.l. from the Bayesian analysis, based on the ITS dataset (support values: Maximum Parsimony jackknife, second line: Bayesian posterior probability and Maximum Likelihood bootstrap).

The alignment of the nrITS region had a length of 707 characters; together with the coded indels, the matrix included a total of 832 characters, of which 422 were parsimony-informative. The MP analysis resulted in 697 most parsimonious trees (L = 2202, CI = 0.416, RI = 0.848, RC = 0.353, HI = 0.584). The 50% majority consensus tree was largely congruent in topology with the 50% majority consensus trees of the BI and ML analyses. Fig. 1 shows the BI cladogram with the MP jackknife (JK) support values above and the BI posterior probabilities (PP) and ML bootstrap (BS) support values below the branches.

The Scorzonerinae are resolved as monophyletic with strong statistical support (clade 1; JK = 97, PP= 1, BS = 100). The subtribe includes three major clades (1A, 1B, 1C), which received strong (1C; JK = 95, PP = 1, BS = 98) or full statistical support (1A, 1B). Their relationships to each other are unresolved. Clade 1A solely comprises the genus *Tourneuxia*. Clade 1B includes one part of *Scorzonera* s.l. We designate it as *Gelasia* clade, because it includes *Scorzonera villosa*, which provides the type of *Gelasia* Cass., being the oldest available generic name for members of this clade. Clade 1C includes the large remainder of the subtribe separated in two well-supported subclades. One (clade 1C1, JK = 95, PP = 1, BS = 99) comprises the genera *Tragopogon*, *Geropogon* and *Epilasia*; the other (clade 1C2, JK = 74, PP = 0.96, BS = 98) is a polytomy of four clades: (1) *Scorzonera divaricata* forming a clade of its own; (2) *Pterachaenia* with *S. codringtonii* as sister; (3) *Koelpinia* and a *S. polyclada* clade; (4) a large clade including both *Scorzonera* in the sense of its type *S. humilis* and *Podospermum* in one well supported subclade (JK = 76, PP = 1, BS = 98) and in a second moderately supported subclade (PP = 0.9, BS = 67) *Pseudopodospermum* and an extended *Takhtajaniantha* clade. Further details will be treated in the Discussion below.

Phylogenetic reconstruction based on the concatenated plastid DNA markers

**Figure 2.**
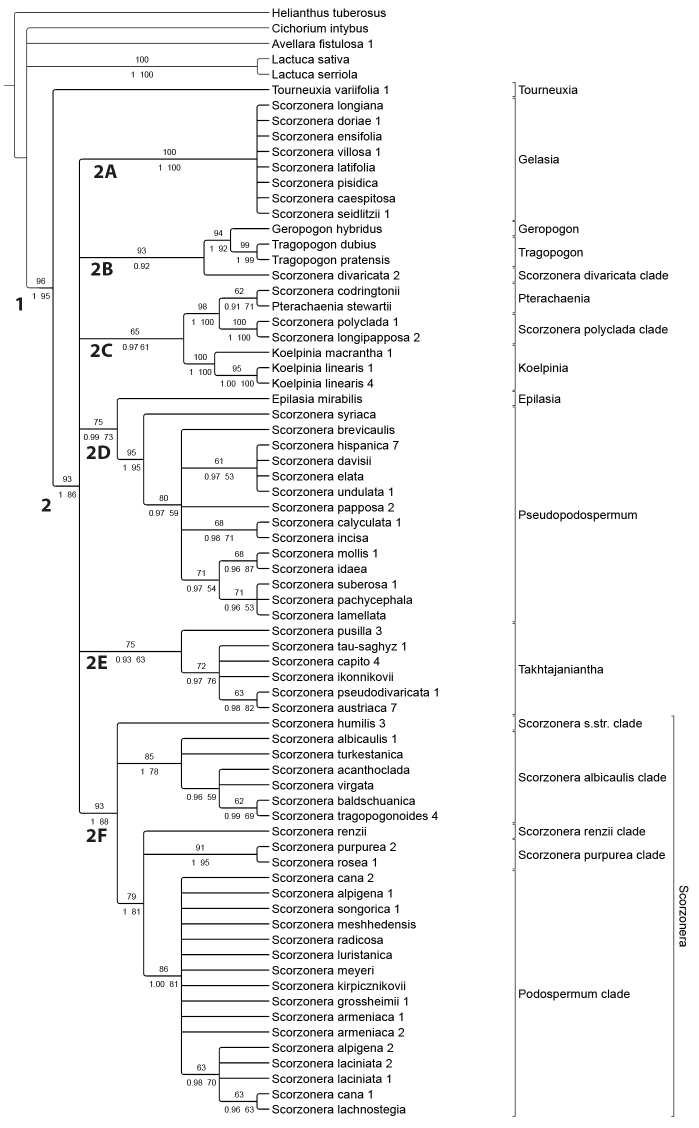
Majority consensus cladogram of the *Scorzonera* s.l. from the Bayesian analysis, based on the plastid DNA dataset (support values: Maximum Parsimony jackknife, second line: Bayesian posterior probability and Maximum Likelihood bootstrap) with clade designations. Designation of terminal clades corresponds to Fig. 1.

The alignment of the two concatenated plastid DNA markers had a length of 1387 characters, of which 112 were parsimony-informative. The MP analysis resulted in 103 most parsimonious trees (L = 371, CI = 0.730, RI = 0.857, RC = 0.626, HI = 0.270). The 50% majority consensus tree was largely congruent in topology with the 50% majority consensus trees of the BI and ML analyses. Fig. 2 shows the BI cladogram with the MP jackknife (JK) support values above and the BI posterior probabilities (PP) and ML bootstrap (BS) support values below the branches.

The Scorzonerinae are resolved as monophyletic and *Tourneuxia* as sister to the remainder of the subtribe with strong support (clade 1; JK = 96, PP = 1, BS = 95). The latter clade 2 is a polytomy: clade 2A with almost full support includes the *Gelasia* clade; clade 2B with strong support only in MP includes *Geropogon*, *Tragopogon* and the *Scorzonera divaricata* clade; clade 2C with low support (JK = 65, PP = 0.97, BS = 61) includes *Pterachaenia* with *S. codringtonii*, the *S. polyclada* clade and *Koelpinia*; clade 2D includes with moderate support (JK = 75, PP = 0.99, BS = 73) *Pseudopodospermum* with *Epilasia* as sister; clade 2E includes a moderately supported (JK = 75, PP = 93, BS = 63) *Takhtajaniantha* clade and the moderately strong supported clade 2F includes both *Scorzonera* in the sense of the type and *Podospermum*.

### Incongruences between nrDNA and plastid DNA phylogenies

Fewer of the deep nodes are resolved in the plastid DNA tree compared to the nrITS tree, but both reconstructions revealed largely the same major terminal clades. Statistically significant topological differences are few. They mainly concern the relationships of three clades: (1) *Epilasia* appears as sister to a clade combining *Geropogon* and *Tragopogon* in the nrITS tree, whereas as sister to *Pseudopodospermum* in the plastid DNA tree; (2) *Scorzonera divaricata* is sister to the *Tragopogon*-*Geropogon* clade in the plastid tree, but nested in a polytomy in the nrITS tree without the latter clade; (3) the *Scorzonera purpurea-S. renzii* clades are nested in the nrITS tree in a polytomy with *S. angustifolia* and the *S. albicaulis* clade, but in the plastid DNA tree, these two species are found in a polytomy with the *Podospermum* clade.

### Morphological and anatomical characters of the achene in the Scorzonerinae

The capitula of the Scorzonerinae are, in principle, homocarpic. Minor differentiations corresponding to the centripetal development of the florets and thus also achenes in the Asteraceae capitula may, however, occur. The marginal achenes are considered as the most representative type. Achene wall anatomy and morphology are fully developed in the middle third of the achene as in all Asteraceae. Correspondingly, all features refer to the middle third of the marginal achenes.

**Figure 3.**
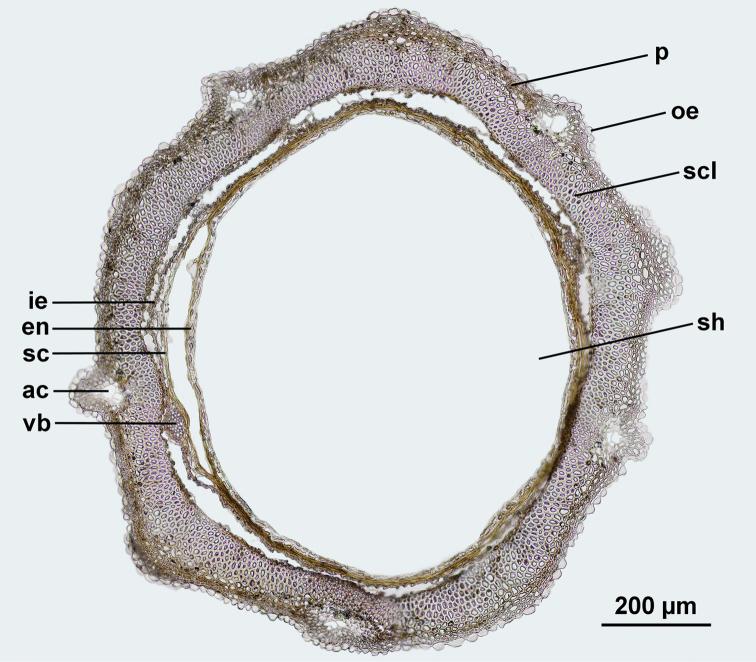
Cross-section of the middle achene part of *Scorzonera stricta* (from *V. Prima 485* (LE)). Abbreviations in pericarp: oe – outer epidermis, ac – air cavity, p – parenchyma, scl – sclerenchyma, vb – vascular bundles, ie – inner epidermis; abbreviations in seed: sc – seed coat, en – endosperm, sh – seed hollow (embryo not shown).

The achene wall (Fig. 3) is composed of five segments corresponding to the five principal vascular bundles of the achene wall (plesiomorphic state in all Cichorieae and all Compositae) that may be barely noticeable. The principal vascular bundles of the achene wall can be seen often well below the principal ribs even if the latter are not clearly expressed. In the Scorzonerinae, each segment forms one main rib and, usually, two secondary ribs, the latter shared with the contiguous segments, resulting in a pattern of 10 ribs, either differentiated or not into more prominent principal and less prominent secondary ribs. The achene epidermis is the outer cell layer of the achene wall and can be glabrous, hairy or papillose. The achene surface can also exhibit emergences: stout cell conglomerations, hooked spines or tubercles with verrucose ribs consisting of pore parenchyma. Below the epidermis, the achene wall is composed of sclerenchymatous and parenchymatous layers of various arrangements and thickness. Sometimes, air cavities are present, resulting from rupture of thin-walled parenchyma. The endocarp is often obliterated. The seed coat is ± adjoining to the endocarp and consists of two or several layers, sometimes with the vestiges of vascular bundles. The endosperm is two-layered and the vertical embryo occupies almost all volume in the achene corpus.

The morphological characters of the pappus, as part of the diaspore, are also included in the carpological analysis.

### Coding of carpological characters and states in Scorzonerinae

The carpologial analysis across the subtribe revealed a variety of features, which were found to be diagnostic for species or groups of them. In the following, these carpological features are coded in characters (composed of a structure and a property) and states. In the character designation, structures and substructures are separated by colons and these are separated from the respective property by a semicolon.

1. Achene: carpopodium (formed by the tube-like protruding achene wall); presence

0: absent

1: present

Carpopodia as a protrusion of the basal achene wall surrounding the stipe-like structure in which the vascular bundles of the achene enter the receptacle, are known throughout the family and the tribe (Haque and Godward 1984). A particular conspicuous tube-like protrusion of the achene wall is present in some members of *Scorzonera* s.l. (entirely omitted by Haque and Godward 1984) and has been employed in the past as a diagnostic character to delimit taxa at infrageneric or generic rank (S. subg. Podospermum, S. sect. Purpurea and *Pseudopodospermum*).

2. Achene: carpopodium: border (by shape and surface texture) between achene corpus and carpopodium; presence

0: absent (Fig. 4A)

**Figure 4.**
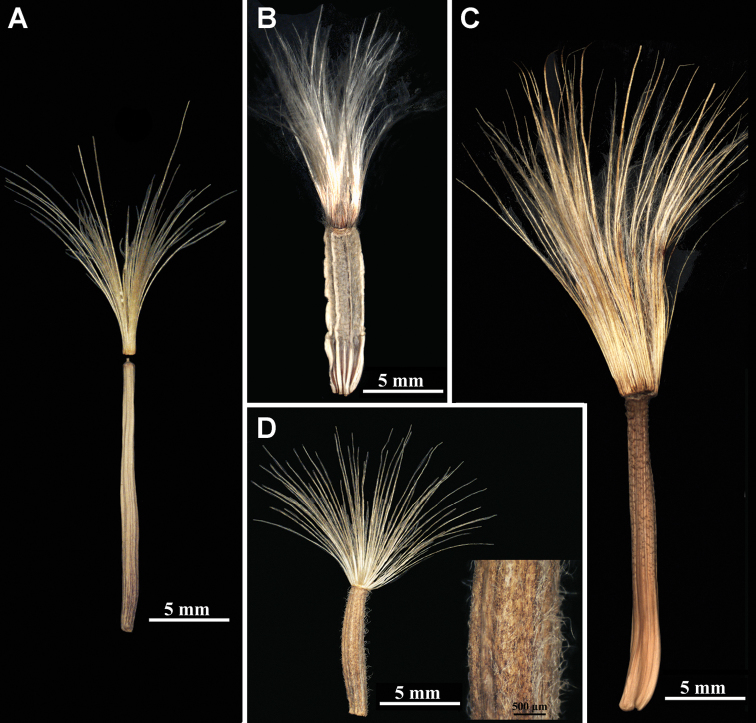
**A** Marginal achene of *Scorzonera transiliensis* without a carpopodium, **A** from *Lipschitz 271* (MW0892149) **B** marginal achene of *S. armeniaca* showing the carpopodium with a border, **B** from *Kuthatheladze s.n.* (LE) **C** marginal achene of *S. brevicaulis* showing the carpopodium without a border to the basal part of the achene, **C** from *Dubuis 12704* (B) **D** marginal achene of *S. ikonnikovii* (left: general view, right: a part of the achene with hairs), **D** from *Gubanov s.n*. (MW0194380).

1: present (Fig. 4B, C)

We note here for the first time that the carpopodium is morphologically separated from the achene corpus by a border in Scorzonera subg. Podospermum and some members of S. subg. Pseudopodospermum

3. Achene epidermis: hairs; presence

0: none, epidermis glabrous

1: with soft multicellular eglandular hairs (Fig. 4D)

**Figure 5.**
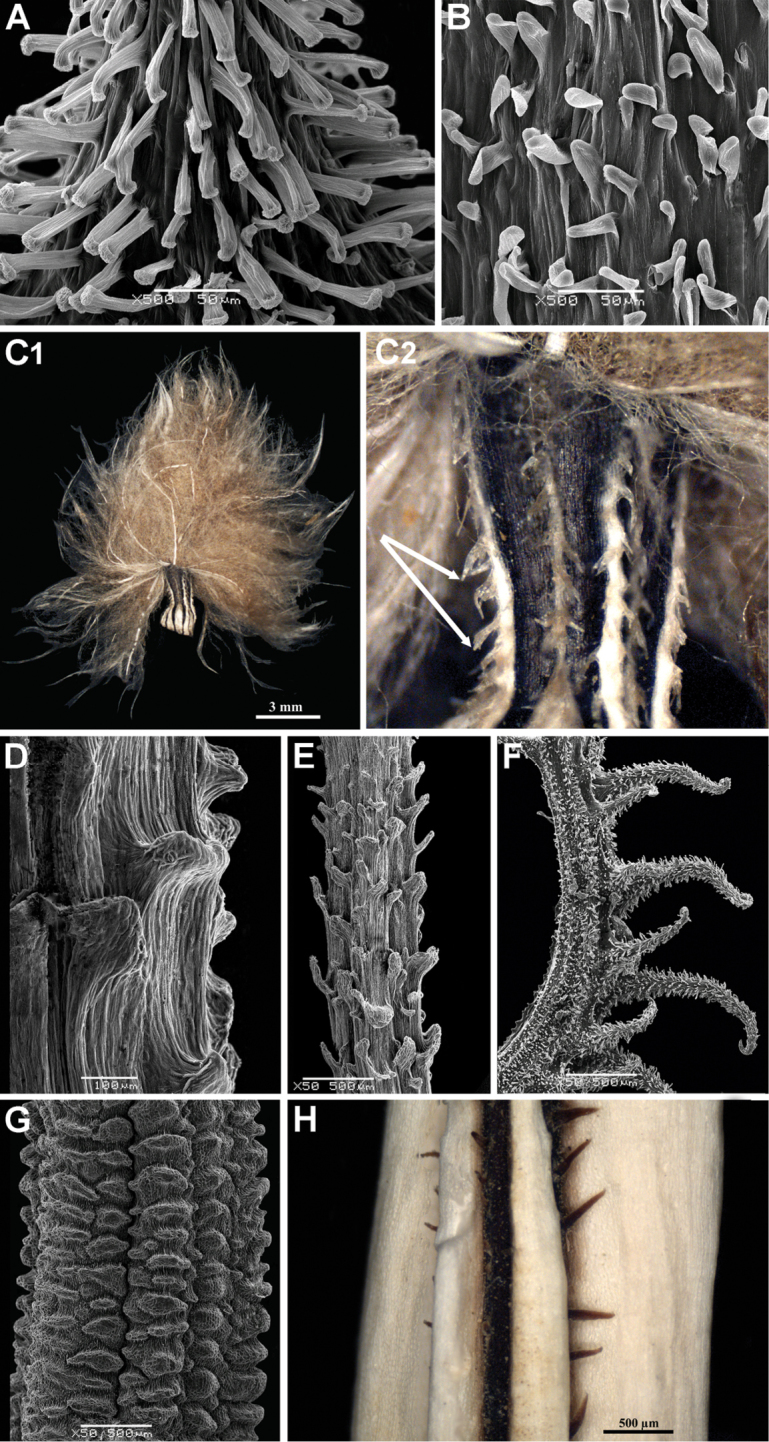
**A** SEM image with the close-up view of the achene body of *Koelpinia macrantha*, showing elongated slightly curved papillae, **A** from *Pimenov et al. 447* (MW0891267) **B** SEM image with the close-up view of the marginal achene of *Scorzonera pseudodivaricata* with cylindrical papillae, **B** from *Gubanov 8816* (MW0194378) **C1, C2** image of the marginal achene of *Epilasia hemilasia* (left: general view, right: close-up view of the achene body with stout hair-like papillae [arrows]), **C** from *Aidarova s.n.* (FRU). Continued. **D** SEM image of the marginal achene body of *S. pygmaea* showing stout and short conglomerations, **D** from *Quer 726* (B) **E** SEM image of the marginal achene body of *S. inconspicua* showing stout and short conglomerations, **E** from *Nikitin s.n.* (FRU) **F** SEM image of the marginal achene body of *Koelpinia macrantha* showing hooked spines on its surface. Each spine is covered with the elongated papillae, see also Fig. 4A). **F** from *Pimenov et al. 447* (MW0891267) **G** close-up view of the achene body of *S. aristata* with verrucose ultrasculpture, **G** from *Bornmüller s.n.* (B) **H** close-up view of the achene body of *Pterachaenia stewartii* with a spinula), **H** from *Anders 8990* (W0032145).

2: with drastically elongated, often slightly curved papillae looking like glandular hairs (Fig. 5A)

3: with cylindrical, not drastically elongated papillae or mamillae (Fig. 5B)

4: with retrorse acute papillae (Fig. 5C1, C2)

The outgrowths (hairs, papillae and mamillae), if present, originate from the achene epidermis. The hairs are multicellular in contrast to papillae (elongated cylindrical outgrowths) and mamillae (very small conical outgrowths) that are always unicellular. The character states 2 and 4 are peculiar for *Koelpinia* and *Epilasia*, respectively.

4. Achene surface: emergences; presence

0: absent

1: present

5. Achene surface: emergences; shape (Fig. 5)

0: stout and short conglomerations (Fig. 5D, E)

1: as hooked spines (*Koelpinia*: Fig. 5F)

2: as verrucose and undulate sculptures formed by pore parenchyma (Fig. 5G)

3: as spinules (*Pterachaenia stewartii*: Fig. 5H)

Emergences are distinguished from hairs and papillae as outgrowths of the achene wall originating from pericarp layers below the epidermis.

6. Achene wall: sclerenchyma; arrangement

0: Continuous sclerenchymatous layers (sheath) equal in thickness present

1: Discontinuous sclerenchymatous layers with a gap in the principal ribs

2: Continuous sclerenchymatous layers with a narrow or wider hunch on either side of the principal rib

3: Continuous or slightly discontinuous sclerenchyma with well-expressed invaginations (depressions).

Sclerenchyma as stabilising element develops in the achene wall above the vascular bundles into different patterns and formations as defined in the states coding.

7. Achene wall: sclerenchyma; orientation

0: Sclerenchyma entirely with parallel orientation to achene axis

1: Sclerenchyma differentiated: outer layer with parallel, inner with oblique to perpendicular orientation to achene axis.

2: Sclerenchyma only in emergences or rib areas perpendicular (or oblique) to achene axis.

**Figure 6.**
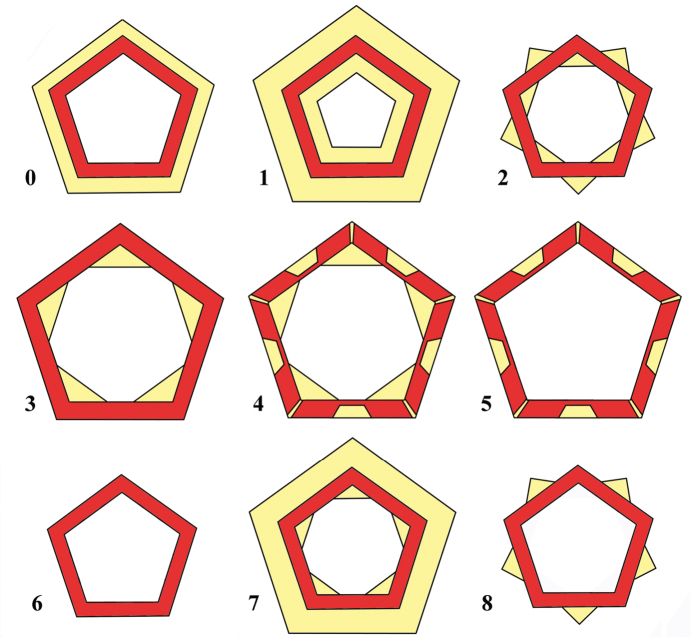
Various arrangements of the parenchyma in the pericarp. Numbers correspond with the states of the character 8. Yellow area – parenchyma, red area – sclerenchyma, white area in the centre – seed hollow.

8. Achene wall: parenchyma; arrangement (Fig. 6)

0: Present as subepidermal continuous layer(s)

1: Continuous parenchyma layers above and below the sclerenchyma

2: Insular in principal ribs below sclerenchyma and in secondary ribs above sclerenchyma

3: Only insular in principal ribs below sclerenchyma

4: Insular above sclerenchymatous invaginations and below sclerenchyma in the principal ribs

5: Only insular above sclerenchymatous invaginations

6: Absent

7: Parenchyma continuous above sclerenchyma and below sclerenchyma insular in principal ribs

8: Only insular in second ribs above sclerenchyma

Besides sclerenchyma, the main element of the achene wall is parenchyma, but often the parenchyma elements are left behind in quantity by the sclerenchyma. The distribution of the parenchyma elements in the achene wall is taxon specific and several different arrangements have been found as outlined in the character state coding. The various ways the parenchyma can be arranged in the achene wall is schematically shown in Fig. 6. As in the other cases, we have refrained from speculations about evolutionary pathways for these arrangements and have coded them as unordered states.

9. Achene wall: parenchyma; differentiation

0: Parenchyma only as subepidermal mechanical parenchyma

1: Subepidermal collenchyma-like layers differentiated besides parenchyma

2: Parenchyma of thin-walled cells only

3: Parenchyma of two types present, mechanical parenchyma and such with thin-walled cells

The unusual collenchyma-like layers are built of thin-walled cells with prominent intercellular spaces of triangular shape. The parenchyma in character states 0 and 2–3 lack prominent intercellular spaces.

10. Achene wall: air cavities; occurrence

0: Absent

1: Present

11. Achene wall; ribbing pattern

0: Each segment with a principal rib and 2 secondary ribs, the latter shared with the contiguous segments, achene middle third, thus with 5 principal and 5 secondary ribs

1: Each segment with a principal rib only, achene middle third, thus with 5 ribs

2: No distinct ribs developed, middle third ± terete (roundish)

3: Two or more ribs enlarged to wings

12. Achene wall: tannins; presence

0: Absent

1: Present in cell protoplast

2: Present in cell walls only

13. Achene beak; presence

0: Absent

1: Present

The beak is defined as the more or less abrupt attenuation of the achene apex into a stipe-like structure carrying the pappus disc.

14. Pappus; presence

0: Absent

1: Present

15. Pappus; structure

0: Of entirely softly plumose bristles (with long soft fimbriae all along the bristle)

1: Of entirely softly plumose bristles and scabrid awns

2: Of bristles softly plumose in lower and scabrid in upper half or third (at least 5–10 longer bristles have unequal lateral fimbriae)

4: Bristles scabrid completely or for the most part

The setaceous pappus in the Cichorieae consists of bristles with lateral projection (= fimbriae) not or little exceeding the diameter of the bristles, which make the bristles rough, thus “scabrid”. Alternatively, the lateral projection may be many times longer than the bristle diameter and the bristles thus featherlike or “plumose”. Plumose pappi in the Cichorieae have evolved as three different types, each specific for one subtribe (Kilian et al. 2009a). The plumose bristles in the Scorzonerinae are characterised by soft and often intertwining fimbriae pointing in all directions and consisting of a row of flattened cells. Taxon specific variation of the pappus structure concerns the composition of the pappus by plumose and scabrid elements or the differentiation of the fimbriae development in one and the same pappus element.

16. Pappus; colour

0: white

1: dirty white

2: fulvous (at least in basal part)

3: yellowish

4: grey or blackish

### Carpological characterisation of the clades and ancestral character reconstruction

**Figure 7.**
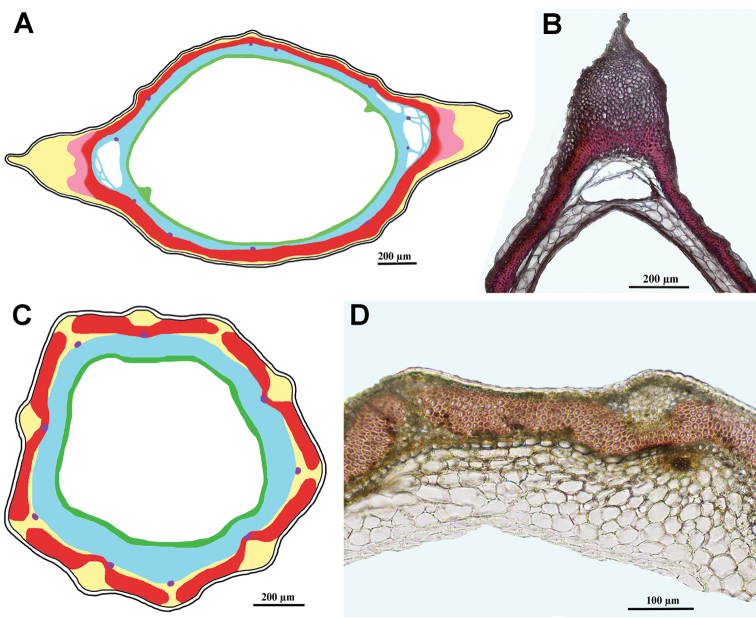
Schemes (**A, C**) and cross-sections of the achenes (**B, D**). **A, B***Tourneuxia variifolia* (*Tourneuxia* clade), **B** from *Bochantsev 862* (LE) **C, D***Scorzonera villosa* (*Gelasia* clade), **D** from *Rechinger 23425* (B). Designation of the colours in **A** and **C**: yellow – thin-walled parenchyma, pink (**A**) – sclerenchyma obliquely orientated in the region of the wings; red – sclerenchyma (of parallel orientation); blue – seed coat; violet dots (**C**) – vestiges of vascular bundles in the seed coat; green – endosperm; white areas between pericarp and seed coat designate air cavities; central white area – seed hollow.

**Figure 8.**
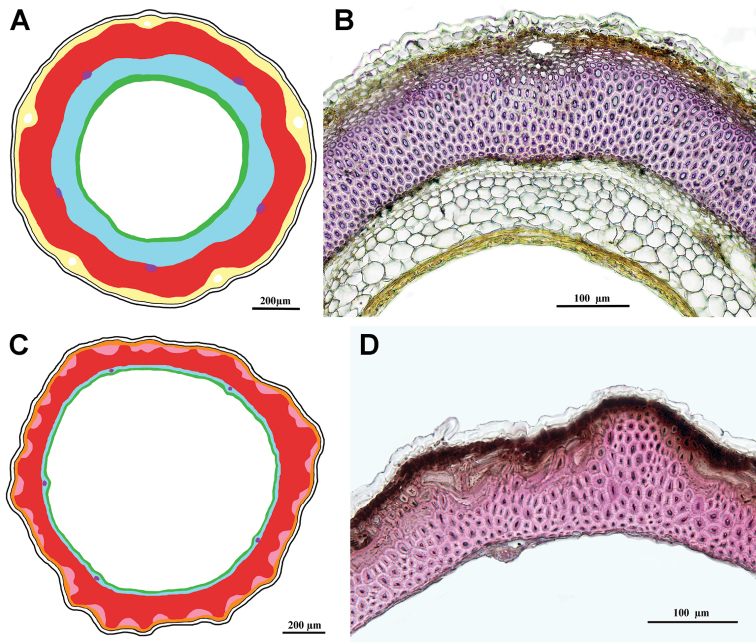
Schemes (**A, C**) and cross-sections of the achenes (**B, D**). **A, B***Geropogon hybridus* (*Geropogon* clade), **B** from *Seregin & Privalova A-692* (MW0752412) **C, D***Epilasia mirabilis* (*Epilasia* clade), **D** from *Furse 5920* (LE). Designation of the colours in **A** and **C**: yellow – thin-walled parenchyma, orange – mechanical subepidermal parenchyma, pink (**C**) – sclerenchyma obliquely orientated in the region of the wings; red – sclerenchyma (of parallel orientation); blue – seed coat (large in *Geropogon* and tiny in *Epilasia*); violet dots (**A, C**) – vestiges of vascular bundles in the seed coat; green – endosperm; small white areas in (**A**) – air cavities; central white area – seed hollow.

**Figure 9.**
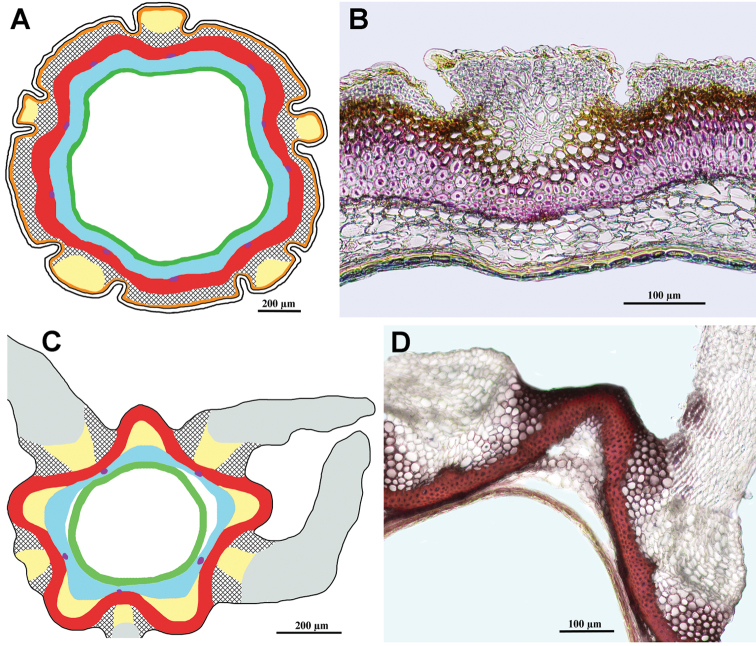
Schemes (**A, C**) and cross-sections of the achenes (**B, D**). **A, B***Scorzonera divaricata* (*Scorzonera divaricata* clade), **B** from *Gubanov 8585* (MW0194347) **C, D***Pterachaenia stewartii* (*Pterachaenia* clade), **D** from *Anders 8990* (W0032145). Designation of the colours in **A** and **C**: orange (**A**) – mechanical subepidermal sheath; grey (**C**) – wings; yellow – thin-walled parenchyma; hatched areas (**A, C**) – thick-walled (lignified) parenchyma; red – sclerenchyma (of parallel orientation); blue – seed coat; violet dots (**A, C**) – vestiges of vascular bundles in the seed coat; green – endosperm; central white area – seed hollow.

**Figure 10.**
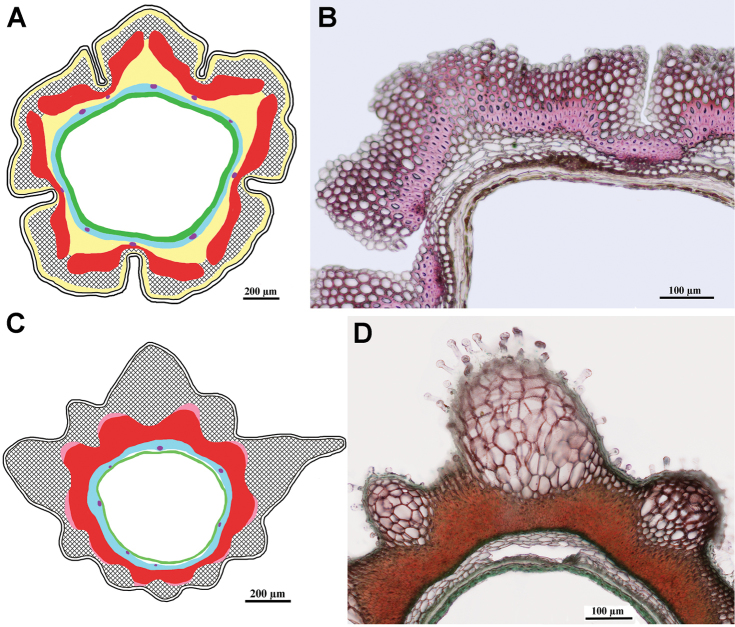
Schemes (**A, C**) and cross-sections of the achenes (**B, D**). **A, B***Scorzonera longipapposa* (*Scorzonera polyclada* clade), **B** from *Rechinger 35489* (G181688) **C, D***Koelpinia macrantha* (*Koelpinia* clade), **D** from *Pimenov et al. 447* (MW0891267). Designation of the colours: yellow – thin-walled parenchyma (absent in **C**), hatched area – thick-walled parenchyma; pink (**C**) – sclerenchyma obliquely orientated in the region of the wings (present in **C**); red – sclerenchyma (of parallel orientation); blue – seed coat; violet dots (**A, C**) – vestiges of vascular bundles in the pericarp and seed coat (**A**) and in the seed coat (**C**); green – endosperm; central white area – seed hollow.

**Figure 11.**
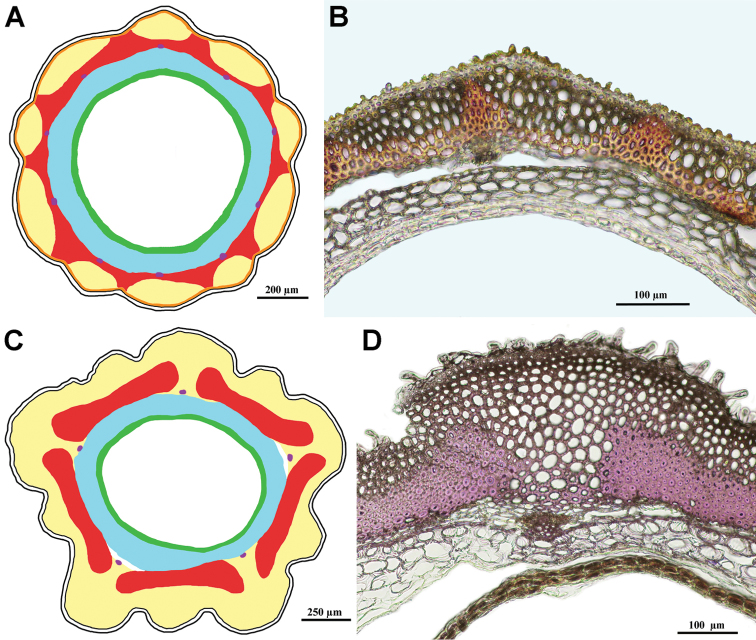
Schemes (**A, C**) and cross-sections of the achenes (**B, D**), *Takhtajaniantha* clade. **A, B***Scorzonera pusilla* (Takhtajaniantha type), **B** from *Aidarova s.n.* (FRU) **C, D***S. pseudodivaricata* (Pseudodivaricata type), **D** from *Gubanov 8816* (MW0194378). Designation of the colours: orange (**A**) – mechanical subepidermal parenchyma; yellow – thin-walled parenchyma; red – sclerenchyma (of parallel orientation); blue – seed coat; violet dots – vestiges of vascular bundles in the seed coat; green – endosperm; central white area – seed hollow.

**Figure 12.**
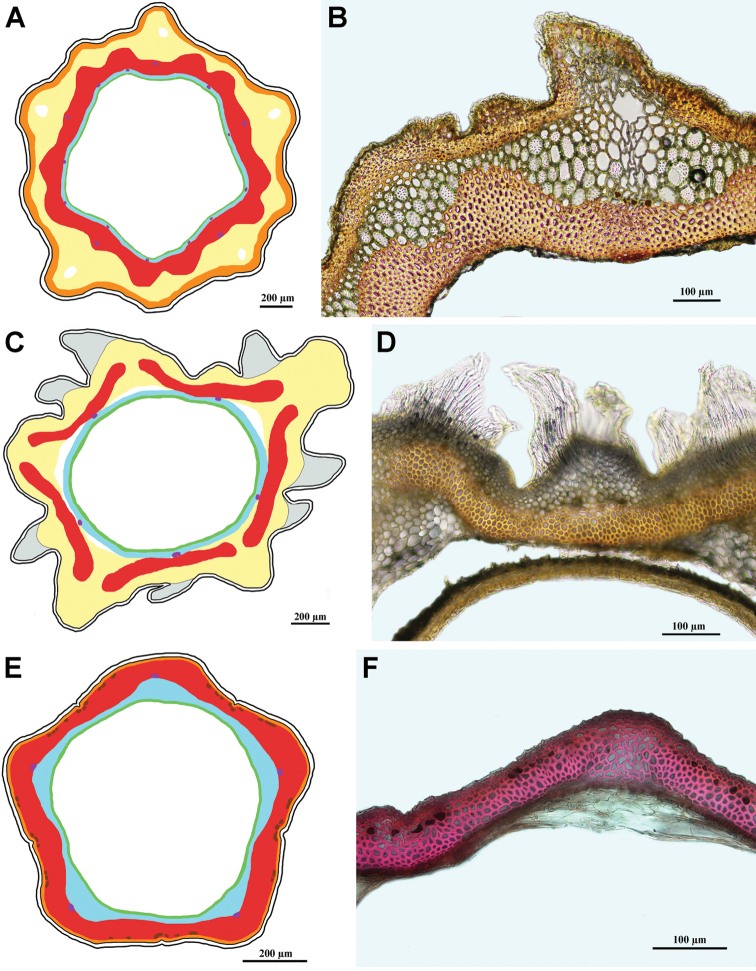
Schemes (**A, C, E**) and cross-sections of the achenes (**B, D, F**), *Pseudopodospermum* clade, **A, B***Scorzonera brevicaulis* (Brevicaulis type), **B** from *Dubuis 12704* (B) **C, D***S. papposa* (Calyculata type), **D** from *Bornmüller 4131* (B) **E, F***S. hispanica* (Pseudopodospermum type). F from *Rechinger 1590* (B100047704). Designation of the colours: orange (**A, E**) – mechanical subepidermal parenchyma; yellow – thin-walled parenchyma, white small hollows in the pericarp – air cavities; grey – stout conglomerations (**C**); red – sclerenchyma (of parallel orientation); blue – seed coat; green – endosperm; violet dots in pericarp – tannins cells, violet dots in seed coat – vestiges of vascular bundles; central white area – seed hollow.

**Figure 13.**
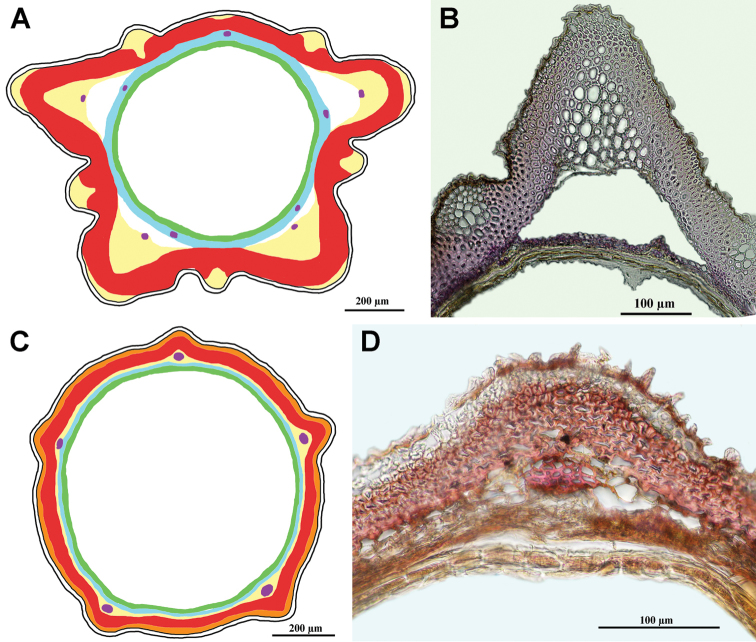
Schemes (**A, C**) and cross-sections of the achenes (**B, D**). **A, B***Scorzonera humilis* (*Scorzonera* clade), **B** from *Tikhomirov & al. s.n.* (MW0550805) **C, D***S. rhodantha* (*S. purpurea* clade), **D** from *Rechinger 18566* (B). Designation of the colours in **A** and **C** orange (**C**) – mechanical subepidermal parenchyma; yellow – thin-walled parenchyma (very small in **C** below the sclerenchyma); red – sclerenchyma (of parallel orientation); blue – seed coat; violet dots (**A, C**) – vestiges of vascular bundles in both pericarp and seed coat (**A**) and in the pericarp (**C**); green – endosperm; central white area – seed hollow.

**Figure 14.**
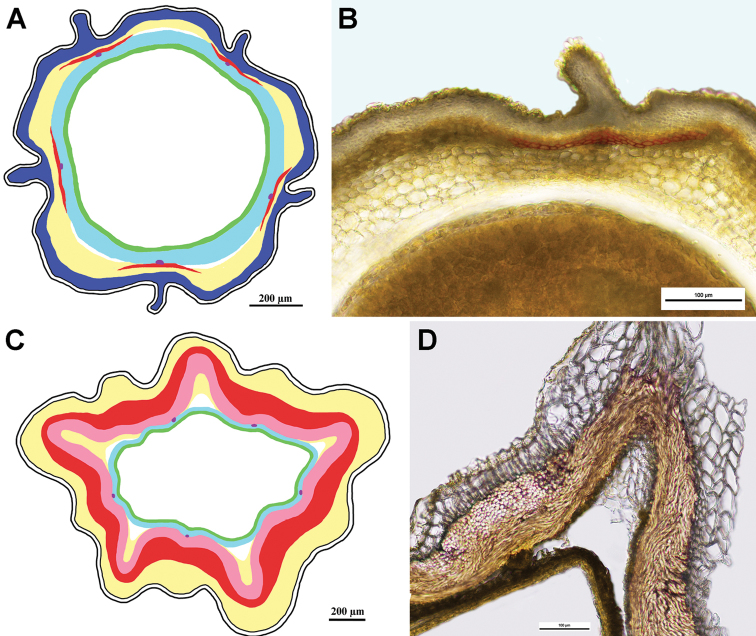
Schemes (**A, C**) and cross-sections of the achenes (**B, D**). **A, B***Scorzonera tragopogonoides* (Piptopogon type), **B** from *Kryltsov s.n.* (MW0891887) **C, D***S. armeniaca* (Podospermum type), **D** from *Kuthatheladze s.n.* (LE). Designation of the colours in **A** and **C**: yellow – thin-walled parenchyma; red – sclerenchyma (of parallel orientation); pink – sclerenchyma (of perpendicular orientation); dark blue – collenchymatous parenchyma (**A**); blue – seed coat; violet dots (**A, C**) – vestiges of vascular bundles in the seed coat (**A**) and in the pericarp (**C**); green – endosperm; central white area – seed hollow.

The matrix of the 16 carpological characters is given in Table 2. Representative achene cross sections photographs and schemes representative for the clades are presented in Figs 7–14. The detailed carpological descriptions of the principal terminal clades are presented in the concluding part “Taxonomy”, below. Here, we summarise the anatomical features for the individual clades.

**Table 2.** Matrix of the carpological characters. Numerical designations of characters and states refer to the corresponding section of the Results. * – sample included in the ITS tree, # – sample included in the plastid DNA tree, clear text – sample only included in the carpological analysis.

Taxon [sample]/ character	1	2	3	4	5	6	7	8	9	10	11	12	13	14	15	16
*Tourneuxia* clade																
*^#^*Tourneuxia variifolia* [-1, -3]	0	n/a	0	0	n/a	0	2	0	2	0	3	0	0	1	2	2
*Gelasia* clade																
*Scorzonera acantholimon*	0	n/a	0	0	n/a	3	0	1	2	0	1	0	0	1	2	2
*Scorzonera albicans*	0	n/a	1	0	n/a	3	0	2	2	0	2	0	0	1	2	2
*Scorzonera araneosa*	0	n/a	1	0	n/a	3	0	2	2	0	2	0	0	1	4	2
*Scorzonera biebersteinii*	0	n/a	1	0	n/a	3	0	1	2	0	1	0	0	1	2	2
^#^ *Scorzonera caespitosa*	0	n/a	0	1	0	3	0	2	2	0	2	0	0	1	2	2
*Scorzonera cinerea [-1]	0	n/a	0	0	n/a	3	0	1	2	0	1	0	0	1	2	2
*Scorzonera circumflexa [-1]	0	n/a	1	0	n/a	3	0	1	2	0	1	0	0	1	2	2
*Scorzonera cretica [-1]	0	n/a	1	0	n/a	3	0	1	n/a	0	1	0	0	1	4	3
*Scorzonera czerepanovii*	0	n/a	1	0	n/a	3	0	1	2	0	1	0	0	1	2	3
*^#^*Scorzonera doriae* [-1]	0	n/a	1	0	n/a	3	0	2	2	0	2	0	0	1	2	2
*Scorzonera dzhawakhetica* [-1]	0	n/a	1	0	n/a	3	0	1	2	0	1	0	0	1	2	2
*Scorzonera dzhawakhetica* [-2]	0	n/a	1	0	n/a	3	0	1	2	0	1	0	0	1	2	2
^#^ *Scorzonera ensifolia*	0	n/a	1	0	n/a	3	0	1	2	0	1	0	0	1	2	3
*Scorzonera eriophora [-1]	0	n/a	1	0	n/a	3	0	2	2	0	2	0	0	1	2	2
*Scorzonera filifolia*	0	n/a	0	0	n/a	3	0	1	2	0	1	0	0	1	4	2
*Scorzonera hirsuta [-1]	0	n/a	1	0	n/a	3	0	1	2	0	1	0	0	1	2	2
Scorzonera hirsuta var. villosaeformis	0	n/a	1	0	n/a	3	0	1	2	0	1	0	0	1	2	2
*Scorzonera ketzkhovelii*	0	n/a	0	0	n/a	3	0	1	2	0	1	0	0	1	2	3
*Scorzonera kotschyi [-2]	0	n/a	1	0	n/a	3	0	1	2	0	1	0	0	1	2	2
*^#^*Scorzonera latifolia* [-1, - 2]	0	n/a	1	0	n/a	3	0	1	2	0	1	0	0	1	2	2
*Scorzonera mirabilis*	0	n/a	0	0	n/a	3	0	1	2	0	1	0	0	1	4	3
*Scorzonera psychrophila [-1, -3]	0	n/a	1	0	n/a	3	0	2	2	0	2	0	0	1	2	3
*Scorzonera pygmaea [-1]	0	n/a	0	1	0	3	0	2	2	0	2	0	0	1	4	2
*Scorzonera ramosissima*	0	n/a	1	0	n/a	3	0	2	2	0	2	0	0	1	2	1
*Scorzonera rigida [-1]	0	n/a	0	0	n/a	3	0	1	2	0	1	0	0	1	2	1
*^#^*Scorzonera seidlitzii* [-1, -2]	0	n/a	0	0	n/a	3	0	1	2	0	1	0	0	1	2	2
*Scorzonera sericea*	0	n/a	0	0	n/a	3	0	2	2	0	2	0	0	1	2	2
*Scorzonera tomentosa*	0	n/a	0	0	n/a	3	0	2	2	0	2	0	0	1	4	2
*Scorzonera ulrichii*	0	n/a	1	0	n/a	3	0	2	2	0	2	0	0	1	2	2
*^#^*Scorzonera villosa* [-1]	0	n/a	0	1	0	3	0	1	2	0	1	0	0	1	4	2
*Epilasia* clade																
*Epilasia acrolasia [-1, -3]	1	1	4	0	n/a	0	0	0	0	0	2	2	0	1	2	4
*Epilasia hemilasia [-1]	1	1	4	0	n/a	0	0	0	0	0	2	2	0	1	2	4
*^#^*Epilasia mirabilis*	1	1	4	0	n/a	0	2	0	0	0	0	2	0	1	2	4
*Geropogon* clade																
*^#^*Geropogon hybridus*	0	n/a	0	1	0	0	0	0	2	1	2	2	1	1	1	1
Tragopogon clade																
*Tragopogon brevirostris	0	n/a	0	1	0	0	0	0	3	0	2	0	0	1	0	1
*Tragopogon porrifolius	0	n/a	0	1	0	0	0	0	3	1	1	0	1	1	0	1
*^#^*Tragopogon dubius*	0	n/a	0	1	0	0	0	0	3	1	1	0	1	1	0	1
*Divaricata* clade																
*^#^*Scorzonera divaricata* [-1, -2, -3]	0	n/a	3	0	n/a	0	0	0	3	0	0	2	0	1	2	1
*Pterachaenia* clade																
*^#^*Pterachaenia stewartii* [-1, -4]	0	n/a	3	1	3	0	0	2	3	0	3	0	0	1	2	2
*^#^*Scorzonera codringtonii* [-1, -2]	0	n/a	0	0	n/a	0	0	5	3	0	1	0	0	1	2	2
*Polyclada* clade																
*Scorzonera intricata [-1]	0	n/a	0	0	n/a	3	0	7	3	0	0	0	0	1	2	2
*Scorzonera koelpinioides*	0	n/a	0	0	n/a	3	0	7	3	0	0	2	0	1	2	1
*^#^*Scorzonera longipapposa* [-1, -2]	0	n/a	0	0	n/a	3	0	7	3	0	1	0	0	1	2	1
*Scorzonera musilii*	0	n/a	0	0	n/a	3	0	7	3	0	0	0	0	1	2	2
*^#^*Scorzonera polyclada* [-1, -2]	0	n/a	0	0	n/a	3	0	7	3	0	1	0	0	1	2	2
*Scorzonera tortuosissima*	0	n/a	0	0	n/a	3	0	7	3	0	0	0	0	1	2	2
*Koelpinia* clade																
*^#^*Koelpinia linearis* [-1]	0	n/a	2	1	1	0	2	0	0	1	0	0	0	0	n/a	n/a
*Koelpinia linearis [-4]	0	n/a	2	1	1	0	2	0	0	0	1	0	0	0	n/a	n/a
*^#^*Koelpinia macrantha* [-1]	0	n/a	2	1	1	2	2	0	0	0	0	0	0	0	n/a	n/a
*Koelpinia tenuissima*	0	n/a	3	0	n/a	0	0	0	0	1	0	0	0	0	n/a	n/a
*Takhtajaniantha* clade																
*Austriaca* type																
*^#^*Scorzonera austriaca* [-1, -2, -7]	0	n/a	3	0	n/a	2	0	0	3	0	0	0	0	1	2	1
Scorzonera austriaca subsp. curvata	0	n/a	3	0	n/a	2	0	0	3	0	0	0	0	1	2	1
Scorzonera austriaca var. verrucosa	0	n/a	3	1	0	2	0	0	3	0	0	0	0	1	2	1
Scorzonera austriaca var. tenuifolia	0	n/a	3	0	n/a	2	0	0	3	0	0	0	0	1	2	1
*Scorzonera crispa*	0	n/a	3	0	n/a	2	0	0	3	0	0	0	0	1	2	1
*^#^*Scorzonera ikonnikovii*	0	n/a	1	0	n/a	2	0	0	3	0	0	0	0	1	2	1
*Scorzonera mongolica [-3, -4]	0	n/a	3	0	n/a	2	0	0	3	0	0	0	0	1	2	0
*^#^*Scorzonera pusilla* [-1, -2, -3]	0	n/a	3	0	n/a	2	0	0	3	0	0	0	0	1	2	0
Scorzonera pusilla f. vegetor	0	n/a	3	1	0	2	0	0	3	0	0	0	0	1	2	0
*Pseudodivaricata* type																
*^#^*Scorzonera capito* [-1, -2, -3, -4]	0	n/a	1	1	0	0	0	0	2	0	0	0	0	1	2	0
*^#^*Scorzonera pseudodivaricata* [-1, -3, -4]	0	n/a	3	0	n/a	1	0	0	2	0	0	0	0	1	2	0
*^#^*Scorzonera tau-saghyz* [-1, -3]	0	n/a	1	1	0	0	0	6	n/a	0	0	0	0	1	2	0
*Scorzonera tau-saghyz* [-2]	0	n/a	1	0	n/a	0	0	8	2	0	0	0	0	1	2	0
*Scorzonera veresczaginii*	0	n/a	3	0	n/a	0	0	0	2	0	0	0	0	1	2	1
*Pseudopodospermum* clade																
*Calyculata* type																
*^#^*Scorzonera calyculata* [-1]	0	n/a	3	1	2	1	0	0	2	0	0	1	0	1	2	1
*Scorzonera crispatula [-1]	0	n/a	3	1	0	1	0	0	2	0	1	1	0	1	2	1
*^#^*Scorzonera davisii*	0	n/a	3	1	2	1	0	0	2	0	1	1	0	1	2	0
*Scorzonera hissarica	0	n/a	0	1	0	1	0	0	2	0	1	0	0	1	2	2
*Scorzonera inaequiscapa	0	n/a	3	1	0	1	0	0	2	0	1	1	0	1	2	1
*^#^*Scorzonera incisa*	0	n/a	3	1	2	1	0	0	2	0	1	0	0	1	2	1
*Scorzonera libanotica*	0	n/a	3	1	2	1	0	0	2	0	1	0	0	1	2	1
*Scorzonera ovata*	0	n/a	0	1	0	1	0	0	2	0	0	0	0	1	2	0
*Scorzonera papposa [-1, -4]	0	n/a	3	1	0	1	0	0	2	0	0	0	0	1	2	0
^#^*Scorzonera papposa* [-2]	0	n/a	3	1	0	1	0	0	2	0	1	1	0	1	2	0
*Scorzonera reverchonii*	0	n/a	3	1	2	1	0	0	2	0	2	1	0	1	2	1
*Scorzonera troodea*	0	n/a	3	1	2	1	0	0	2	0	1	1	0	1	2	0
*Scorzonera violacea*	0	n/a	3	1	2	1	0	0	2	0	0	1	0	1	2	1
*Pseudopodospermum* type																
*Scorzonera boetica*	1	1	0	0	n/a	0	0	0	3	1	1	1	0	1	2	1
*Scorzonera chantavica*	1	1	3	1	0	0	0	0	3	0	0	1	0	1	2	3
*^#^*Scorzonera elata*	1	1	3	1	0	0	0	0	3	0	1	1	0	1	2	0
*^#^*Scorzonera hispanica* [-4, -7]	0	n/a	3	1	0	0	0	0	3	0	1	1	0	1	2	1
*Scorzonera hispanica* [-8]	0	n/a	3	1	0	0	0	0	3	0	0	1	0	1	2	1
*Scorzonera hispanica* [-5]	0	n/a	3	0	n/a	0	0	0	0	0	1	1	0	1	2	1
*^#^*Scorzonera idaea*	1	1	3	0	n/a	0	0	0	0	0	1	0	0	1	2	1
*Scorzonera inconspicua* [-1, -2]	1	1	3	1	0	0	0	0	3	0	0	1	0	1	2	1
*Scorzonera gracilis*	0	n/a	3	1	0	0	0	0	3	0	1	1	0	1	2	1
*^#^*Scorzonera lamellata*	1	1	3	1	0	0	0	0	0	0	2	0	0	1	2	1
*Scorzonera leptophylla*	1	1	3	1	0	0	0	0	3	1	0	1	0	1	2	4
*^#^*Scorzonera mollis* [-1, -2]	1	1	3	1	0	0	0	0	0	0	1	1	0	1	2	0
*Scorzonera mollis* [-3]	1	1	3	1	0	0	0	0	3	0	1	1	0	1	2	1
*Scorzonera mollis var. platyphylla	1	1	3	1	0	0	0	0	3	0	2	1	0	1	2	1
*^#^*Scorzonera pachycephala*	1	1	3	1	0	0	0	0	0	0	1	1	0	1	2	4
*Scorzonera pubescens	1	1	3	1	0	0	0	0	0	0	2	1	0	1	2	1
*Scorzonera raddeana [-1]	0	n/a	3	1	0	0	0	0	0	0	2	0	0	1	2	4
*Scorzonera rawii*	0	n/a	3	1	0	1	0	0	3	1	0	1	0	1	2	4
*Scorzonera stricta*	1	1	3	1	0	0	0	0	3	1	0	1	0	1	2	1
*^#^*Scorzonera suberosa* [-1]	1	1	3	1	0	0	0	0	3	1	1	1	0	1	2	4
*Scorzonera szowitzii*	1	1	3	1	0	0	0	0	3	1	1	1	0	1	2	4
*Scorzonera turcomanica*	0	n/a	1	1	0	1	0	0	0	0	1	1	0	1	2	4
*Scorzonera semicana*	1	1	3	1	0	0	0	0	3	0	1	1	0	1	2	1
*^#^*Scorzonera undulata* [-1]	1	1	3	1	0	1	0	0	2	1	0	1	0	1	2	1
*Brevicaulis* type																
*Scorzonera brevicaulis* [-1, -2]	1	0	3	1	0	2	0	0	3	1	1	1	0	1	2	3
*Scorzonera crocifolia*	1	1	3	1	0	2	0	0	3	1	0	1	0	1	2	3
Scorzonera hispanica var. asphodeloides	0	n/a	3	1	0	2	0	0	3	1	1	1	0	1	2	1
*Scorzonera phaeopappa [-1]	1	0	3	1	0	2	0	0	3	0	2	0	0	1	2	3
*Scorzonera phaeopappa* [-2]	1	1	3	1	0	2	0	0	3	1	1	1	0	1	2	3
*^#^*Scorzonera syriaca*	1	0	3	1	0	2	0	0	3	1	1	1	0	1	2	3
*Scorzonera* s.str. clade																
*Scorzonera aristata [-3]	0	n/a	3	1	2	0	0	8	2	0	0	0	0	1	2	1
*^#^*Scorzonera humilis* [-3, -4]	0	n/a	3	0	n/a	0	0	2	2	0	0	0	0	1	2	1
Scorzonera humilis f. alpina	0	n/a	3	0	n/a	0	0	2	2	0	0	0	0	1	2	1
*Scorzonera parviflora [-1, -2]	0	n/a	3	0	n/a	0	0	2	2	0	0	0	0	1	2	1
*Scorzonera radiata*	0	n/a	3	0	n/a	0	0	2	2	0	0	0	0	1	2	1
*Purpurea* clade																
*^#^*Scorzonera purpurea* [-1]	1	1	3	0	n/a	0	0	1	3	0	2	0	0	1	2	1
*^#^*Scorzonera purpurea* [-2]	1	1	3	1	2	0	0	1	3	0	2	0	0	1	2	1
Scorzonera purpurea subsp. peristerica	1	1	3	0	n/a	0	0	1	3	0	1	0	0	1	2	1
*^#^*Scorzonera rosea* [-1, -3]	1	1	3	0	n/a	0	0	1	3	0	2	0	0	1	2	1
*Scorzonera rhodantha*	1	1	3	1	2	0	0	1	3	0	1	0	0	1	2	1
*Scorzonera albicaulis* clade																
*Achyroseris* type																
*Scorzonera angustifolia [-2]	0	n/a	3	0	n/a	1	0	0	1	0	0	0	1	1	2	3
*Scorzonera graminifolia*	0	n/a	3	0	n/a	1	0	0	1	0	0	0	1	1	2	3
*^#^*Scorzonera baldschuanica*	0	n/a	3	0	n/a	0	0	0	1	0	0	0	1	1	2	3
*Scorzonera bracteosa	0	n/a	3	0	n/a	1	0	0	1	0	0	0	1	1	2	3
*Scorzonera crassicaulis*	0	n/a	3	0	n/a	1	0	0	1	0	0	0	1	1	2	3
*Scorzonera petrovii*	0	n/a	3	0	n/a	1	0	0	1	0	0	0	1	1	2	3
*^#^*Scorzonera tragopogonoides* [-1, -4]	0	n/a	3	0	n/a	0	0	0	1	0	0	0	1	1	2	3
*Scorzonera tragopogonoides* [-2]	0	n/a	3	0	n/a	1	0	0	1	0	0	0	1	1	2	3
*Albicaulis* type																
*^#^*Scorzonera acanthoclada*	0	n/a	3	0	n/a	0	0	3	2	0	1	0	1	1	2	3
*^#^*Scorzonera albicaulis* [-1, -2]	0	n/a	3	0	n/a	0	0	2	2	0	0	0	1	1	2	3
*Scorzonera franchetii* [-1]	0	n/a	3	0	n/a	0	0	6	n/a	0	0	0	1	1	2	3
*Scorzonera franchetii* [-2, -3]	0	n/a	3	0	n/a	0	0	2	2	0	1	0	1	1	2	3
*Scorzonera iliensis*	0	n/a	3	0	n/a	0	0	6	n/a	0	1	0	1	1	2	3
*Scorzonera racemosa	0	n/a	3	0	n/a	0	0	6	n/a	0	1	0	1	1	2	3
*Scorzonera rupicola	0	n/a	3	0	n/a	0	0	3	2	0	0	0	1	1	2	3
*Scorzonera transiliensis* [-1, -2]	0	n/a	3	0	n/a	0	0	2	2	0	0	0	1	1	2	3
*^#^*Scorzonera turkestanica*	0	n/a	3	0	n/a	0	0	3	2	0	1	0	1	1	2	3
*Podospermum* clade																
*^#^*Scorzonera alpigena* [-1]	1	1	1	1	2	0	1	7	2	0	0	0	0	1	2	0
*^#^*Scorzonera alpigena* [-2]	1	1	1	1	2	0	1	7	2	0	3	0	0	1	2	0
*^#^*Scorzonera armeniaca* [-1, -4]	1	1	1	1	2	0	1	7	2	0	3	0	0	1	2	0
*^#^*Scorzonera armeniaca* [-2]	1	1	1	1	2	0	1	7	2	0	0	0	0	1	2	0
*Scorzonera armeniaca* [-3]	1	1	1	0	n/a	0	1	1	2	0	0	0	0	1	2	0
*^#^*Scorzonera cana* [-1, -2, -5]	1	1	0	0	n/a	0	1	7	2	0	0	0	0	1	2	1
*Scorzonera cana* [-6]	1	1	0	0	n/a	0	1	1	2	0	1	0	0	1	2	1
*^#^*Scorzonera grossheimii* [-1, -2]	1	1	0	0	n/a	0	1	1	2	0	0	0	0	1	2	1
*Scorzonera hieraciifolia [-1]	1	1	0	1	2	0	1	7	2	0	0	0	0	1	2	1
*Scorzonera kandavanica*	1	1	1	0	n/a	0	1	0	2	0	0	0	0	1	2	0
*^#^*Scorzonera kirpicznikovii*	1	1	0	0	n/a	0	1	1	2	0	1	0	0	1	2	1
*^#^*Scorzonera lachnostegia*	1	1	0	0	n/a	0	1	1	2	0	0	0	0	1	2	1
*^#^*Scorzonera laciniata* [-1, -9]	1	1	0	0	n/a	0	1	7	2	0	0	0	0	1	2	1
*^#^*Scorzonera laciniata* [-2]	1	1	0	0	n/a	0	1	0	2	0	0	0	0	1	2	1
*Scorzonera laciniata* [-6, -7]	1	1	0	0	n/a	0	1	1	2	0	0	0	0	1	2	1
*^#^*Scorzonera luristanica*	1	1	0	0	n/a	0	1	7	2	0	0	0	0	1	2	0
*^#^*Scorzonera meshhedensis*	1	1	0	0	n/a	0	1	7	2	0	2	0	0	1	2	1
*^#^*Scorzonera meyeri*	1	1	0	0	n/a	0	1	1	2	0	1	0	0	1	2	1
*Scorzonera persepolitana*	1	1	1	0	n/a	0	1	7	2	0	0	0	0	1	2	0
*^#^*Scorzonera radicosa*	1	1	0	0	n/a	0	1	1	2	0	2	0	0	1	2	1
*Scorzonera schischkinii*	1	1	0	0	n/a	0	1	1	2	0	0	0	0	1	2	1
*^#^*Scorzonera songorica* [-1, -2, -3]	1	1	0	0	n/a	0	1	7	2	0	0	0	0	1	2	1

***Tourneuxia* clade**

Fig. 7A, B

Achenes without carpopodium, with two ribs elongated into small wings; achene epidermis glabrous; emergences absent; subepidermal parenchyma almost continuous, more prominent in the winged areas and much thinner (1–3 layers) between them, of thin-walled cells only; sclerenchyma continuous, its fibres of parallel orientation, sometimes slightly obliquely orientated in the region of the wings; no air cavities; tannins absent.

***Gelasia* clade**

Fig. 7C, D

Achenes without carpopodium; achene epidermis often densely covered with soft multicellular hairs making a long woolly indumentum; emergences mostly absent; subepidermal parenchyma usually insular, of thin-walled cells only; sclerenchyma continuous with an invagination on either side of the principal rib, parallel; no air cavities; tannins absent.

***Geropogon* clade**

Fig. 8A, B

Achenes without carpopodium; achene epidermis with papillae, emergences present; subepidermal parenchyma continuous of thin-walled cells only; sclerenchyma continuous, parallel; air cavities present; tannins present.

***Tragopogon* clade**

Achenes without capopodium; achene epidermis usually with papillae; emergences absent or present; subepidermal parenchyma continuous, of thick-walled or thick- and thin-walled cells; sclerenchyma continuous, parallel; air cavities usually present; tannins absent.

***Epilasia* clade**

Fig. 8C, D

Achenes with carpopodium; achene wall with hair-like papillae; emergences absent; subepidermal parenchyma continuous, of thick-walled cells only; sclerenchyma continuous, parallel; air cavities absent; tannins present.

***Scorzonera divaricata* clade**

Fig. 9A, B

Achenes without carpopodium; achene epidermis with papillae; emergences absent; subepidermal parenchyma of both thin- and thick-walled cells; sclerenchyma continuous, parallel; air cavities absent; tannins present.

***Pterachaenia* clade**

Fig. 9C, D

Achenes without carpopodium; with 2–3 elongated ribs forming wings (*Pterachaenia stewartii*) or without (*Pterachaenia codringtonii*); achene epidermis with papillae or glabrous; emergences absent; subepidermal parenchyma discontinuous, located above sclerenchyma between the principal ribs and below sclerenchyma in the rib areas or only insular above sclerenchyma, of both thin- and thick-walled cells; sclerenchyma continuous, parallel; air cavities absent; tannins absent.

***Scorzonera polyclada* clade**

Fig. 10A, B

Achenes without carpopodium; achene epidermis glabrous; emergences absent; subepidermal parenchyma continuous; sclerenchyma usually continuous (sometimes irregularly discontinuous), with an invagination on either side of the principal rib, parallel; air cavities absent; tannins usually absent (rarely present in cell wall only).

***Koelpinia* clade**

Fig. 10C, D

Achenes without carpopodium; achene epidermis glandular-like (elongated) papillae (almost glabrous in *K. tenuissima*); emergences present; subepidermal parenchyma continuous, of thick-walled cells only; sclerenchyma continuous, parallel (but perpendicular in emergences); air cavities absent; tannins absent.

***Takhtajaniantha* clade**

Fig. 11A–D

Achenes without carpopodium; achene epidermis with papillae or soft multicellular hairs; emergence absent or present; subepidermal parenchyma continuous; air cavities absent; tannins absent. Two types represented: (1) with parenchyma of both thin- and thick-walled cells and sclerenchyma continuous with a narrow or wider hunch on either side of the principal ribs (Takhtajaniantha type: *Scorzonera austriaca*, S. austriaca subsp. curvata, S. austriaca var. verrucosa, S. austriaca var. tenuifolia, *S. crispa*, *S. ikonnikovii*, *S. mongolica*, *S. pusilla*; Fig. 11A, B); (2) with parenchyma of thick-walled cells only and sclerenchyma either continuous or discontinuous forming 5 bundles, without hunches (Pseudodivaricata type: *S. capito*, *S. pseudodivaricata*, *S. tau-saghyz*, *S. veresczaginii*; Fig. 11C, D).

***Pseudopodospermum* clade**

Fig. 12A–F

Achenes with or without carpopodium; achene epidermis usually with papillae; emergences usually present; subepidermal parenchyma usually continuous, often reduced to one layer or few layers, of thin-walled cells only or of both thick- and thin-walled cells; air cavities absent or present in the principal ribs; sclerenchyma with parallel fibres, of three types (1) with a narrow or wider hunch on either side of the principal ribs (Brevicaulis type: *S. brevicaulis*, *S. crocifolia*, S. hispanica var. asphodeloides, *S. phaeopappa*, *S. syriaca*; Fig. 12A, B), (2) discontinuous with a gap in the principal ribs (Calyculata type: *S. calyculata*, *S. crispatula*, *S. davisii*, *S. hissarica*, *S. inaequiscapa*, *S. incisa*, *S. libanotica*, *S. ovata*, *S. papposa*, *S. reverchonii*, *S. troodea*, *S. violacea*; Fig. 12C, D) or (3) continuous and equal in thickness (Pseudopospermum type: *S. boetica*, *S. chantavica*, *S. elata*, *S. hispanica*, *S. idaea*, *S. inconspicua*, *S. gracilis*, *S. lamellata*, *S. leptophylla*, *S. mollis*, S. mollis var. platyphylla, *S. pachycephala*, *S. pubescens*, *S. raddeana*, *S. rawii*, *S. stricta*, *S. suberosa*, *S. szowitzii*, *S. turcomanica*, *S. semicana*, *S. undulata*; Fig. 12E, F.

***Scorzonera* s.str. clade**

Fig. 13A, B

Achenes without carpopodium; achene epidermis with papillae; emergences present (*S. aristata*) or absent; parenchyma insular in principal ribs below sclerenchyma and in secondary ribs above sclerenchyma, of thin-wall cells only; sclerenchyma continuous, parallel; air cavities absent; tannins absent.

***Scorzonera purpurea* clade**

Fig. 13C, D

Achenes with carpopodium; achene epidermis with papillae; emergences mostly present; parenchyma represented only by one layer or few layers above and below sclerenchyma, of both thin- and thick-walled cells; sclerenchyma continuous, parallel; air cavities absent; tannins absent.

***Scorzonera albicaulis* clade**

Fig. 14A, B

Achenes without carpopodium; achene epidermis with papillae; emergences absent; sclerenchyma with parallel fibres; air cavities usually absent; tannins absent. Two types represented: (1) subepidermal parenchyma represented by collenchymatous cells and sclerenchyma with a gap in the principal ribs (Piptopogon type: *Scorzonera angustifolia*, *S. graminifolia*, *S. baldschuanica*, *S. bracteosa*, *S. crassicaulis*, *S. petrovii*, *S. tragopogonoides*; Fig. 14A, B) and (2) subepidermal parenchyma with thin-walled cells and sclerenchyma continuous (Albicaulis type: *Scorzonera acanthoclada*, *S. albicaulis*, *S. franchetii*, *S. iliensis*, *S. racemosa*, *S. rupicola*, *S. transiliensis*, *S. turkestanica*, similar to *Scorzonera* s.str.; Fig. 13A, B).

***Podospermum* clade**

Fig. 14C, D

Achenes with carpopodium; achene epidermis glabrous or with multicellular eglandular hairs; emergences mostly absent; subepidermal parenchyma continuous of thin-walled cells or both of thin- and thick-walled cells; sclerenchyma continuous, diversely orientated (outer layers orientated parallel to the achene axis, the inner ones obliquely or perpendicularly orientated); air cavities absent; tannins absent.

The results of the ancestral character reconstruction, based on the nrITS tree, are presented in Suppl. material 1: Fig. S1. For further details, see the corresponding section in the Discussion.

## Discussion

### Phylogenetic reconstruction of the Scorzonerinae and its significance for the generic classification of the subtribe

The molecular phylogenetic study, presented here, is the one with the broadest sampling across the subtribe to date and the first comparing reconstructions, based on nuclear ribosomal and plastid DNA markers. It fully confirms previous studies (Mavrodiev et al. 2004; Winfield et al. 2006) in that *Scorzonera* is polyphyletic in all traditional circumscriptions, based on morphological data. Our broader sampling, however, elucidates an even higher extent of polyphyly than assumed so far. Actually, *Scorzonera* s.l. comprises at least six independent lineages.

Several deeper nodes of the Scorzonerinae are not resolved in either reconstruction and in previous analyses. However, our nuclear and plastid DNA trees are topologically largely congruent and supplement each other to some extent, allowing a first hypothesis on the major lineages of the Scorzonerinae and their relationships, which are discussed following the structure of the ITS tree (Fig. 1).

**The *Tourneuxia* and *Gelasia* lineages**

The nrITS tree (Fig. 1) separates three well-supported clades of the subtribe in a basal polytomy: (1) the *Tourneuxia* clade, (2) the *Gelasia* clade and (3) the remainder. In our plastid DNA tree (Fig. 2), the monospecific N African *Tourneuxia* is resolved with strong support as sister to all other members of the subtribe which are placed in a large polytomy, whereas the *Gelasia* clade is resolved with almost full support as one of the six clades of this polytomy. A sister group relationship of the monospecific N African *Tourneuxia* to all other members is the hitherto best indication for its systematic position and is not in contradiction to the nrITS tree. Taking the evidence from both trees, we may further hypothesise, thus, that *Tourneuxia* and *Gelasia* are successive sisters to the remainder of the subtribe. The nrDNA-based reconstructions by Mavrodiev et al. (2004) and Winfield et al. (2006), showed a sister group relationship of *Tourneuxia* with the *Gelasia* (as *Lasiospora*) clade or the *Gelasia* and *Tourneuxia* clades as successive sisters to the remainder of the subtribe, but without statistical support.

The N African distribution of the early diverging *Tourneuxia* lineage corroborates this region being part of the ancestral area of the tribe Cichorieae (compare Kilian et al. 2009b). This clade comprises the genus *Tourneuxia*, consisting of *T. variifolia* only, which is characterised by the annual life form and heteromorphic (entire to pinnatisect) leaves. The pollen structure of *T. variifolia*, consisting of 9 (6 abporal and 3 equatorial) lacunae, is not unique amongst Scorzonerinae and found also in *Pterachaenia* and different groups of *Scorzonera* s.l. (Blackmore 1982; Nazarova 1997). Additionally, *Tourneuxia* shares the basic chromosome number x = 7 with many other Scorzonerinae (Watanabe 2018), its characterisation by Mavrodiev et al. (2004: fig. 4) as having x = 6 being erroneous. *Tourneuxia* has a carpological type of its own with the following combination of the features: achenes somewhat flattened, with two small wing-like ribs; pappus inserted obliquely to the achene body.

The sizable ***Gelasia* lineage** has full statistical support in both our trees. Including a considerable number of species of some sections of Scorzonera subg. Scorzonera (S. sect. Anatolia, S. sect. Infrarosulares, S. sect. Nervosae, S. sect. Pulvinares, S. sect. Subaphyllae, S. sect. Trachyactis, S. sect. Tuberosae, S. sect. Vierhapperia), it is resolved far remote from the core of *Scorzonera* in both our trees. This illustrates a surprisingly strong discrepancy between the traditional classification of the genus and molecular phylogenetic results. The lineage has already been resolved by Mavrodiev et al. (2004) and Winfield et al. (2006) as the “*Lasiospora* clade”, after Scorzonera subg. Lasiospora (Cass.) Tzvelev and the genus *Lasiospora* Cass. (Cassini 1822), respectively, lectotypified by *S. hirsuta* L. (Tzvelev 1989). As we now have shown for the first time that also *Scorzonera villosa* Scop. is a member of this clade, the oldest available generic name for this lineage is *Gelasia* Cass. (Cassini 1818). *Gelasia* was separated by Cassini (1818), mostly based on the pappus structure (“aigrette irrégulière”, viz. pappus plumose in lower portion and scabrid in upper part) and *Lasiospora* was described in having pubescent achenes (Cassini 1822). As our character state reconstructions show, the heteromorphic pappus bristles are, however, a synapomorphy of the entire subtribe with only single shifts to homomorphic bristles of either type (Suppl. material 1: Fig. S1, character 15). Hairy achenes, in contrast, seem to be a synapomorphy of the *Gelasia* lineage but (a) shifts to glabrous achenes occur and (b) variously hairy achenes have been developed in three other clades of the subtribe: in the *Podospermum* clade, *Pseudopodospermum* (*S. turcomanica*, not included in the character reconstruction tree) and the *Takhtajaniantha* clade (Suppl. material 1: Fig. S1; character 3), but the pubescence of the achenes in these clades is rather scattered, indistinctly expressed or glabrescent in contrast to lanate achenes in *Gelasia*. The lineage has further carpological peculiarities (Fig. 7C, D): all species belong to the same carpological type with no carpopodium, ± continuous sclerenchyma layers with well-expressed invaginations, which in a similar way is otherwise only present in the *S. polyclada* and *Pterachaenia* clades (Suppl. material 1: Fig. S1, character 6) and, besides, with insular parenchyma above the sclerenchyma in significant thickness (Suppl. material 1: Fig. S1, character 8), similarly to that also found in other clades. The basic chromosome number for the *Gelasia* lineage seems to be x = 6 (De Santis et al. 1976; Humphries et al. 1978; Nazarova 1984; Magulaev 1986; Constantinidis et al. 2002), judging from the vast majority of chromosome number reports available (Watanabe 2018), but in a few cases x = 7 has also been reported (Sz.-Borsos 1970; Nazarova 1980; Vogt and Oberprieler 2008; Watanabe 2018). Further studies will have to show whether the deviating numbers are due to erroneous identifications or countings or whether two different basic numbers are present in this lineage, as is the case, for example, in the *Pterachaenia* lineage, see below. As no other, more distinct morphological features characterise the *Gelasia* lineage, it is no surprise that it has not been recognised as a separate genus for almost 200 years.

**The *Geropogon* and *Tragopogon* lineages**

The monospecific *Geropogon* and the sizable *Tragopogon* are consistently and with strong support resolved as sister groups in both trees. Like the previous analyses by Mavrodiev et al. (2004), Winfield et al. (2006) and Mavrodiev et al. (2012), our analysis revealed *Tragopogon* as monophyletic. The different topology by Mavrodiev et al. (2004), with *Geropogon* as sister to the *Podospermum* clade, has no statistical support and Mavrodiev et al. (2012) also revealed *Geropogon* and *Tragopogon* as sister groups with high support, based on nuclear ribosomal and nuclear markers. In the past, *Geropogon* was sometimes merged with *Tragopogon* (e.g. Richardson 1976), but more frequently accepted in its generic status, mostly based on different life form and pappus characters (annual vs. biennial or perennial and awned pappus vs. plumose pappus in outer achenes, respectively (Boissier 1875; Borisova 1964; Rechinger 1977; Meikle 1985). Pollen structure, chromosome numbers (Díaz de la Guardia and Blanca 1986, 1988; Blackmore and Barnes 1987) and anatomy of the inner achenes (Sukhorukov and Nilova 2015), moreover, distinguish both genera.

Both trees are incongruent, however, with respect to the relationships of the *Geropogon*-*Tragopogon* clade: in the nrITS tree, the *Epilasia* lineage is the sister group to the former with strong statistical support as was also found by Mavrodiev et al. (2004) and the three are sister to the other members of the subtribe, *Tourneuxia* and *Gelasia* excluded (Fig. 1). In contrast, in the plastid DNA tree, the *Scorzonera divaricata* clade is sister to the *Geropogon*-*Tragopogon* clade, while their relationship is not resolved and *Epilasia* is sister to *Podospermum* with strong support (Fig. 2).

**The *Epilasia* lineage**

This lineage is restricted to SW and W Central Asia, is in our analysis represented by two of its three annual species and received full support in the nrITS tree. *Epilasia* was first recognised as a section of *Scorzonera* by Bunge (1852) because of its annual life form, outer leaf-like phyllaries and the pappus arising from a flat or caplike pappus disk. Later, it was established at generic rank by Bentham (in Bentham and Hooker 1873) and has since been unanimously accepted as a separate genus (e.g. Lipschitz 1939; Rechinger 1977; Tagaev 1993; Mavrodiev et al. 2004; Tzvelev 2008). *Epilasia* is characterised by the unique combination of pollen structure (18 lacunae: 6 abporal, 6 paraporal, 6 polar: Blackmore 1986), the basic chromosome number x = 6 with a diploid or tetraploid chromosome complement (Watanabe 2018) and a distinct achene anatomy (see Taxonomy).

**The lineages outside the *Scorzonera-Pseudopodospermum-Takhtajaniantha* clade**

In the nrITS tree (Fig. 1) four lineages, which are also found in the plastid DNA tree, are resolved with full support, although with partly incongruent relationships.

The ***Scorzonera divaricata* lineage** so far only contains the name-giving species, a subspinescent divaricately-branched perennial herb or subshrub, restricted to N and Central China and Mongolia. In the nrITS tree, its relationship is unresolved, in the plastid DNA tree it is resolved as sister to the *Geropogon*-*Tragopogon* clade. Previously, *S. divaricata* was included in S. sect. Polyclada (Lipschitz 1939, 1964), which mostly contained the species having capitula with a few florets, a frequently homoplastic character state. Notably, the habitually most similar *S. pseudodivaricata* is found entirely unrelated, being nested in the *Takhtajaniantha* clade. The basic chromosome number of *S. divaricata* is x = 7 (Khatoon and Ali 1988). Carpologically, it is characterised by the peculiar combination of 10 ribs, subepidermal parenchyma divided into transparent cell layers and layers with tanniniferous cell walls followed by sclerenchyma equal in thickness. We recognise this lineage as a new genus *Lipschitzia* (see Taxonomy).

The ***Pterachaenia* lineage** was first established as Scorzonera sect. Pterachaenia by Bentham (in Bentham and Hooker 1873) and it was first Lipschitz (1939) who assumed that the section may deserve generic rank. *Pterachaenia* includes a single species, *P. stewartii* from E Iran, Afghanistan and Pakistan, which is peculiar by its winged achenes and annual life form. Unexpectedly, *Scorzonera codringtonii* is resolved as sister to *P. stewartii* with full support in the nrITS tree and moderate support in the plastid DNA tree. These taxa were never before considered as relatives. Both have a similar life form (annual in *P. stewartii* and annual to short-lived perennial in *S. codringtonii*) and habit, a similar achene anatomy apart from the wings in *P. stewartii* and a similar distribution area. However, following the literature, they differ in pollen structure (9 [6 abporal and 3 equatorial] lacunae in *P. stewartii* and 18 [6 abporal, 6 equatorial and 6 interporal] lacunae in *S. codringtonii* (Blackmore 1982) and chromosome numbers (*P. stewartii* having x = 6; *S. codringtonii* x = 7: Watanabe 2018).

The ***Scorzonera polyclada* lineage** is sister to *Koelpinia* in the nrITS tree, whereas the deeper nodes are unresolved (Fig. 1). In the plastid DNA tree, it is sister to the *Pterachaenia* lineage and both, in turn, are sister to *Koelpinia* (Fig. 2). In both trees, the *S. polyclada* lineage is shown here for the first time to constitute a lineage separate from the core of *Scorzonera*. The suffruticose, divaricately branching species *S. polyclada* and *S. longipapposa* of Afghanistan, as well as the more widely distributed SW Asian *S. intricata* were considered as closely related already by Rechinger (1977). Considering morphological resemblance, we assume that species, such as *S. tortuosissima* (having pollen with 24 lacunae, Askerova 1987), also belong to this lineage. Chromosome numbers x = 7, diploid (data for *S. koelpinioides* and *S. tortuosissima*: Razaq et al. 1994). We recognise this lineage as a new genus *Ramaliella* (see Taxonomy).

The ***Koelpinia* lineage** includes only the small genus *Koelpinia* (five annual species) with an Irano-Turanian distribution, extending into the S Mediterranean area. The plastid DNA tree places *Koelpinia* in closer relationship to both the *S. polyclada* and *Pterachaenia* lineages (with only moderate support) and the nrITS tree only to the former (but with strong support). A closer relationship between *Koelpinia* and *Pterachaenia* has already been revealed by Mavrodiev et al. (2004), although without statistical support. Its pollen with 15 (6 abporal, 3 equatorial, 6 interporal) lacunae is rather similar to *Tragopogon* (Blackmore 1982; Nazarova, 1997). The achenes are distinct from all other *Scorzonerinae* due to the columnar-scorpioid shape, the surface covered with retrorse subulate spine-like emergences suitable for epizoochory and peculiar, elongated, more or less stout papillae resembling glandular hairs. This lineage is another example for the co-occurrence of both basic numbers 6 and 7; moreover, it forms polyploids up to the octaploid level in *K. linearis*.

**The lineages of the *Scorzonera-Pseudopodospermum-Takhtajaniantha* clade**

Its three major clades, which include the vast majority of *Scorzonera* in the wide traditional sense, were resolved in both trees and received strong to even full support in the nrITS tree and moderate to strong support in the plastid DNA tree. The sister group relationship of *Pseudopodospermum* and *Epilasia* revealed in the plastid DNA tree but incongruent to the nrITS topology has been mentioned already above. The relationship between the three major clades is only resolved in the nrITS tree, where the *Pseudopodospermum* and *Takhtajaniantha* clades are sister (with only moderate support: PP = 0.90, BS = 67, not resolved in MP tree) and both in turn are sister to the *Scorzonera* clade with fairly strong support (PP = 0.99; BS = 98, not resolved in MP tree).

The ***Takhtajaniantha* lineage** encompasses the species of some sections of the typical subgenus of *Scorzonera*: S. sect. Fibrillosae (*Scorzonera austriaca*, *S. ikonnikovii*, *S. subacaulis*), S. sect. Egregiae (*S. tau-saghyz*), S. sect. Parviflorae p.p. (*S. mongolica*), S. sect. Papposae p.min.p. (*S. capito*), S. sect. Polyclada p.min.p. (*S. pseudodivaricata*); it also includes *S. pusilla*, the only member of S. sect. Pusillae, which was split from *Scorzonera* as the monotypic genus *Takhtajaniantha* by Nazarova (1990). Decisive for its separation was the peculiar combination of the presence of a tuber deeply sunken in the substrate, apically acuminate and somewhat coiled leaves, snow-white pappus, as well as is karyotype indicating allotetraploidy (2n = 28), there for the first time found in *Scorzonera*. The basic number of x = 7 is also shared by the other members of the lineage so far known. Later, Nazarova (1997) discovered the similarity of the pollen of *T. pusilla*, having only six abporal lacunae, to *S. austriaca*, *S. ikonnikovii* and *S. tau-saghyz*, which is a pollen type, however, also present in the *Scorzonera* s.str. clade (Blackmore 1982). *Takhtajaniantha* species possess two carpological types (“Takhtajaniantha” and “Pseudodivaricata”) (see Taxonomy and Fig. 11A–D). Many species are characterised by a caudex with fibrous leaf sheath residues, a feature, however, not exclusive to this lineage, but also present in the *S. purpurea* clade, but only here combined with lanate leaf axiles; otherwise, the lineage seems to lack morpological features distinctly circumscribing it, although several species remarkably resemble each other. The centre of the distribution of *Takhtajaniantha* is Central Asia as a part of the Irano-Turanian region, with extensions into the E Mediterranean area and E Europe (*S. pusilla*) and into Central Europe and South Siberia (*S. austriaca*).

The ***Pseudopodospermum* lineage** has full support in the nrITS tree and moderately strong support (JS = 75, PP = 0.99, BS = 73) in the plastid DNA tree. The generic rank proposed by Kuthatheladze (1978) for S. subg. Pseudopodospermum, which is typified by *Scorzonera mollis*, is corroborated by the fact that this lineage is resolved in both our reconstructions, separate from the core of *Scorzonera*. The *Pseudopodospermum* lineage, however, also includes species previously placed in sections *Incisae*, *Foliosae*, *Papposae* and Hissaricae of the former subgenus Scorzonera (Lipschitz 1964). It is predominantly distributed in the Irano-Turanian and E Mediterranean regions. Morphologically, the species united here are highly variable in many characters, such as presence of tubers and achene carpopodium, floret colour, but they are all perennial herbs. All species, so far investigated, are characterised by the basic chromosome number x = 7 and pollen with 20 (6 abporal, 6 equatorial, 6 interporal, 2 polar) lacunae (Blackmore 1986; Nazarova 1997; Pinar et al. 2016). Our carpological results show that all representatives can be divided into three carpological types that differ from each other by anatomical characters (arrangement of sclerenchyma, presence of stout conglomerations, air cavities and tanniniferous cells; Figs 3, 12A–F).

The ***Scorzonera* lineage** received strong support in both trees (nrITS: JS = 76, PP = 1, BS = 98; plastid DNA: JS = 93, PP = 1, BS = 88). All members share a basic chromosome number of x = 7. It includes four major terminal clades: the *Scorzonera* s.str. clade, the *S. purpurea* clade, the *S. albicaulis* clade and the *Podospermum* clade. Besides, *S. rupicola*, an intricately branched shrublet and *S. renzii*, a linear-leafy perennial herb with peculiar involucre, both from Iran and Turkey, respectively, form separate clades. The latter, little known species was previously placed in S. sect. Turkestanicae (Rechinger 1977; Kamelin and Tagaev 1986; Coşkunçelebı et al. 2016). The relationships between the clades of the *Scorzonera* lineage, as well as within these clades, are not well resolved. Moreover, the relationships of *S. renzii* and the *S. purpurea* clade are incongruent between the nrITS and the plastid DNA tree.

*Scorzonera* s.str. clade: This clade unites *S. humilis* (providing the type of the name *Scorzonera*), *S. aristata* and *S. parviflora*. The close relationship of *S. humilis* with *S. aristata* and *S. parviflora* was assumed already by Díaz de la Guardia and Blanca (1987), whereas it was considered as the sole member of S. sect. Parviflorae by Kamelin and Tagaev (1986) or related with *S. mongolica* by Lipschitz (1964) due to the snow-white pappus and similar tolerance of saline habitats. The following features seem characteristic for this clade: capitula solitary or by a few; apex of phyllaries often with a red spot; small, echinate or echinolophate pollen with 6 (all abporal) or 18 lacunae (*S. aristata*; Halbritter and Berger 2017; erroneously as with 15 lacunae by Díaz de la Guardia and Blanca 1985); glabrous and beakless achenes with similar anatomy (present investigation). *Scorzonera parviflora* is distributed from Central Europe to Central Asia, whereas *S. humilis* is widespread in Europe and *S. aristata* restricted to SW Europe (Kilian et al. 2009b). Another species, *S. radiata*, distributed in Central and Far East Asia (Shih and Kilian 2011), may be related to these three species, based on general morphology and carpology, but this needs confirmation by molecular phylogenetics.

*Scorzonera purpurea* clade (*S. purpurea*, *S. rosea*, *S. renzii*): *Scorzonera purpurea* and its close relative *S. rosea* were often considered to belong to S. subg. Podospermum due to the presence of a carpopodium (Mavrodiev et al. 2004; Greuter and van Raab-Straube 2006), but our analysis clearly shows the homoplasy of this character state. The main characteristics of this group are the fibrous leaf sheath residues (as otherwise present in *Takhtajaniantha*), combined with often graminoid leaves and purple or lilac florets, pollen with 18 (6 abporal, 6 equatorial, 6 interporal) lacunae, narrow achenes with the same carpological type (present investigation). Carpology corroborates the close affinity of *S. rhodantha* (SE Europe) to this clade and it has sometimes been considered as subspecies of *S. purpurea* (S. purpurea subsp. peristerica: Lack and Kilian in Strid and Tan 1991) or *S. rosea* (Greuter and van Raab-Straube 2006, sub Podospermum roseum subsp. peristericum).

*Scorzonera albicaulis* clade: This clade includes species of Scorzonera sect. Piptopogon, S. sect. Turkestanicae and S. sect. Polycladae. Morphologically, all species share the graminoid leaves, achenes attenuated into a more or less prominent beak, an easily caducous pappus and an achene surface slightly scabrid due to attenuate elongations of the epidermis cells. The presence of beaked achenes was the reason for Kamelin and Tagaev (1986) to reinstate the previously monotypic genus *Achyroseris* Sch.Bip., based on *A. macrospermum* Sch.Bip. (= *Scorzonera albicaulis* Bunge) and for the transfer of further species with beaked achenes from *Scorzonera* to *Achyroseris* by Tagaev (1993). The members of the *S. albicaulis* clade are predominantly distributed in Central Asia, extending to W Asia and the N Himalaya (*S. virgata*); one species (*S. angustifolia*) is confined to the Iberian Peninsula and Morocco. They share a pollen with 24 (6 abporal, 6 equatorial, 6 interporal, 6 polar) lacunae (Poddubnaya-Arnoldi et al. 1934, Nazarova 1997). Carpologically, the members of this clade are divided into two types. The main anatomical difference between them is the presence of remarkable subepidermal thick-walled parenchyma with well-visible intercellular spaces (Fig. 14A, B) in *S. angustifolia*, *S. baldschuanica*, *S. bracteosa* and *S. tragopogonoides*. The other members (*S. acanthoclada*, *S. albicaulis*, *S. racemosa*, *S. turkestanica* and *S. virgata*) have the same achene anatomy as the *Scorzonera* s.str. clade (Fig. 13A, B), but often with a significant reduction of the parenchyma portion and always with a yellowish, usually easily deciduous pappus (persistent and dirty white or white in the *Scorzonera* s.str. clade). Carpological and morphological characters suggest the close affinity of *S. crassicaulis*, *S. franchetii*, *S. petrovii*, *S. rupicola* (not studied by molecular phylogeny) to this clade.

*Podospermum* clade: This clade unites the majority of the members of Scorzonera subg. Podospermum sensu Kuthatheladze (1978) and the genus *Podospermum* sensu Kamelin and Tagaev (1986), respectively. Usually its members are characterised by pinnately lobed leaves, but sometimes not all leaves of an individual plant are divided and a few species (may) have only entire to sinuate leaves (*S. hieraciifolia*, *S. songorica*). Moreover, the pinnately lobed leaves are shown in our analysis to be a homoplastic state, because the pinnately leafy species of the former *Podospermum* sections *Incisae* (*S. bicolor*, *S. calyculata*, *S. incisa*, *S. libanotica*, *S. reverchonii*, *S. troodea*) and *Brevicaulis* (*S. brevicaulis*) are resolved as members of the *Pseudopodospermum* clade. All members of the *Podospermum* clade have a well-developed carpopodium, which is another homoplasy shared with the *Pseudopodospermum* clade and also with the *S. purpurea* clade. Peculiar for the members of the *Podospermum* clade are: (1) horn-like appendages on the outer phyllaries of the capitula and (2) the diversely orientated sclerenchymatous fibres in the mesocarp of the achene wall. A limited number of species, investigated palynologically, share pollen with 24 (6 abporal, 6 equatorial, 6 interporal, 6 polar) lacunae (Nazarova 1997). Most species are distributed in SW Asia, extending to W China (*S. songorica*), N Africa and S and Central Europe (*S. laciniata*).

Resolution within the clade is very poor and also, morphologically, its members are fairly uniform, indicating a young diversification age. Some samples of the widely distributed, variable species *Scorzonera laciniata* and *S. cana* occupy different positions in both the nrITS and plastid DNA trees (Figs 1, 2). This may be connected with the cryptic or poorly understood taxa described at species or varietal rank (e.g. Candolle 1838; Koch 1850, both as *Podospermum*; Chamberlain 1975; Nazarova 1995) that are identified as *S. laciniata* and *S. cana*. Besides, the distinction of both taxa is somewhat subtle, relying on the life form (monocarpic *S. laciniata* and polycarpic in *S. cana*) and the length of the ligules in relation to the involucre.

### Carpological results in the light of the phylogenetic reconstruction

According to the ancestral character reconstruction based on the nrITS tree (Fig. 1), achenes with the following features seem ancestral for the subtribe (compare Suppl. material 1: Fig. S1): achene base without carpopodium (character 1, Suppl. material 1: Fig. S1: 1) surface covered with unicellular papillae/mamillae (character 3) and without emergences (character 4, Suppl. material 1: Fig. S1: 4), having a wall with continuous sclerenchymatous layers (character 6), orientated parallel to the achene axis (character 7, Suppl. material 1: Fig. S1: 7), subepidermal continuous parenchyma layers (character 8, Suppl. material 1: Fig. S1: 8) of thin-walled cells only (character 9, Suppl. material 1: Fig. S1: 9), without air cavities (character 10, Suppl. material 1: Fig. S1: 10), with principal ribs only (character 11, Suppl. material 1: Fig. S1: 11) and without tannins (character 12, Suppl. material 1: Fig. S1: 12), achene apex without beak (character 13, Suppl. material 1: Fig. S1: 12), a pappus (character 14, Suppl. material 1: Fig. S1: 14) of entirely softly plumose bristles (with long soft fimbriae all along the bristle) (character 15, Suppl. material 1: Fig. S1: 15) of fulvous colour (character 16, Suppl. material 1: Fig. S1: 16).

*Tourneuxia* matches this ancestral type most closely, except for the presence of ribs enlarged to wings (character state 11/3, Suppl. material 1: Fig. S1: 11), a homoplastic state that occurs a second time in *Pterachaenia* and a third time in *Scorzonera armeniaca* (core *Scorzonera*-*Podospermum* clade).

Amongst the carpological characters analysed, non-homoplastic synapomorphies for clades of the Scorzonerinae are the rare exception (see below, character states 7/2, 12/1, Suppl. material 1: Fig. S1: 7, 12). Most states occur independently in several clades and, in larger clades, often more than one state is represented. These patterns may partly be explained by the fact that dispersal-related achene characters are strongly exposed and rapidly respond to selection pressure (e.g. Cody and Overton 1996; Cheptou et al. 2008) and, whenever different clades diverge under similar ecological conditions, also to parallel evolution. Achene wall anatomy seems to be less exposed to selection pressure, because the same functional result may be achieved by different anatomical constructions. Little surprisingly, amongst the least homoplastic states are anatomical ones particularly (with characters 6–10). In the following, we discuss which insights for the evolution of the carpological characters the analysis revealed.

The conspicuous tubular carpopodium (character 1, Suppl. material 1: Fig. S1: 1) in some members of the subtribe seems to have evolved at least three times, being present in the *Epilasia*, *Pseudopodospermum* and the *Scorzonera purpurea* and *Podospermum* clades. According to Haque and Godward (1984), the carpopodium as a rigid structure not contracting on drying when the achene matures, may build up tension in the cells of the abscission layer and thus facilitate abscission of the achene from the receptacle. These authors also suggested an evolution of the carpopodium in the Cichorieae from the dominating interrupted forms as basal rib outgrowths to uninterrupted ringlike forms with smooth surface, which are rare in the tribe. The aforementioned clades provide further examples of the uninterrupted form for the tribe in addition to *Lactuca* given by these authors. It seems remarkable in this context that, in the Scorzonerinae, interrupted carpopodia or a series from interrupted to more or less or fully uninterrupted ringlike, do not occur and carpopodia are either entirely ringlike or absent.

If a carpopodium is present, there is either some sort of border with respect to texture and shape between carpopodium and basal achene corpus or none (character 2, Suppl. material 1: Fig. S1: 2). It seems that the presence of such a border is plesiomorphic and, only in comparatively few topologically scattered cases, the border vanished.

The plesiomorphic state of an achene surface with unicellular papillae or mamillae (character 3, Suppl. material 1: Fig. S1: 3) seems to have shifted several times towards a glabrous surface, four times to a pubescence of multicellular eglandular hairs and one time each to drastically elongate (*Koelpinia*) and stout hair-like papillae (*Epilasia*).

Emergences of various types (character 4 and 5, Suppl. material 1: Fig. S1: 4, 5) have apparently evolved some times independently; verrucose emergences have evolved three times.

Sclerenchyma in the achene wall is responsible for the stabilty of the structure and is derived from the xylem elements of the vascular bundles of the achene wall. The ancestral condition of a continuous sclerenchymatous sheath in the wall (character 6, Suppl. material 1: Fig. S1: 6) is predominant and present in all major clades. Hunch-like outgrowths of the scleremcyma sheath flanking the principal ribs are the predominant state in the *Takhtajaniantha* clade but are also present in the *Pseudopodospermum* and *Scorzonera* s.str. clades). In other members of these two clades and in the *Gelasia* clade, the sclerenchyma shows well-expressed depressions. A differentiation of the sclerenchyma orientation with the outer layers parallel and the inner perpendicular to the achene axis seems a non-homoplastic synapomorphy of the *Podospermum* clade (character 7, Suppl. material 1: Fig. S1: 7). Perpendicular or oblique sclerenchyma layers restricted to the ribs and emergences have evolved in the *Tourneuxia*, *Epilasia* and *Koelpinia* clades.

The parenchyma of the achene wall shows considerable diversity regarding arrangement (character 8, Suppl. material 1: Fig. S1: 8) and differentiation (character 9, Suppl. material 1: Fig. S1: 9). The ancestral state of a continuous subepidermal layer (8/0) has shifted to various types of insular presence, in particular in the *Gelasia* clade. Continuous layers above and below the sclerenchyma (8/1) are present in the *Scorzonera* clade; the distribution of this state in the *Podospermum* clade for which otherwise an arrangement continuous above and insular below the sclerenchyma (8/7) was revealed as synapomorphy, indicates a close relationship between these two states. Regarding its differentiation, the parenchyma has apparently been differentiated several times in the evolution of the subtribe to support stabilisation of the achene wall, either as mechanical or collenchyma-like tissue or both (9/1–3).

Air cavities in the achene wall (character 10, Suppl. material 1: Fig. S1: 10) seem to have evolved several times independently.

Our reconstruction of evolution of the ribbing pattern (character 11, Suppl. material 1: Fig. S1: 11) revealed that, from the ancestral state with 5 prominent main ribs (11/1), shifts have occurred towards (11/2) reduction of the prominence of the ribs and a terete cross section shape, (11/3) enlargement of ribs as wings (in four terminal clades) and (11/0) differentiation of secondary ribs, whereby in the subtribe always adjacent secondary ribs are fused to a condition with 10 alternating principal and secondary ribs.

Occurrence of tannins (character 12, Suppl. material 1: Fig. S1: 12) has evolved in the cell walls (12/2) in the *Epilasia* and *S. divaricata* clades and in the protoplast (12/1) as an exclusive synapomorphy of in the *Pseudopodospermum* clade (but with reversions in several terminals).

Achene beaks (character 13, Suppl. material 1: Fig. S1: 13) have evolved several times, whereby remarkably as a synapomorphy of the *S. albicaulis* clade.

The pappus has been lost once in the *Koelpinia* clade (character 14, Suppl. material 1: Fig. S1: 14; for parallels in other subtribes, see under Taxonomic conclusions). Its structure (character 15, Suppl. material 1: Fig. S1: 15) is almost uniform in the entire subtribe: it is mostly composed of basally softly plumose and apically scabrid bristles (15/2). Shifts to purely scabrid bristles (15/3) have occurred in *Gelasia* and, in the outer achenes only (15/1) in *Geropogon*, the opposite shift to purely plumose bristles (15/0) in species of *Tragopogon*. With respect to pappus colour (character 16, Suppl. material 1: Fig. S1: 16), the fulvous colour was revealed as ancestral and as a synapomorphy of *Gelasia* (and *Tourneuxia*). Shifts are indicated to have occurred to a dirty white colour at some state of uncertain position in the evolution of the subtribe, with further shifts to yellowish, pure white or grey and reversals to fulvous.

## Taxonomic conclusions

We have shown above that morphology, even extended to include fruit anatomy, does not very well reflect the structure of the subtribe as revealed through molecular phylogenetics. Actually, most of the lineages resolved are difficult to characterise by morphology. Even more distant clades of the Scorzonerinae are often not well distinguished morphologically. Neither gross morphology nor fruit anatomy provides non-homoplastic synapomorphies for most of the major lineages. A prominent example is *Gelasia*, of which many species are nicely recognisable by the very conspicuous long-lanate achene indumentum; in a number of species, however, a reversal to glabrous achenes has occurred and what remains is some overall similarity of the *Gelasia* species which cannot be appropriately expressed in a character-state matrix or an identification key. This situation is apparently responsible for the reluctant reception of any classification of Scorzonerinae with more than the few morphologically conspicuous elements and the perseverance of a polyphyletic taxonomic concept of *Scorzonera* in spite of contrary evidence. We assume, however, that practical taxonomic experience in the application of a phylogenetic classification will bring to light new means to distinguish the various entities.

Our molecular phylogenetic analysis, based on nrITS, shows three principal options for a revised classification, based on monophyletic generic concepts. The first is to separate generically the *Gelasia* clade and retain the remainder of the current *Scorzonera*, but also include in it the genera *Pterachaenia* and *Koelpinia*. In this option *Scorzonera* would encompass clade 1C2 (Fig. 1). Since a second taxon, of *Scorzonera* and without winged achenes, was resolved in the *Pterachaenia* clade, the winged achenes are no argument against its inclusion in *Scorzonera*. The inclusion of *Koelpinia* with epizoochorous achenes without pappus seems more unconventional, but there are other cases in the Cichorieae were taxa without pappus, all traditionally placed in genera of their own for this conspicuous feature, turn out in molecular phylogenetic analyses to be nested in larger pappus-bearing genera; examples are *Lapsana* and *Rhagadiolus* being nested in *Crepis* (Enke and Gemeinholzer 2008) and *Lapsanastrum* being nested in *Youngia* (Deng et al. 2014). However, the actual inconvenience and challenge is the diagnosing of *Gelasia* as a separate genus. This burden is inevitable – unless treating the subtribe as a single genus (or as two genera, if *Tourneuxia* is retained) which is definitely undesirable – and consequently shared by all three available options. The second option is to recognise *Scorzonera* in the circumscription of clade 2 (Fig. 1). This means to separate *Gelasia*, as well as the *S. divariata* and the *S. polyclada* clades. This option retains *Koelpinia* and *Pterachaenia* as separate genera and requires thus the establishing of two other small but rather easily recognisable genera besides *Gelasia*. The third option is to recognise *Scorzonera* in the sense of clade 3 (Fig. 1), thus also generically separating *Pseudopodospermum* and *Takhtajaniantha*. This option is the one which is equally resolved in both the nrITS and plastid tree (Figs 1, 2) and the one here proposed.

### Key to the revised genera of the Scorzonerinae

The following key is a first attempt and provides some guidance rather than a truly reliable means for identification, for the reasons explained above. We expect that with the new classification at hand, taxonomic experience with the subtribe will lead to the construction of a more reliable key.

**Table:** 

1	Achenes without pappus, apically with retrorse hooked spines	*** Koelpinia ***
–	Achenes with pappus of softly plumose or scabrid bristles	**2**
2	Pappus inserted laterally at the achenes apex, situated perpendicular to the achene axis	*** Tourneuxia ***
–	Pappus inserted terminally at the achene apex	**3**
3	Involucre with outer phyllary series leaf-like and longer than the inner series; pappus greyish	*** Epilasia ***
–	Involucre with outer phyllary series shorter or equal in length to the inner series; pappus variously coloured, very rarely greyish	**4**
4	Phyllaries in one series only	**5**
–	Phyllaries in two or several series, ± imbricate	**6**
5	Perennial or biennial herbs; all achenes with pappus of plumose bristles	*** Tragopogon ***
–	Annuals; outer achenes with pappus of 5 scabrid awns, inner achenes with pappus of plumose bristles	*** Geropogon ***
6	Leaves (at least lower ones) pinnately lobed; outer phyllaries with horn-like outgrowths; achenes with carpopodium	***Scorzonera* (*Podospermum* clade)**
–	Leaves entire, rarely lobed to pinnatisect (if so, then achenes without carpopodium); phyllaries never with horn-like outgrowths	**7**
7	Caudex with fibrous and lacerate brown leaf sheath residues; florets mauve; achenes with carpopodium	***Scorzonera* (*S. purpurea* clade)**
–	Caudex without leaf sheath residues, sometimes with lacerate residues, but in the latter case, florets yellow (or yellow with purple veins) and achenes without carpopodium	**8**
8	Florets pink, yellow or orange (*S. transiliensis*); achenes almost always with beak; pappus usually easily caducous	***Scorzonera* (*S. albicaulis* clade + *S. rupicola clade* + *S. renzii* clade + *S. angustifolia* clade).**
–	Florets yellow or otherwise, but never orange; achenes without beak; pappus persistent	**9**
9	Inner phyllaries apically with dark red or blackish spot	***Scorzonera* s.str.**
–	Phyllaries apically without dark red or blackish spot	**10**
10	Annuals or perennial herbs, caudex without fibrous leaf sheath residues; florets yellow with purple veins; pappus fulvous	*** Pterachaenia ***
–	Biennials or perennial herbs or subshrubs; caudex with fibrous leaf sheath residues or not; florets entirely yellow, very rarely yellow with purple veins (*S. subacaulis*), but then with fibrous leaf sheath residues	**11**
11	Synflorescence divaricately and intricately branched	**12**
–	Synflorescence different	**13**
12	Capitula with 4–5 florets; pappus 5–8 mm, dirty-white; plants of Mongolia and N China	*** Lipschitzia ***
–	Capitula with 3–12 florets; pappus 11–18 mm, fulvous or dirty white; plants of SW Asia and NE Africa	*** Ramaliella ***
13	Achenes conspicuously and densely lanate of long soft multicellular eglandular hairs or, more rarely, glabrous and then smooth; never with carpopodium; pappus mostly fulvous and never pure white, with bristles either plumose below and scabrous above or (almost) entirely scabrous	*** Gelasia ***
–	Achenes glabrous and smooth or with verrucose ribs, rarely sparsely hairy or glabrescent; often with carpopodium; pappus of various colours but often pure white and rarely fulvous; bristles plumose below and scabrous above, never entirely scabrous	**14**
14	Caudex without leaf sheath residues; rosulate leaves basally not lanate; florets yellow, pink or purple; achenes often with carpopodium and verrucose ribs	*** Pseudopodospermum ***
–	Caudex often with basally lanate leaf sheath residues; florets yellow (sometimes with purple veins); achenes neither with carpopodium nor with verrucose ribs	*** Takhtajaniantha ***

### Revised classification of the Scorzonerinae

We provide in the following condensed profiles of the genera (and where appropriate of informal infrageneric entities) recognised, with nomenclature (typifications and synonymies), diagnostic features, brief descriptions, species lists (with new combinations, where necessary) and the distribution area (country occurrences chiefly compiled from Kilian et al. 2009b and country abbreviations following ISO 3166 ALPHA-2).

#### Tourneuxia

Taxon classificationPlantaeAsteralesAsteraceae

Coss., Bull. Soc. Bot. France 6: 395. 1859.

A66D92E0-BFBC-55C2-889F-68AF61A0C6EE

##### Type species.

*Tourneuxia variifolia* Coss.

##### Diagnostic features.

Subacaulescent annual; capitula solitary; pollen with 9 lacunae; receptacle convex; achenes winged; pappus in marginal achenes obliquely inserted.

##### Description.

***Habit, life form, subterranean parts:*** annual subaculescent herb with taproot.

***Leaves:*** ± rosulate, linear to spatulate, entire to pinnatifid.

***Stem, synflorescence:*** with one to several short weak and pubescent stems, each with solitary capitulum.

***Capitula:*** involucre tomentose, phyllaries biseriate, linear or lanceolate, equal in size; receptacle convex; capitula with > 12 florets, yellow, dorsally sometimes tinged purple, much exceeding the involucre.

***Pollen:*** echinolophate, tricolporate and each colpus divided into 2 lacunae, with 9 (6 abporal and 3 equatorial) lacunae (Blackmore 1982).

***Achenes:*** 3–6 mm, compressed, smooth, with two ribs elongated into small wings, without carpopodium; achene wall with continuous sclerenchymatous layer, of parallel orientation, sometimes the fibres of the sclerenchyma are slightly obliquely orientated in the region of the wings, parenchyma subepidermal, almost continuous, more prominent in the winged areas and much thinner (1–3 layers) between them, of thin-walled cells only, air cavities absent, tannins absent.

***Pappus:*** 4–7 mm, obliquely inserted in the marginal achenes, bristles plumose almost entirely, but scabrid in upper portion, the fimbriae of which are soft and tangled with each other, proximally brownish, distally dirty white.

##### Chromosome number.

x = 7, diploid.

##### Species.

**Figure 15.**
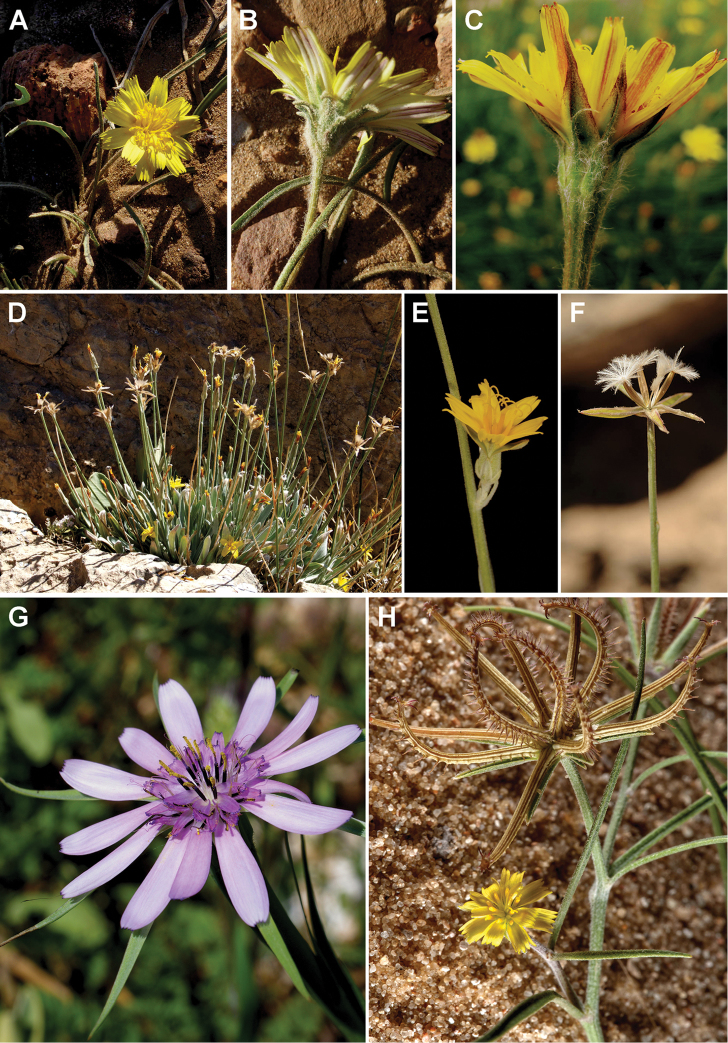
Images of living plants. **A, B** – *Tourneuxia variifolia* (Morocco, near Mrhimina). Photographs by A. Garcin (www.teline.fr) **C** – *Gelasia villosa* (cult. in BG Berlin, 13 Jun 2010). Photograph by N. Kilian **D, E, F** – *Gelasia rigida* (Jordan, Nakeb, 1250 m alt., 8 May 2019). Photograph by O. Fragman-Sapir. **D** general view **E** capitula in flowering **F** capitula in fruiting, a part of achenes had fallen off **G***Geropogon hybridus* (Greece, Lefkas, 15 May 2016). Photograph by E. Willing **H***Koelpinia linearis* (Qatar, Khashem Al Nekhsh area, 18 Mar 2016). Photograph by A. Sergeev.

(1) *T. variifolia* Coss. – Fig. 15A, B

##### Distribution area.

**N Africa** (DZ; LY; MA; TN).

#### Gelasia

Taxon classificationPlantaeAsteralesAsteraceae

Cass., Bull. Sci. Soc. Philom. Paris 1818: 33. 1818.

B646E85A-3172-530D-A1B8-E8D6DDED42F8

≡ Scorzonera sect. Gelasia (Cass.) DC., Prodr. 7(1): 123. 1838. Type: Scorzonera villosa Scop. ≡ Gelasia villosa (Scop.) Cass.
= Lasiospora Cass. in Cuvier, Dict. Sci. Nat. 25: 306. 1822.
≡ Scorzonera sect. Lasiospora (Cass.) Less., Syn. Gen. Compos.: 134. 1832.
Lasiospora ≡ Scorzonera subg. Lasiospora (Cass.) Peterm., Deutschl. Fl.: 334. 1846–1849. Lectotype (Tzvelev 1989: 45): Lasiospora hirsuta (Gouan) Cass.
= Scorzonera subsect. Pulvinares Boiss., Fl. Orient. 3: 756. 1875.
≡ Scorzonera sect. Pulvinares (Boiss.) Lipsch., Fragm. Monogr. Gen. Scorzonera 1: 24. 1935. Lectotype (designated here): Scorzonera seidlitzii Boiss.
= Scorzonera subsect. Infrarosulares Hand.-Mazz., Ann. K. K. Naturhist. Hofmus. 27: 455. 1913.
≡ Scorzonera sect. Infrarosulares (Hand.-Mazz.) Lipsch., Fragm. Monogr. Gen. Scorzonera 1: 10. 1935. Lectotype (designated here): Scorzonera acantholimon Hand.-Mazz.
= Scorzonera sect. Nervosae Lipsch., Fragm. Monogr. Gen. Scorzonera 1: 126. 1935. Type: Scorzonera latifolia (Fisch. & C.A.Mey.) DC.
= Scorzonera sect. Trachyactis Rech.f., Oesterr. Bot. Z. 84: 169. 1935. Lectotype (designated here): Scorzonera cretica Willd. Note: This section was described, based on the entirely scabrid pappus, a remarkable feature in Gelasia and the subtribe. Three species were reported to form this section: S. dependens Rech.f. (= S. cretica Willd.), S. araneosa Sm. and S. eximia Rech.f. The first species (as S. cretica) was nested in our nrITS phylogeny in the Gelasiaclade. Two species, S. cretica and S. araneosa, were included in our carpological analysis and match the carpology of Gelasia.
= Scorzonera sect. Tuberosae Lipsch., Fragm. Monogr. Gen. Scorzonera 2: 39. 1939. Type (Art. 10.8, see Turland et al. 2018): S. tuberosa Pall.
= Scorzonera sect. Vierhapperia Lipsch., Fragm. Monogr. Gen. Scorzonera 2: 88. 1939. Lectotype (designated here): Scorzonera ensifolia M.Bieb.
= Scorzonera sect. Anatolia Makbul & Coşkunç., Turk. J. Bot. 39: 77. 2015. Type: S. zorkunensis Coskunç. & Makbul

##### Diagnostic features.

Perennials; leaves entire; involucre often pubescent; achenes often densely lanate, rarely glabrous, usually smooth rarely with emergence; pappus often rigid, plumose in lower portion and scabrid in upper portion or rarely almost completely scabrid; sclerenchyma forming a sheath, with an invagination on either side of the principal rib.

##### Description.

***Habit, life form, subterranean parts:*** perennial herbs of various habits, rarely subshrubs (only *G. acantholimon*), with a taproot, more rarely with globose or cylindrical tuber.

***Leaves:*** rosulate or cauline, sessile or petiolate, linear to ovate, often hairy (frequently very densely so, fleecy, lanate) or glabrous, margin entire and flat or undulate.

***Stem, synflorescence:*** scape-like, small or leafy, tall and branched, often pubescent, more rarely glabrous (usually like those of the leaves), synflorescence racemiform, spiciform or corymbiform or capitula single.

***Capitula:*** involucre usually pubescent, sometimes glabrescent at fruiting or rarely glabrous, phyllaries in several series, all lanceolate or triangular, outer phyllaries < 3–4 times smaller than the inner ones, receptacle glabrous or hairy, capitula of many yellow florets (Fig. 15C) or, rarely, only 4–6 (*G. acantholimon*), well exceeding the involucre.

***Pollen:*** echinolophate, tricolporate and each colpus divided into 2 lacunae; with 18 (6 abporal, 6 equatorial and 6 interporal) or, more rarely, 24 (in addition with 6 polar: *G. lanata*, *G. pygmaea*) lacunae (Blackmore 1982; Pinar et al. 2016), extensive echinate polar areas present.

***Achenes:*** 4–12 mm long, without carpopodium, often densely covered with long woolly indumentum, more rarely glabrous, straight or slightly curved, 5-ribbed or roundish, smooth or rarely with stout conglomerations (*G. caespitosa*, *G. pygmaea* and *G. villosa*), parenchyma usually insular above invaginations of the sclerenchyma and below the principal ribs or only insular above invaginations of sclerenchyma, air cavities absent, sclerenchymatous layers continuous or discontinuous in the main ribs, forming a sheath, with an invagination on either side of the principal rib, fibres orientated parallel to the fruit axis, tannins absent.

***Pappus:*** 5–24 mm; bristles plumose in lower portion and scabrid in upper portion or more rarely, almost completely scabrid; dirty white, yellow or fulvous.

##### Chromosome number.

x = 6 (rarely 7), diploids or tetraploid.

##### Species.

(1) *Gelasia acantholimon* (Hand.-Mazz.) Zaika, Sukhor. & N.Kilian, comb. nov. ≡ *Scorzonera acantholimon* Hand.-Mazz. in Ann. K. K. Naturhist. Hofmus. 27: 455. 1913. urn:lsid:ipni.org:names:77204005-1

(2) *Gelasia albicans* (Coss.) Zaika, Sukhor. & N.Kilian, comb. nov. ≡ *Scorzonera albicans* Coss., Notes Crit.: 11. 1851. urn:lsid:ipni.org:names:77204006-1

(3) *Gelasia araneosa* (Sm.) Zaika, Sukhor. & N.Kilian, comb. nov. ≡ *Scorzonera araneosa* Sm. in Sibthorp, Fl. Graec. Prodr. 2(1): 123. 1813. urn:lsid:ipni.org:names:77204007-1

(4) *Gelasia aucheriana* (DC.) Zaika, Sukhor. & N.Kilian, comb. nov. ≡ *Scorzonera aucheriana* DC., Prodr. 7(1): 125. 1838. urn:lsid:ipni.org:names:77204009-1

(5) *Gelasia biebersteinii* (Lipsch.) Zaika, Sukhor. & N.Kilian, comb. nov. ≡ *Scorzonera biebersteinii* Lipsch., Fragm. Monogr. Gen. Scorzonera 2: 95. 1939. urn:lsid:ipni.org:names:77204011-1

(6) *Gelasia caespitosa* (Pomel) Zaika, Sukhor. & N.Kilian, comb. nov. ≡ *Scorzonera caespitosa* Pomel, Nouv. Mat. Fl. Atl.: 266. 1875. urn:lsid:ipni.org:names:77204012-1

(7) *Gelasia callosa* (Moris) Zaika, Sukhor. & N.Kilian, comb. nov. ≡ *Scorzonera callosa* Moris, Stirp. Sard. Elench. 1: 29. 1827. urn:lsid:ipni.org:names:77204014-1

(8) *Gelasia cinerea* (Boiss.) Zaika, Sukhor. & N.Kilian, comb. nov. ≡ *Scorzonera cinerea* Boiss., Diagn. Pl. Orient., ser. 1, 11: 44. 1849. urn:lsid:ipni.org:names:77204016-1

(9) *Gelasia circumflexa* (Krasch. & Lipsch.) Zaika, Sukhor. & N.Kilian, comb. nov. ≡ *Scorzonera circumflexa* Krasch. & Lipsch., Byull. Moskovsk. Obshch. Isp. Prir., Otd. Biol., ser. 2, 42. 148. 1934. urn:lsid:ipni.org:names:77204017-1

(10) *Gelasia cretica* (Willd.) Zaika, Sukhor. & N.Kilian, comb. nov. ≡ *Scorzonera cretica* Willd., Sp. Pl. 3: 1504. 1803. urn:lsid:ipni.org:names:77204018-1

(11) *Gelasia doriae* (Degen & Bald.) Zaika, Sukhor. & N.Kilian, comb. nov. ≡ *Scorzonera doriae* Degen & Bald., Oesterr. Bot. Z. 46: 417. 1896. urn:lsid:ipni.org:names:77204021-1

(12) *Gelasia dzhawakhetica* (Sosn. ex Grossh.) Zaika, Sukhor. & N.Kilian, comb. nov. ≡ *Scorzonera dzhawakhetica* Sosn. ex Grossh., Fl. Kavk. 4: 236. 1934. urn:lsid:ipni.org:names:77204022-1

(13) *Gelasia ensifolia* (M.Bieb.) Zaika, Sukhor. & N.Kilian, comb. nov. ≡ *Scorzonera ensifolia* M.Bieb., Fl. Taur.-Caucas. 2: 235. 1808. urn:lsid:ipni.org:names:77204023-1

(14) *Gelasia eriophora* (DC.) Zaika, Sukhor. & N.Kilian, comb. nov. ≡ *Scorzonera eriophora* DC., Prodr. 7(1): 125. 1838. urn:lsid:ipni.org:names:77204025-1

(15) *Gelasia filifolia* (Boiss.) Zaika, Sukhor. & N.Kilian, comb. nov. ≡ *Scorzonera filifolia* Boiss., Fl. Orient. 3: 774. 1875. urn:lsid:ipni.org:names:77204026-1

(16) *Gelasia hirsuta* (Gouan) Zaika, Sukhor. & N.Kilian, comb. nov. ≡ *Tragopogon hirsutus* Gouan, Fl. Monsp.: 342. 1764. urn:lsid:ipni.org:names:77204027-1

(17) *Gelasia ketzkhovelii* (Grossh.) Zaika, Sukhor. & N.Kilian, comb. nov. ≡ *Scorzonera ketzkhovelii* Sosn., Trudy Tbilissk. Bot. Inst. 2: 219. 1938. urn:lsid:ipni.org:names:77204029-1

(18) *Gelasia kotschyi* (Boiss.) Zaika, Sukhor. & N.Kilian, comb. nov. ≡ *Scorzonera kotschyi* Boiss., Fl. Orient. 3: 780. 1875. urn:lsid:ipni.org:names:77204031-1

(19) *Gelasia lanata* (L.) Zaika, Sukhor. & N.Kilian, comb. nov. ≡ *Leontodon lanatus* L., Cent. Pl. 1: 26. 1755. urn:lsid:ipni.org:names:77204032-1

(20) *Gelasia lasiocarpa* (Chamberlain) Zaika, Sukhor. & N.Kilian, comb. nov. ≡ *Scorzonera lasiocarpa* Chamberlain, Notes Roy. Bot. Gard. Edinburgh 33: 255. 1974. urn:lsid:ipni.org:names:77204034-1

(21) *Gelasia latifolia* (Fisch. & C.A.Mey.) Zaika, Sukhor. & N.Kilian, comb. nov. ≡ *Lasiospora latifolia* Fisch. & C.A.Mey., Index Sem. Hort. Petrop. 1: 30. 1835. urn:lsid:ipni.org:names:77204035-1

(22) *Gelasia litwinowii* (Krasch. & Lipsch.) Zaika, Sukhor. & N.Kilian, comb. nov. ≡ *Scorzonera litwinowii* Krasch. & Lipsch., Byull. Moskovsk. Obshch. Isp. Prir., Otd. Biol., ser. 2, 43: 153. 1934. urn:lsid:ipni.org:names:77204036-1

(23) *Gelasia longiana* (Sümbül) Zaika, Sukhor. & N.Kilian, comb. nov. ≡ *Scorzonera longiana* Sümbül, Edinburgh J. Bot. 48(1): 35. 1991. urn:lsid:ipni.org:names:77204039-1

(24) *Gelasia mackmeliana* (Boiss.) Zaika, Sukhor. & N.Kilian, comb. nov. ≡ *Scorzonera mackmeliana* Boiss., Diagn. Pl. Orient., ser. 1, 11: 44. 1849. urn:lsid:ipni.org:names:77204041-1

(25) *Gelasia mirabilis* (Lipsch.) Zaika, Sukhor. & N.Kilian, comb. nov. ≡ *Scorzonera mirabilis* Lipsch., Bot. Mater. Gerb. Bot. Inst. Komarova Akad. Nauk S.S.S.R. 22: 288. 1963. urn:lsid:ipni.org:names:77204043-1

(26) *Gelasia pisidica* (Hub.-Mor.) Zaika, Sukhor. & N.Kilian, comb. nov. ≡ *Scorzonera pisidica* Hub.-Mor., Bauhinia 7(3): 179. 1982. urn:lsid:ipni.org:names:77204044-1

(27) *Gelasia psychrophila* (Boiss. & Hausskn.) Zaika, Sukhor. & N.Kilian, comb. nov. ≡ *Scorzonera psychrophila* Boiss. & Hausskn. in Boissier, Fl. Orient. 3: 777. 1875. urn:lsid:ipni.org:names:77204045-1

(28) *Gelasia pygmaea* (Sm.) Zaika, Sukhor. & N.Kilian, comb. nov. ≡ *Scorzonera pygmaea* Sm., Fl. Graec. Prodr. 2(1): 123. 1813. urn:lsid:ipni.org:names:77204046-1

(29) *Gelasia ramosissima* (DC.) Zaika, Sukhor. & N.Kilian, comb. nov. ≡ *Scorzonera ramosissima* DC., Prodr. 7(1): 125. 1838. urn:lsid:ipni.org:names:77204047-1

(30) *Gelasia rigida* (Aucher ex DC.) Zaika, Sukhor. & N.Kilian, comb. nov. (Fig. 15D–F) ≡ *Scorzonera rigida* Aucher ex DC., Prodr. 7(1): 123. 1838. urn:lsid:ipni.org:names:77204048-1

(31) *Gelasia sandrasica* (Hartvig & Strid) Zaika, Sukhor. & N.Kilian, comb. nov. ≡ *Scorzonera sandrasica* Hartvig & Strid, Bot. Jahrb. Syst. 108(2–3): 311. 1987. urn:lsid:ipni.org:names:77204050-1

(32) *Gelasia seidlitzii* (Boiss.) Zaika, Sukhor. & N.Kilian, comb. nov. ≡ *Scorzonera seidlitzii* Boiss., Fl. Orient. 3: 775. 1875. urn:lsid:ipni.org:names:77204051-1

(33) *Gelasia sericea* (DC.) Zaika, Sukhor. & N.Kilian, comb. nov. ≡ *Scorzonera sericea* DC., Prodr. 7(1): 123. 1838. urn:lsid:ipni.org:names:77204053-1

(34) *Gelasia tomentosa* (L.) Zaika, Sukhor. & N.Kilian, comb. nov. ≡ *Scorzonera tomentosa* L., Sp. Pl., ed. 2, 2: 1112. 1763. urn:lsid:ipni.org:names:77204054-1

(35) *Gelasia tuberosa* (Pall.) Zaika, Sukhor. & N.Kilian, comb. nov. ≡ *Scorzonera tuberosa* Pall., Reise Russ. Reich. 3: 757. 1776. urn:lsid:ipni.org:names:77204057-1

(36) *Gelasia ulrichii* (Parolly & N.Kilian) Zaika, Sukhor. & N.Kilian, comb. nov. ≡ *Scorzonera ulrichii* Parolly & N.Kilian, Willdenowia 32: 198. 2002. urn:lsid:ipni.org:names:77204058-1

(37) *Gelasia villosa* (Scop.) Cass.

##### Distribution area.

**N Africa**: DZ; EG; LY; MA; TN. **Europe**: AL; BA; CY; ES; FR; GR; HR; IT; MD; ME; RS; RU; SI; UA. **Asia-Temperate**: AF; AM; AZ; CH; GE; IL; IQ; IR; JO; KG; KZ; LB; RU (Asiatic part); SY; TJ; TM; UZ. **Asia-Tropical**: PK.

The following species may also belong to *Gelasia*, based on their morphological characters (neither included in the carpological nor in molecular analyses): *Scorzonera amasiana* Hausskn. & Bornm., *S. boissieri* Lipsch., *S. bungei* Krasch. & Lipsch., *S. charadzae* Papava, *S. gageoides* Boiss., *S. ispahanica* Boiss. ≡ *Lasiospora ispahanica* (Boiss.) Walp., *S. joharchii* S.R.Safavi, *S. karabelensis* Parolly & N.Kilian, *S. kozlowskyi* Sosn. ex Grossh., *S. lipskyi* Lipsch., *S. persica* Boiss. & Buhse, *S. pulchra* Lomak., *S. safievii* Grossh., *S. sahnea* Parsa, *S. scopariiformis* Lipsch., *S. sublanata* Lipsch., *S. sericeolanata* (Bunge) Krasch. & Lipsch., *S. veratrifolia* Fenzl ≡ *Lasiospora veratrifolia* (Fenzl) Walp., *S. wendelboi* Rech.f., *S. woronowii* Krasch., *S. xylobasis* Rech.f., *S. yildirimlii* A.Duran & Hamzaoğlu, *S. zorkunensis* Coşkunç. & Makbul

#### Epilasia

Taxon classificationPlantaeAsteralesAsteraceae

(Bunge) Benth. in Bentham & Hooker, Gen. Pl. 2: 532. 1873.

0D02DDA2-9E5C-5225-ACDD-B2F928FF5430

≡ Scorzonera sect. Epilasia Bunge, Beitr. Fl. Russl.: 200. 1852. Lectotype (designated by Lipschitz (1964: 111): Epilasia hemilasia (Bunge) Kuntze

##### Diagnostic features.

Annual herbs; phyllaries in two rows, outer herbaceous, leaflike, about equalling in length the inner phyllaries; pollen with 18 lacunae; achenes with carpopodium; pappus arising from flat or caplike pappus disk, grey to blackish; bristles plumose, 5–10 of them longer, scabrid distally.

##### Description.

***Habit, life form, subterranean parts:*** annual herbs with taproot.

***Leaves:*** rosulate and lower stem leaves petiolate, upper stem leaves sessile, lanceolate to ovate, margin flat or somewhat undulate and serrulate.

***Stem, synflorescence:*** stems solitary or more frequently several (three to five), glabrous to arachnoid hairy; capitula terminal, solitary or up to eight on the stem.

***Pollen:*** echinolophate, tricolporate and each colpus divided into 2 lacunae, with 18 (6 aporal, 6 paraporal, 6 polar) lacunae (Blackmore 1982), synapomorphic pollen type.

***Capitula:*** involucre glabrous or hairy, phyllaries biseriate, outer 3–6 herbaceous, leaflike, about equal in length to the 5 inner linear-lanceolate phyllaries; receptacle flat, naked; florets more that 12, pale yellow to pale violet [or pale red or blue], more or less exceeding the involucre.

***Achenes:*** 5–10 mm, terete or with 5–10 less distinct ribs, glabrous, with carpopodium and stout hair-like papillae; either with apical flat pappus disk and pappus (*E. acrolasia*, *E. mirabilis*) or with conic caplike pappus disk and pappus covering the upper half of the achene (*E. hemilasia*); pericarp with thin-walled subepidermal parenchymatous sheath, the cell walls of which can be filled with the tannins and continuous sclerenchymatous layers orientated parallel to the fruit axis (but sometimes perpendicular in the ribs of *E. mirabilis*), air cavities absent.

***Pappus:*** 5–8 mm, dense, ash-grey or rusty, five of its bristles (rarely more) fragile, barbellate at tip, other bristles long-plumose or rarely bristles plumose in lower portion and scabrid in upper portion.

##### Chromosome number.

x = 12, amphiploids.

##### Species.

(1) *E. acrolasia* (Bunge) C.B.Clarke ex Lipsch.

(2) *E. hemilasia* (Bunge) Kuntze

(3) *E. mirabilis* Lipsch.

##### Distribution area.

**Asia-Temperate**: AF; AM; AZ; CN; GE; IQ; IR; KG; KZ; LB; SY; TJ; TM; UZ. **Asia-Tropical**: PK.

#### Tragopogon

Taxon classificationPlantaeAsteralesAsteraceae

L., Sp. Pl. 2: 789. 1753.

B5EC8B81-0647-56F5-B1FE-ADE1B093FF74

##### Lectotype.

(Díaz de la Guardia & Blanca in Jarvis et al. 1993: 95): *Tragopogon porrifolius* L.

##### Diagnostic features.

Biennial or perennial herbs with taproot; leaves mostly graminoid; involucre with phyllaries in a single series; capitula with > 20 yellow, orange, violet or purple florets; achenes often with a prominent beak; pappus plumose.

##### Description.

***Habit, life form, subterranean parts:*** Biennial or perennial herbs with a taproot.

***Leaves:*** rosulate and cauline, graminoid (sessile, subamplexicaule, with prominent midrib), linear to oblong, margin entire or undulate.

***Stem, synflorescence:*** stem solitary or branched, always leafy, glabrous to lanate; peduncle sometimes inflated.

***Capitula:*** involucre glabrous to lanate, cylindrical, phyllaries in one series, triangular or oblong, equal in size, acute, receptacle flat, naked; with > 20 florets, of various length ratios compared to involucre, yellow, orange, violet or purple.

***Pollen:*** echinolophate, tricolporate and each colpus divided into 2 lacunae, with 15 (6 abporal, 3 equatorial 6 interporal) lacunae (Blackmore 1982).

***Achenes:*** 9–55 mm, cylindrical, without carpopodium, often with a beak exceeding the length of the achene body, with 5–10 ribs, rarely some of them winglike enlarged, surface usually papillate and with stout conglomerations, rarely smooth; achene wall with parenchyma of thick-walled cells, air cavities usually present, small or large, sclerenchyma continuous, forming a sheath, cells orientated parallel to the achene axis.

***Pappus:*** 6–40 mm, bristles plumose in lower, scabrid in upper part, white, dirty white, yellowish or fulvous.

##### Chromosome number.

x = 7, diploid.

##### Species.

Appr. 150 species. The largest genus in Scorzonerinae after splitting of *Scorzonera*.

##### Distribution area.

**N Africa**: DZ; EG; LY; TN. **Europe**: all countries; **Asia-Temperate**: AF; AM; AZ; CH; GE; IL; IQ; IR; JO; KG; KW; KZ; LB; SY; SA; TJ; TK; TR; UZ. **Asia-Tropical**: IN; NP; PK.

#### Geropogon

Taxon classificationPlantaeAsteralesAsteraceae

L., Sp. Pl., ed.2, 2: 1109. 1763.

3C71AA19-9ABF-5131-BEC8-E59BC064D63B

##### Type species.

*Geropogon glaber* L. (≡ *G. hybridus* (L.) Sch.Bip.)

##### Diagnostic features.

Involucre with phyllaries in a single series; pappus of marginal achenes of five scabrid rays, pappus of inner achenes with plumose bristles.

##### Description.

***Habit, life form, subterranean parts:*** annual, ephemere.

***Leaves:*** rosulate and cauline, graminoid (sessile, subamplexicaule, with prominent midrib), linear or lanceolate, margin entire.

***Stem, synflorescence:*** stem solitary or branched, always leafy, glabrous; peduncle slightly inflated.

***Capitula:*** involucre glabrous, cylindrical, phyllaries 7–8, linear-lanceolate, in a single series, equal in size, to ~30 mm; receptacle flat, marginally hairy; florets 10–20, much exceeded by the involucre, violet or purple (Fig. 15G).

***Pollen:*** echinolophate, tricolporate and each colpus divided into 2 lacunae, with 24 (6 abporal, 6 equatorial, 6 interporal, 6 polar) lacunae (Blackmore 1982).

***Achenes:*** 32–50 mm, cylindrical, without carpopodium, with a long beak exceeding the length of the achene body, without distinct ribs, surface usually papillate and with stout conglomerations; achene wall with parenchyma of thick-walled cells, air cavities present, small, sclerenchyma continuous, forming a sheath, cells orientated parallel to the achene axis, tannins present.

***Pappus:*** with 5 glabrous awns 3–12 mm in outer achenes and plumose with dirty white bristles in inner achenes.

##### Chromosome number.

x = 6.

##### Species.

(1) *Geropogon hybridus* (L.) Sch.Bip.

##### Distribution area.

**N Africa**: EG; DZ; LY; MA; TN. **Europe**: AL; BG; CY; ES; FR; GR; HR; IT; MD; MT; PT; RU; UA. **Asia-Temperate**: AM; AZ; IQ; IL; IR; JO; LB; SY; TR.

Rare alien in DE.

#### Lipschitzia

Taxon classificationPlantaeAsteralesAsteraceae

Zaika, Sukhor. & N.Kilian gen. nov.

9DC8F22F-99A0-5BB9-82EE-E42785969877

urn:lsid:ipni.org:names:77204059-1

= Scorzonera sect. Polyclada DC., Prodr. 7(1): 125. 1838. Lectotype (designated here): Scorzonera divaricata Turcz.

##### Type species.

*Lipschitzia divaricata* (Turcz.) Zaika, Sukhor. & N.Kilian.

##### Note.

This new genus corresponds to the monotypic *Scorzonera divaricata* clade in our phylogenetic analysis. We treat the species in its narrow sense, excluding, for the time being, the varieties with more numerous florets (7–12) per capitulum, because they are likely misplaced in this species (see also Shih and Kilian 2011 sub *S. divaricata*).

##### Diagnostic features.

Subshrubs or perennial herbs; caudex with smooth scarious leaf sheath residues; stem leaves linear to filiform, only to 1 cm long, apically usually hooked; capitula numerous, with 4–5 florets.

##### Description.

***Habit, life form, subterranean parts:*** subshrubs or perennial herbs with branched caudex covered with smooth scarious leaf sheath residues.

***Leaves:*** sessile, linear to filiform, up to 1 cm, apically often hooked.

***Stem, synflorescence:*** stem divericately branched, glabrous; capitula many, terminal.

***Capitula:*** involucre puberulent, phyllaries in two series, outer phyllaries tiny, triangular or ovate, inner phyllaries oblong; receptacle naked, capitula with 4 or 5 florets; florets yellow, almost equal in length to the involucre.

***Pollen:*** data n/a.

***Achenes:*** 6–10 mm, cylindrical, with 10 ribs, smooth only apically with cylindrical papillae, without emergences and carpopodium; achene wall with both thin- and thick-walled cells (in latter case, the walls are be filled with the tannins) and continuous sclerenchymatous layers which cells orientated parallel to the achene axis, air cavities absent.

***Pappus:*** 5–8 mm, bristles plumose in lower part and scabrid in upper portion, dirty-white.

##### Chromosome number.

x = 7, diploid (Khatoon and Ali 1988 as *Scorzonera divaricata*).

##### Etymology.

The new genus is named after Sergey Yu. Lipschitz [Lipshits] (1905–1983), a Russian botanist and monographer of *Scorzonera*.

##### Species.

(1) *L. divaricata* (Turcz.) Zaika, Sukhor. & N.Kilian, comb. nov. ≡ *Scorzonera divaricata* Turcz., Bull. Soc. Imp. Naturalistes Moscou 5: 200. 1832. urn:lsid:ipni.org:names:77204060-1

##### Distribution area.

**Asia-Temperate**: CN; MN.

#### Pterachaenia

Taxon classificationPlantaeAsteralesAsteraceae

(Benth.) Stewart, Punjab Forest Rec. 2, 1 [Cat. Pl. Rawalpindi Distr.]: 50. 1952.

8BFED08C-C176-5B1A-8C15-61AA92B573DF

≡ Scorzonera sect. Pterachaenia Benth. in Bentham & Hooker, Gen. Pl. 2: 532. 1873.Type: Pterachaenia stewartii (Hook.f.) R.R.Stewart

##### Note.

Usually Lipschitz (1939: 31) is given as the place of publication for the generic name *Pterachaenia*. His statement “I think this section will also turn out to be a separate genus (gen. proprium Pterachaenia)” [translated from Russian] qualifies it, however, as a provisional genus name not formally accepted by its author.

##### Diagnostic features.

Flowering stems several or many, unbranched, leafless (scapes); phyllaries lanceolate, acute; florets yellow with red veins; achenes with 2–3 wings or without.

##### Description.

***Habit, life form, subterranean parts:*** perennial with caudex or annual herbs, with taproot.

***Leaves:*** rosulate, graminoid, linear or lanceolate, glabrous or hairy, often broadened in upper half.

***Stem, synflorescence:*** stems leafless, unbranched (scapes) several to many, pubescent in lower part and glabrous in upper part, with terminal capitulum.

***Pollen:*** echinolophate, tricolporate and each colpus divided into 2 lacunae, with 9 (6 abporal and 3 equatorial: *P. stewartii*) or 18 (6 abporal, 6 equatorial, 6 interporal: *P. codringtonii*) lacunae (Blackmore 1982).

***Capitula:*** involucre usually pubescent, phyllaries in two to several series, linear or lanceolate, acute, outer phyllaries 1/4–1/2 the length of the inner ones; receptacle glabrous, florets 12–22, yellow with red veins, equal in length to inner phyllaries.

***Achenes:*** 12–15 mm, straight, without carpopodium; with 2–3 elongated ribs forming wings denticulate in upper part (*Pterachaenia stewartii*) or without wings (*P. codringtonii*), papillate and with small emergences (spinulae) (*P. stewartii*) or with smooth surface (*P. codringtonii*), with five principal ribs, parenchyma of two types, with thick-walled cells and with thin-walled cells, discontinuous (located above sclerenchyma between the principal ribs and below sclerenchyma in the rib areas) or only insular above sclerenchyma, air cavities absent, sclerenchyma continuous, equal in thickness, fibres orientated parallel to the fruit axis, tannins absent.

***Pappus:*** 13–20 mm, fulvous, bristles plumose, apically scabrid.

##### Chromosome number.

x = 6 (*Pterachaenia stewartii*) and x = 7 (*P. codringtonii*), diploids.

##### Distribution area.

**Asia-Temperate**: AF; IR. **Asia-Tropical**: PK.

##### Species.

(1) *Pterachaenia codringtonii* (Rech.f.) Zaika, Sukhor. & N.Kilian, comb. nov. ≡ *Scorzonera codringtonii* Rech.f., Oesterr. Bot. Z. 97: 263. 1950. urn:lsid:ipni.org:names:77204061-1

(2) *Pterachaenia stewartii* (Hook.f.) R.R.Stewart in Punjab Forest Rec. 2, 1: 50. 1952.

#### Koelpinia

Taxon classificationPlantaeAsteralesAsteraceae

Pall., Reise Russ. Reich. 3: 755. 1776.

19EBB35B-CA75-5833-9C97-C8192AFB5695

##### Type species.

*Koelpinia linearis* Pall.

##### Diagnostic features.

Annual herbs; pollen with 15 lacunae; at least outer achenes columnar-scorpioid, surface with hooked emergences and elongated papillae looking like glandular hairs, (sometimes achenes smooth, without emergences and papillae: *K. tenuissima*), without pappus, but apically with hooked, retrorse spines.

##### Description.

***Habit, life form, subterranean parts:*** annual herbs with a taproot.

***Leaves:*** subsessile, filiform to narrowly oblong, entire.

***Stem, synflorescence:*** stem solitary or mostly branched, glabrous or with scattered simple hairs; synflorescence mostly branched, with several capitula.

***Capitula:*** involucre glabrous to hairy, phyllaries in several series, narrowly oblong to lanceolate, outer phyllaries much shorter than inner; receptacle naked or with scattered setae; capitula with 5–12 yellow florets, much exceeding the involucre.

***Pollen:*** echinolophate, tricolporate and each colpus divided into 2 lacunae, with 15 (6 abporal, 3 equatorial, 6 interporal) lacunae, often pollen tetracolporate in polyploids (Blackmore 1981).

***Achenes:*** 6–25 mm, usually columnar-scorpioid, (sub)annular (*K. macrantha*) or almost straight (*K. tenuissima* and *K. turanica*), with 5 or 10 more or less prominent ribs, surface often covered with emergences resembling hooked spines and glandular-like (elongated) papillae or surface almost glabrous in *K. tenuissima*, without carpopodium; achene wall with or without air cavities, parenchyma continuous above sclerenchyma represented by thick-walled cells, sclerenchyma continuous, its fibres orientated parallel to the fruit axis (perpendicular in emergences), tannins absent.

***Pappus:*** bristles absent, achenes with several retrorse hooked spines (Fig. 15H).

##### Chromosome number.

x = 6 for *K. macrantha* (Podlech and Bader 1974; Nazarova 1981) or 7 (all others species) (Stebbins et al. 1953; Podlech and Bader 1974; Nazarova 1981), diploids to octaploids.

##### Species.

(1) *K. chrysoglochis* Rech.f.

(2) *K. linearis* Pall.

(3) *K. macrantha* C.Winkl.

(4) *K. tenuissima* Pavlov & Lipsch.

(5) *K. turanica* Vassilcz.

##### Distribution area.

**N Africa**: DZ; EG; LY; TN. **Asia-Temperate**: AF; AM; AZ; BH; CN; CY; GE; IL; IQ; IR; KG; KW; KZ; LB; QA; SA; SY; TJ; TM; TR; UZ. **Asia-Tropical**: IN; PK. **Europe**: ES; RU; UA.

#### Ramaliella

Taxon classificationPlantaeAsteralesAsteraceae

Zaika, Sukhor. & N.Kilian gen. nov.

008D20C2-FEB1-53F1-B00E-9969CEB94DD5

urn:lsid:ipni.org:names:77204063-1

= Scorzonera subsect. Intricatae Boiss., Fl. Orient. 3: 756. 1875.
≡ Scorzonera sect. Intricatae (Boiss.) Lipsch., Fragm. Monogr. Gen. Scorzonera 1: 10. 1935. Type (Art. 10.8, see Turland et al. 2018): Scorzonera intricata Boiss.

##### Type species.

*Ramaliella polyclada* (Rech.f. & Köie) Zaika, Sukhor. & N.Kilian

##### Note.

The new genus corresponds to the *Scorzonera polyclada* clade. Morphologically, *Ramaliella* resembles *Lipschitzia divaricata*, but differs by the caudex surface, size of the capitula and the achene anatomy of a type of its own.

##### Diagnostic features.

**Figure 16.**
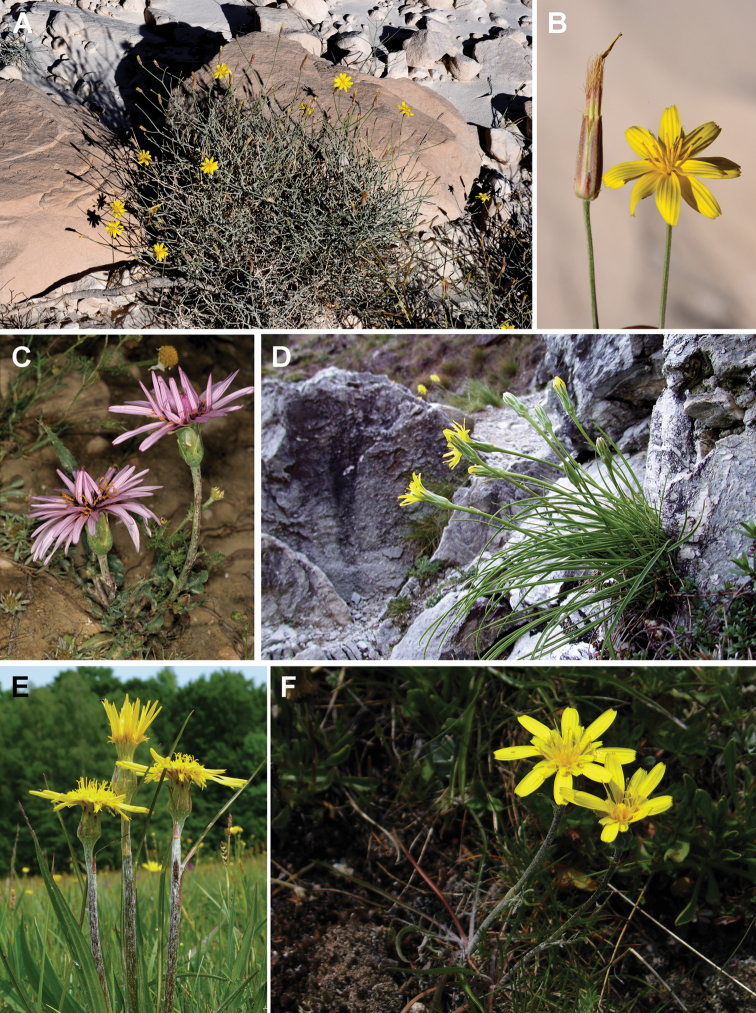
Images of living plants. **A, B***Ramaliella tortuosissima***A** general view **B** close up view of the capitula (Jordan, Nakeb, 1250 m alt., 8 May 2019). Photograph by O. Fragman-Sapir **C***Pseudopodospermum papposum* (Jordan, Nakeb, 8 May 2019). Photograph by O. Fragman-Sapir **D***Takhtajaniantha austriaca* (Italy, South Tyrol, Schlanders, 6 May 2010). Photograph by R. Hand **E***Scorzonera humilis* (Germany, Ammersee, 15 May 2009). Photograph by C. Niederbichler **F***Scorzonera meyeri* (Russia, Karachaevo-Cherkessiya, Teberda, 14 July 2005). Photograph by A.S. Zernov.

Subshrubs or perennial herbs; stems numerous, intricately and divaricately branched (Fig. 16A); leaves few, the lower leaves filiform, the upper leaves reduced, curved; pollen with 24 lacunae; capitula terminal, with 3–12 florets.

##### Description.

***Habit, life form, subterranean parts:*** subshrubs or perennial herbs with taproot, often with cushion-like habit.

***Leaves:*** few, the lower leaves filiform, the upper ones reduced, curved, entire or denticulate.

***Synflorescence:*** stems numerous, strongly divaricately and intricately branched, glabrous or puberulent, capitula terminal.

***Capitula:*** involucre glabrous or pubescent, phyllaries in several series, the outer phyllaries tiny, triangular, the inner phyllaries linear-lanceolate much longer than the outer ones, receptacle naked, flat, florets 3–12, yellow, slightly exceeding the involucre (Fig. 16B).

***Pollen:*** echinolophate, tricolporate and each colpus divided into 2 lacunae, with 24 (6 abporal, 6 equatorial, 6 interporal, 6 polar) lacunae (*R. tortuosissima*: Askerova 1987).

***Achenes:*** 7–12 mm, cylindrical, without carpopodium, with five main ribs and five (or even more) barely noticeable secondary ribs, surface smooth; achene wall with parenchyma of both thin- and thick-walled cells, sclerenchyma forming a sheath (sometimes irregularly discontinuous), with an invagination on either side of the principal rib, cells orientated parallel to the achene axis, air cavities absent, tannins absent or rarely present in cell wall only (*R. koelpinioides*).

***Pappus:*** 11–18 mm, fulvous or dirty white, bristles plumose, but in upper part scabrid.

##### Chromosome number.

x = 7, diploid (data for *R. koelpinioides* and *R. tortuosissima*: Razaq et al. 1994).

##### Etymology.

The new genus is named after the specific “brushwood” habit (rāmālia: brushwood).

##### Species.

(1) *Ramaliella intricata* (Boiss.) Zaika, Sukhor. & N.Kilian, comb. nov. ≡ *Scorzonera intricata* Boiss., Diagn. Pl. Orient., ser. 1, 7: 9. 1846. urn:lsid:ipni.org:names:77204064-1

(2) *Ramaliella koelpinioides* (Rech.f.) Zaika, Sukhor. & N.Kilian, comb. nov. ≡ *Scorzonera koelpinioides* Rech.f., Fl. Iranica 122: 53. 1977. urn:lsid:ipni.org:names:77204065-1

(3) *Ramaliella longipapposa* (Rech.f.) Zaika, Sukhor. & N.Kilian, comb. nov. ≡ *Scorzonera longipapposa* Rech.f., Fl. Iranica 122: 53. 1977. urn:lsid:ipni.org:names:77204066-1

(4) *Ramaliella musilii* (Velen.) Zaika, Sukhor. & N.Kilian, comb. nov. ≡ *Scorzonera musilii* Velen., Sitzungsber. Königl. Böhm. Ges. 9: 8. 1911. urn:lsid:ipni.org:names:77204068-1

(5) *Ramaliella polyclada* (Rech.f. & Köie) Zaika, Sukhor. & N.Kilian, comb. nov. ≡ *Scorzonera polyclada* Rech.f. & Köie, Biol. Skr. 8(2): 195. 1955. urn:lsid:ipni.org:names:77204069-1

(6) *Ramaliella tortuosissima* (Boiss.) Zaika, Sukhor. & N.Kilian, comb. nov. ≡ *Scorzonera tortuosissima* Boiss., Fl. Orient. 3: 775. 1875. urn:lsid:ipni.org:names:77204071-1

##### Distribution area.

**N Africa**: EG. **Asia-Temperate**: AE; AF; IQ; IR; JO; KU; SA; YE. **Asia-Tropical**: PK.

The following species may belong to *Ramaliella*, based on their morphological characters (they were neither included in the carpological nor in molecular investigations): *Scorzonera hondae* Kitam., *S. microcalathia* (Rech.f.) Rech.f., *S. subaphylla* Boiss., *S. yemensis* Podlech

#### Pseudopodospermum

Taxon classificationPlantaeAsteralesAsteraceae

(Lipsch. & Krasch.) Kuth., Kavkaz. Predstav. Scorzonerinae: 85. 1978.

976E0259-90D6-5E0E-9120-F3BC0CD42C3F

≡ Scorzonera sect. Pseudopodospermum Lipsch. & Krasch. in Lipschitz, Fragm. Monogr. Gen. Scorzonera 1: 70. 1935.
≡ Scorzonera subg. Pseudopodospermum (Lipsch. & Krasch.) Lipsch. in Bobrov & Tzvelev, Fl. URSS 29: 48. 1964. Lectotype (designated by Lipschitz 1964: 49): Scorzonera mollis M.Bieb.
= Scorzonera subsect. Foliosae Boiss., Fl. Orient. 3: 756. 1875.
≡ Scorzonera sect. Foliosae (Boiss.) Lipsch. in Bobrov & Tzvelev, Fl. URSS 29: 721. 1964. Lectotype (designated by Lipschitz 1964: 721): S. stricta Hornem.
= Scorzonera sect. Incisae Lipsch., Fragm. Monogr. Gen. Scorzonera 1: 91. 1935.
≡ Podospermum sect. Incisae (Lipsch.) Kuth., Kavkaz. Predstav. Scorzonerinae: 99. 1978. Type (Art. 10.8, see Turland et al. 2018): Scorzonera incisa DC.
= Scorzonera sect. Papposae Lipsch. & Krasch. in Lipsch., Fragm. Monogr. Gen. Scorzonera 1: 45. 1935. Type (Art. 10.8, see Turland et al. 2018): Scorzonera papposa DC.
= Scorzonera ser. Brevicaulis Lipsch., Fragm. Monogr. Gen. Scorzonera 1: 92. 1935. Type (Art. 10.8, see Turland et al. 2018): Scorzonera brevicaulis Vahl
= Scorzonera subsect. Hissarica Lipsch., Fragm. Monogr. Gen. Scorzonera 2: 63. 1939.
≡ Scorzonera sect. Hissarica Lipsch. in Bobrov & Tzvelev, Fl. URSS 29: 97. 1964. Type (Art. 10.8, see Turland et al. 2018): Scorzonera hissarica C.Winkl.

##### Diagnostic features.

Perennial herbs often with a tuber; leaves entire, often with undulate margin or pinnatifid; capitula solitary or few; pollen with 20 lacunae; achenes usually papillate, with stout conglomerations or verrucose ribs.

##### Description.

***Habit, life form, subterranean parts:*** perennial herbs mostly with a tuber, or else with taproot.

***Leaves:*** rosulate only or cauline, lower leaves usually petiolate, upper leaves sessile and often (sub) amplexicaule, their shape diverse (linear-lanceolate to broadly ovate), margin entire and then often undulate, or dentate to pinnatisect.

***Stem, synflorescence:*** stem solitary or with a few branches, sometimes leafless and scapose, glabrous to lanate; capitula terminal.

***Capitula:*** involucre glabrous to lanate, phyllaries in several series, outer phyllaries triangular or ovate, obtuse, inner phyllaries oblong or lanceolate, much longer than outer phyllaries, receptacle naked, flat, florets more than 12, exceeding the involucre up to 1.5 times, yellow or purple (Fig. 16C), sometimes bicoloured, with yellow ligulae and dark purple mouth.

***Pollen:*** echinolophate, tricolporate and each colpus divided into 2 lacunae, with 20 (6 abporal, 6 equatorial, 6 interporal, 2 polar) lacunae (Blackmore 1982; Pinar et al. 2016).

***Achenes:*** 8–28 mm, straight or slightly curved, with or without carpopodium, usually with five, rarely with ten ribs or without, surface papillate and/or usually with stout conglomerations or the outer strongly rugose or tuberculate-squamate; achene wall with parenchyma subepidermal, continuous (or very rarely sometimes irregularly discontinuous), often reduced to one- to several layers consisting of thin- and/or thick-walled cells, sometimes with subepidermal mechanical parenchyma, air cavities absent or present, sclerenchyma discontinuous with a gap in the principal ribs or continuous forming a sheath, equal in thickness or with a narrow or wider hunch on either side of the principal rib, sclerenchymatous fibres orientated parallel to the achene axis, tannins absent or present in the cell protoplasts.

***Pappus:*** 6–28 mm, bristles equal or unequal (5–10 bristles often longer than the rest), plumose in lower and scabrid in upper part, dirty white or white, yellowish, blackish or rarely fulvous.

##### Chromosome number.

x = 7, diploids, tetraploids or hexaploids.

##### Species.

(1) *Pseudopodospermum boeticum* (Boiss.) Zaika, Sukhor. & N.Kilian, comb. nov. ≡ Scorzonera montana var. boetica Boiss. in Candolle, Prodr. 7(1): 121. 1838 ≡ *Scorzonera boetica* (Boiss.) Boiss., Voy. Bot. Espagne 2: 382. 1841. urn:lsid:ipni.org:names:77204074-1

(2) *Pseudopodospermum brevicaule* (Vahl) Zaika, Sukhor. & N.Kilian, comb. nov. ≡ *Scorzonera brevicaulis* Vahl, Symb. Bot. 2: 88. 1791. urn:lsid:ipni.org:names:77204076-1

(3) *Pseudopodospermum calyculatum* (Boiss.) Zaika, Sukhor. & N.Kilian, comb. nov. ≡ *Scorzonera calyculata* Boiss., Diagn. Pl. Orient., ser. 1, 11: 42. 1849. urn:lsid:ipni.org:names:77204078-1

(4) *Pseudopodospermum chantavicum* (Pavlov) Zaika, Sukhor. & N.Kilian, comb. nov. ≡ *Scorzonera chantavica* Pavlov, Vestn. Akad. Nauk Kazakhsk. S.S.R. 8: 27. 1950. urn:lsid:ipni.org:names:77204079-1

(5) *Pseudopodospermum crispatulum* (DC.) Zaika, Sukhor. & N.Kilian, comb. nov. ≡ Scorzonera hispanica var. crispatula DC., Prodr. 7(1): 121. 1838 ≡ *Scorzonera crispatula* (DC.) Boiss., Voy. Bot. Espagne 2: 741. 1845. urn:lsid:ipni.org:names:77204082-1

(6) *Pseudopodospermum crocifolium* (Sm.) Zaika, Sukhor. & N.Kilian, comb. nov. ≡ *Scorzonera crocifolia* Sm. in Sibthorp, Fl. Graec. Prodr. 2(1): 123. 1813. urn:lsid:ipni.org:names:77204084-1

(7) *Pseudopodospermum davisii* (Lipsch.) Zaika, Sukhor. & N.Kilian, comb. nov. ≡ *Scorzonera davisii* Lipsch., Bot. Mater. Bot. Inst. Komarova Akad. Nauk S.S.S.R. 22: 291. 1963. urn:lsid:ipni.org:names:77204085-1

(8) *Pseudopodospermum elatum* (Boiss.) Zaika, Sukhor. & N.Kilian, comb. nov. ≡ *Scorzonera elata* Boiss., Diagn. Pl. Orient., ser. 1, 4: 25. 1844. urn:lsid:ipni.org:names:77204086-1

(9) *Pseudopodospermum gracile* (Lipsch.) Zaika, Sukhor. & N.Kilian, comb. nov. ≡ *Scorzonera gracilis* Lipsch. in Bobrov & Tzvelev, Fl. URSS 29: 721. 1964. urn:lsid:ipni.org:names:77204088-1

(10) *Pseudopodospermum hispanicum* (L.) Zaika, Sukhor. & N.Kilian, comb. nov. ≡ *Scorzonera hispanica* L., Sp. Pl. 2: 791. 1753. urn:lsid:ipni.org:names:77204090-1

(11) *Pseudopodospermum hissaricum* (C.Winkl.) Zaika, Sukhor. & N.Kilian, comb. nov. ≡ *Scorzonera hissarica* C.Winkl., Trudy Imp. S.-Peterburgsk. Bot. Sada 11: 172. 1889. urn:lsid:ipni.org:names:77204092-1

(12) *Pseudopodospermum idaeum* (Gand.) Zaika, Sukhor. & N.Kilian, comb. nov. ≡ *Podospermum idaeum* Gand., Bull. Soc. Bot. France 62: 155. 1916. urn:lsid:ipni.org:names:77204093-1

(13) *Pseudopodospermum inaequiscapum* (Boiss.) Zaika, Sukhor. & N.Kilian, comb. nov. ≡ *Scorzonera inaequiscapa* Boiss., Fl. Orient. 3: 762. 1875. urn:lsid:ipni.org:names:77204091-1

(14) *Pseudopodospermum incisum* (DC.) Zaika, Sukhor. & N.Kilian, comb. nov. ≡ *Scorzonera incisa* DC., Prodr. 7(1): 119. 1838. urn:lsid:ipni.org:names:77204089-1

(15) *Pseudopodospermum inconspicuum* (Lipsch.) Zaika, Sukhor. & N.Kilian, comb. nov. ≡ *Scorzonera inconspicua* Lipsch., Byull. Moskovsk. Obshch. Isp. Prir., Otd. Biol., n.s. 42: 128. 1933. urn:lsid:ipni.org:names:77204087-1

(16) *Pseudopodospermum lamellatum* (Krasch.) Zaika, Sukhor. & N.Kilian, comb. nov. ≡ *Scorzonera lamellata* Krasch., Trudy Bot. Inst. Akad. Nauk S.S.S.R., ser. 1, Fl. Sist. Vyssh. Rast. 1: 180. 1933. urn:lsid:ipni.org:names:77204083-1

(17) *Pseudopodospermum leptophyllum* (DC.) Kuth.

(18) *Pseudopodospermum libanoticum* (Boiss.) Zaika, Sukhor. & N.Kilian, comb. nov. ≡ *Scorzonera libanotica* Boiss., Diagn. Pl. Orient., ser. 1, 11: 43. 1849. urn:lsid:ipni.org:names:77204081-1

(19) *Pseudopodospermum molle* (M.Bieb.) Kuth.

(20) *Pseudopodospermum mucidum* (Rech.f., Aellen & Esfand.) Zaika, Sukhor. & N.Kilian, comb. nov. ≡ *Scorzonera mucida* Rech.f., Aellen & Esfand., Oesterr. Bot. Z. 97: 264. 1950. urn:lsid:ipni.org:names:77204077-1

(21) *Pseudopodospermum ovatum* (Trautv.) Zaika, Sukhor. & N.Kilian, comb. nov. ≡ *Scorzonera ovata* Trautv., Trudy Imp. S.-Peterburgsk. Bot. Sada 1: 275. 1871. urn:lsid:ipni.org:names:77204075-1

(22) *Pseudopodospermum pachycephalum* (Podlech & Rech.f.) Zaika, Sukhor. & N.Kilian, comb. nov. ≡ *Scorzonera pachycephala* Podlech & Rech.f. in Rechinger, Fl. Iranica 122: 36. 1977. urn:lsid:ipni.org:names:77204073-1

(23) *Pseudopodospermum papposum* (DC.) Zaika, Sukhor. & N.Kilian, comb. nov. ≡ *Scorzonera papposa* DC., Prodr. 7(1): 119. 1838. urn:lsid:ipni.org:names:77204072-1

(24) *Pseudopodospermum phaeopappum* (Boiss.) Zaika, Sukhor. & N.Kilian, comb. nov. ≡ *Podospermum phaeopappum* Boiss., Diagn. Pl. Orient., ser. 1, 7: 5. 1846. urn:lsid:ipni.org:names:77204070-1

(25) *Pseudopodospermum pubescens* (DC.) Zaika, Sukhor. & N.Kilian, comb. nov. ≡ *Scorzonera pubescens* DC., Prodr. 7(1): 122. 1838. urn:lsid:ipni.org:names:77204067-1

(26) *Pseudopodospermum raddeanum* (C.Winkl.) Zaika, Sukhor. & N.Kilian, comb. nov. ≡ *Scorzonera raddeana* C.Winkl., Trudy Imp. S.-Peterburgsk. Bot. Sada 11: 150. 1889. urn:lsid:ipni.org:names:77204062-1

(27) *Pseudopodospermum reverchonii* (O.Debeaux ex Hervier) Zaika, Sukhor. & N.Kilian, comb. nov. ≡ *Scorzonera reverchonii* O.Debeaux ex Hervier, Bull. Acad. Int. Géogr. Bot., sér. 3, 15(187–188): 107. 1905. urn:lsid:ipni.org:names:77204056-1

(28) *Pseudopodospermum semicanum* (DC.) Zaika, Sukhor. & N.Kilian, comb. nov. ≡ *Scorzonera semicana* DC., Prodr. 7(1): 119. 1838. urn:lsid:ipni.org:names:77204055-1

(29) *Pseudopodospermum strictum* (Hornem.) Zaika, Sukhor. & N.Kilian, comb. nov. ≡ *Scorzonera stricta* Hornem., Hort. Bot. Hafn.: 2: 750. 1815. urn:lsid:ipni.org:names:77204052-1

(30) *Pseudopodospermum suberosum* (K.Koch) Kuth., Kavkaz. Predstav. Scorzonerinae: 94. 1978

(31) *Pseudopodospermum syriacum* (Boiss. & C.I.Blanche) Zaika, Sukhor. & N.Kilian, comb. nov. ≡ *Scorzonera syriaca* Boiss. & C.I.Blanche, Diagn. Pl. Orient., ser. 2, 3: 93. 1856. urn:lsid:ipni.org:names:77204049-1

(32) *Pseudopodospermum szowitzii* (DC.) Kuth., Kavkaz. Predstav. Scorzonerinae: 92. 1978.

(33) *Pseudopodospermum troodeum* (Boiss.) Zaika, Sukhor. & N.Kilian, comb. nov. ≡ *Scorzonera troodea* Boiss., Fl. Orient., suppl.: 320. 1888. urn:lsid:ipni.org:names:77204042-1

(34) *Pseudopodospermum turcomanicum* (Krasch. & Lipsch.) Zaika, Sukhor. & N.Kilian, comb. nov. ≡ *Scorzonera turcomanica* Krasch. & Lipsch. in Lipschitz, Fragm. Monogr. Gen. Scorzonera 1: 80. 1935. urn:lsid:ipni.org:names:77204040-1

(35) *Pseudopodospermum undulatum* (Vahl) Zaika, Sukhor. & N.Kilian, comb. nov. ≡ *Scorzonera undulata* Vahl, Symb. Bot. 2: 86. 1791. urn:lsid:ipni.org:names:77204038-1

(36) *Pseudopodospermum violaceum* (Chamberlain) Zaika, Sukhor. & N.Kilian, comb. nov. ≡ *Scorzonera violacea* Chamberlain, Notes Roy. Bot. Gard. Edinburgh 33(2): 256. 1974. urn:lsid:ipni.org:names:77204037-1

##### Distribution area.

**N Africa**: DZ; LY; MA; TN. **Europe**: AL; AT; BA; BE; BG; CH; CY; CZ; DE; ES; FR; GR; HR; HU; IT; MD; ME; MK; PL; PT; RO; RS; RU; SK; SI; UA. **Asia-Temperate**: AF; AM; AZ; EG; GE; IL; IQ; IR; JO; KW; KZ; LB; OM; SY; SA; TJ; TK; TR; UZ. **Asia-Tropical**: PK. *Pseudopodospermum hispanicum* as an edible crop was introduced in further regions.

The following species may belong to *Pseudopodospermum*, based on their morphological characters (they were included neither in the carpological nor in molecular investigations): *Scorzonera acuminata* Boiss., *S. aragatzii* Kuth. ≡ *Pseudopodospermum aragatzii* (Kuth.) Kuth., *S. coriacea* A.Duran & Aksoy, *S. crassifolia* Krasch. & Lipsch., *S. drarii* Täckh., *S. ferganica* Krasch., *S. gorovanica* Nazarova, *S. helodes* Rech.f., *S. karkasensis* S.R.Safavi, *S. lacera* Boiss. & Balansa, *S. limnophila* Boiss., *S. mariovoensis* Micevski, *S. mucida* Rech.f. et al., *S. pacis* Güzel et al., *S. nivalis* Boiss. & Hausskn., *S. rawii* Rech.f. & Guest, *S. scyria* M.A.Gust. & Snogerup, *S. serpentinica* Rech.f., *S. stenocephala* Boiss., *S. tadshikorum* Krasch. & Lipsch., *S. tunicata* Rech.f. & Köie, *S. tuzgoluensis* A.Duran et al.

#### Takhtajaniantha

Taxon classificationPlantaeAsteralesAsteraceae

Nazarova, Biol. Zhurn. Armenii 43: 179. 1990.

7ADBE6F6-5D69-5409-BC6E-9468436F2166

= Scorzonera sect. Fibrillosae Nakai, Rep. Inst. Sci. Res. Manchoukuo, ser. 1, 6: 171. 1937. Type: Scorzonera glabra Rupr. [= S. austriaca Willd.]
= Scorzonera subsect. Egregia Kult., Tau-Sagyz Ekol. Osnovy Vvedeniya Ego Kul’t.: 108. 1938.
≡ Scorzonera subg. Egregia (Kult.) Ovcz., Soobshch. Tadzhiksk. Fil. Akad. Nauk SSSR 20: 54. 1950.
≡ Scorzonera sect. Egregia (Kult.) Lipsch., Fl. URSS 29: 58. 1964. Lectotype (designated here): Scorzonera tau-saghyz Lipsch. & G.G.Bosse
= Scorzonera sect. Pusillae Lipsch., Fragm. Monogr. Gen. Scorzonera 2: 59. 1939. Type: Scorzonera pusilla Pall.
= Scorzonera sect. Capito Tzvelev, Rast. Tsentral. Azii 14b: 115. 2009. Type: Scorzonera capito Maxim.

##### Type species.

*Takhtajaniantha pusilla* (Pall.) Nazarova

##### Diagnostic features.

Perennials; caudex often with dark brown fibrous leaf sheath residues and base of the rosulate leaves often lanate; pollen with 6 lacunae.

##### Description.

***Habit, life form, subterranean parts:*** perennial herbs or subshrublets with a taproot or a tuber.

***Leaves:*** rosulate or crowded in the basal part of the stem, rarely dispersed along the stem, sessile or petiolate, filiform or ovate, apically sometimes hooked (*T. pusilla*), margin flat or undulate, entire or denticulate.

***Stem, synflorescence:*** stem solitary or branched, scape-like or leafy, capitula terminal.

***Pollen:*** echinolophate, tricolporate and each colpus divided into 2 lacunae, with 6 abporal lacunae (sometimes with spineless areas resembling rudimentary lacunae (Blackmore 1982).

***Capitula:*** glabrous to arachnoid hairy or tomentose, phyllaries in several series, outer phyllaries smaller than inner ones, triangular-ovate, inner phyllaries linear to ovate, receptacle glabrous, flat, with many or rarely few (up to 12: *T. pseudodivaricata*) florets; florets yellow (Fig. 16D) or with dark red veins, usually clearly exeeding the incolucre.

***Achenes:*** 5–15 mm, without carpopodium, straight or slightly curved, often with 10 ribs or rarely roundish, glabrous or hairy, sometimes (as in *T. pusilla*) with stout conglomerations; achene wall with parenchyma of two types, with thick- and thin-walled cells, present only as subepidermal continuous layer(s) or only with thick-walled cells, air cavities absent, sclerenchyma with continuous layer with a narrow or wider hunch on either side of the principal rib or, as in *T. pseudodivaricata*, without hunches, but discontinuous forming 5 bundles (with a gap in the principal ribs), or continuous, its fibres orientated parallel to the fruit axis, tannins absent.

***Pappus:*** 7–28 mm, ice white or dirty white, bristles scabrid in upper part.

***Chromosome number:*** x = 7 (diploids); 2n = 28 in *T. pusilla* (amphiploid); in *T. tau-saghyz* polyploid cytoraces 2n = 14, 21, 28, 42 and aneuploids with chromosome numbers 15, 16, 17, 18, 22 and 24, 28 and 42 are known (Krayevoy 1934; Poddubnaya-Arnoldi et al. 1934; Sosnovets 1960).

**Species.**

(1) *Takhtajaniantha austriaca* (Willd.) Zaika, Sukhor. & N.Kilian, comb. nov. (Fig. 16D) ≡ *Scorzonera austriaca* Willd., Sp. Pl. 3(3): 1498. 1803 (incl. *S. glabra* Rupr.). urn:lsid:ipni.org:names:77204033-1

(2) *Takhtajaniantha capito* (Maxim.) Zaika, Sukhor. & N.Kilian, comb. nov. ≡ *Scorzonera capito* Maxim., Bull. Acad. Imp. Sci. Saint-Pétersb. 32: 491. 1888. urn:lsid:ipni.org:names:77204030-1

(3) *Takhtajaniantha crispa* (M.Bieb.) Zaika, Sukhor. & N.Kilian, comb. nov. ≡ *Scorzonera crispa* M.Bieb., Fl. Taur.-Caucas. 2: 234. 1808. urn:lsid:ipni.org:names:77204028-1

(4) *Takhtajaniantha ikonnikovii* (Krasch. & Lipsch.) Zaika, Sukhor. & N.Kilian, comb. nov. ≡ *Scorzonera ikonnikovii* Krasch. & Lipsch. in Lipschitz, Fragm. Monogr. Gen. Scorzonera 1: 109. 1935. urn:lsid:ipni.org:names:77204024-1

(5) *Takhtajaniantha mongolica* (Maxim.) Zaika, Sukhor. & N.Kilian, comb. nov. ≡ *Scorzonera mongolica* Maxim., Bull. Acad. Imp. Sci. Saint-Pétersb. 32: 492. 1888. urn:lsid:ipni.org:names:77204020-1

(6) *Takhtajaniantha pseudodivaricata* (Lipsch.) Zaika, Sukhor. & N.Kilian, comb. nov. ≡ *Scorzonera pseudodivaricata* Lipsch., Byull. Moskovsk. Obshch. Isp. Prir., Otd. Biol. 42: 158. 1933. urn:lsid:ipni.org:names:77204019-1

(7) *Takhtajaniantha pusilla* (Pall.) Nazarova

(8) *Takhtajaniantha subacaulis* (Regel) Zaika, Sukhor. & N.Kilian, comb. nov. ≡ Scorzonera austriaca var. subacaulis Regel, Trudy Imp. S.-Peterburgsk. Bot. Sada 6: 323. 1880. urn:lsid:ipni.org:names:77204015-1

(9) *Takhtajaniantha tau-saghyz* (Lipsch. & G.G.Bosse) Zaika, Sukhor. & N.Kilian, comb. nov. ≡ *Scorzonera tau-saghyz* Lipsch. & G.G.Bosse, Trudy Nauchno-Issl. Lab. Tresta "Kauchukonos" 4: 27. 1930. urn:lsid:ipni.org:names:77204010-1

(10) *Takhtajaniantha veresczaginii* (Kamelin & S.V.Smirn.) Zaika, Sukhor. & N.Kilian, comb. nov. ≡ *Scorzonera veresczaginii* Kamelin & S.V.Smirn., Turczaninowia 5(1): 17. 2002. urn:lsid:ipni.org:names:77204008-1

##### Distribution area.

**N Africa**: EG. **Asia-Temperate**: AF; AR; AZ; CN; IL; IR; JO; KG; KP; KR; KZ; MN; RU (Asiatic part); SA; TJ; TM; UZ. **Asia-Tropical**: PK. **Europe**: AL; AT; BA; BG; BY; CH; CZ; DE; FR; GR; HR; HU; IT; MD; ME; RO; RS; RU; SI; SK; UA.

The following species may belong to *Takhtajaniantha*, based on their morphological characters (they were included neither in the carpological nor in molecular investigations):

*Scorzonera aniana* N.Kilian, *S. grubovii* Lipsch., *S. karataviensis* Kult. (possibly a synonym of *T. tau-saghyz*), *S. manshurica* Nakai, *S. pamirica* C.Shih, *S. sinensis* (Lipsch. & Krasch.) Nakai

#### Scorzonera

Taxon classificationPlantaeAsteralesAsteraceae

L., Sp. Pl.: 790. 1753.

209CDCFE-7223-51AF-9029-5928108FB2B4

##### Lectotype.

(Green in Hitchcock and Green 1929: 177): *Scorzonera humilis* L.

##### Note.

*Scorzonera* in this revised sense as a monophyletic genus includes four larger clades, the *Scorzonera* s.str. clade (3 spp.), *Scorzonera albicaulis* clade (~17 spp.), *Podospermum* clade (~20 spp.) and *Scorzonera purpurea* clade (2 spp.). In addition, it includes a few, so far somewhat isolated species (*S. rupicola*, *S. renzii*, *S. angustifolia*). The genus in this cricumscription still shows considerable variation. Our analysis is insufficient with respect to a revision of the elaborated infrageneric classifications of previous authors. We therefore refer here to the clades of our trees.

#### Scorzonera

Taxon classificationPlantaeAsteralesAsteraceae

s.str. clade

CC342938-05E6-5C98-8DBC-E81888283F93

Scorzonera sect. Parviflorae Lipsch. in Bobrov & Tzvelev, Fl. URSS 29: 79. 1964 ≡ Scorzonera ser. Parviflorae Lipsch. in Bobrov & Tzvelev, Fl. URSS 29: 720. 1964 [S. subsect. Parviflora Lipsch., Fragm. Monogr. Gen. Scorzonera 2: 112. 1939, nom. inval. (Art. 39.1, Turland et al. 2018)]. Type: Scorzonera parviflora Jacq.
= Scorzonera sect. Radiatae Nakai in Rep. Inst. Sci. Res. Manchoukuo, ser. 1, 6: 169. 1937. Type: Scorzonera radiata Fisch. ex Ledeb.

##### Diagnostic features.

Inner phyllaries apically often with dark red or blackish spot; achene sclerenchyma insular in principal ribs below sclerenchyma and in secondary ribs above sclerenchyma (in *S. aristata*, the parenchyma is present in secondary ribs above sclerenchyma).

##### Description.

***Habit, life form, subterranean parts:*** perennial or biennial herbs, often with leaf rosettes, rootstock cylindrical.

***Leaves:*** rosulate leaves present, petiolate, cauline leaves sessile, all leaves entire, linear to elliptical or ovate, glabrous or glabrescent at maturity.

***Stem, synflorescence:*** stems solitary or few, straight, mostly not branched, leafless (scapes) or leafy, glabrous or arachnoid-hairy becoming almost glabrous at the fruiting; capitula terminal, solitary or several.

***Pollen:*** echinolophate, tricolporate and each colpus divided into 2 lacunae; with 6 abporal lacunae (*S. humilis* and *S. parviflora*: Blackmore 1982; Díaz de la Guardia and Blanca 1985) or 18 (6 abporal, 6 equatorial and 6 interporal) lacunae (*S. aristata*: Halbritter and Berger 2017).

***Capitula:*** glabrous or hairy and glabrescent, phyllaries in several series, outer phyllaries triangular-ovate, inner phyllaries lanceolate to oblong, apically with dark red or blackish spot, outer phyllaries at least ½ as long as inner ones, receptacle glabrous, flat, florets more than 12, yellow or whitish, equal in length to involucre or 1½–2 times as long.

***Achenes:*** 7–12 mm, without carpopodium, glabrous, achene epidermis with papillae, outer achenes more or less curved, with 10 ribs, without emergences or verrucose (*S. aristata*); achene wall with parenchyma insular in principal ribs below sclerenchyma and in secondary ribs above sclerenchyma, sclerenchyma continuous, its fibres orientated parallel to the achene axis, air cavities and tannins absent.

***Pappus:*** 11–16 mm, dirty white or rarely ice-white (*S. parviflora*), bristles plumose or apically scabrid (or 5–10 longer bristles scabrid and other bristles plumose).

##### Chromosome number.

x = 7, diploids.

##### Species.

(1) *Scorzonera aristata* Ramond ex DC.

(2) *Scorzonera humilis* L. (Fig. 16E)

(3) *Scorzonera parviflora* Jacq.

##### Distribution area.

**Asia-Temperate**: AF; AM; AR; CN; CY; GE; IR; KG; LB; MN; RU (Asiatic part); SY; TM; TR; UZ. **Europe**: AT; BE; BY; CH; CZ; DE; DK; EE; ES; FI; FR; GB; HR; IT; LT; LV; NL; NO; PL; PT; RO; RS; RU; SE; SK; SI; UA.

*Scorzonera radiata* Fisch. ex Ledeb. may also belong to this clade on account of its resemblance in morphological and carpological characters.

#### Podospermum

Taxon classificationPlantaeAsteralesAsteraceae

clade

5F63FF32-413D-5312-9D16-1811DF1FCFA5

≡ Podospermum DC. in Lamarck & Candolle, Fl. Franç., ed. 3, 4: 61. 1805, nom. cons.
≡ Scorzonera sect. Podospermum (DC.) Benth. in Bentham & Hooker, Gen. Pl. 2: 532. 1873.
≡ Scorzonera subg. Podospermum (DC.) Lipsch., Fragm. Monogr. Gen. Scorzonera 1: 7. 1935. Type: Podospermum laciniatum (L.) DC.

##### Diagnostic features.

Herbs, biennial or perennial; leaves (at least some) pinnately divided; phyllaries in several series, often subapically corniculate; achene with conspicuous cylindrical carpopodium usually 1/5–1/3 as long as achene body; achene surface mostly smooth, more rarely verrucose or undulate and glabrous or rarely somewhat hairy; pollen with 18 or 24 lacunae; sclerenchyma in achene mesophyll of diverse orientation (outer layers orientated parallel to the achene axis, the inner ones obliquely or perpendicularly orientated).

##### Description.

***Habit, life form, subterranean parts:*** biennial or perennial herbs, with taproot, often with woody caudex.

***Leaves:*** glabrous or pubescent, mostly located in the lower third of the stem, rosulate leaves usually present. Heterophylly often present (basal leaves petiolate, pinnatifid or pinnatisect, cauline leaves (sub)sessile, lobate to entire).

***Stem, synflorescence:*** stem solitary or branched, leafy (especially in lower third), glabrous or pubescent, capitula terminal.

***Pollen:*** echinolophate, tricolporate and each colpus divided into 2 lacunae; with 24 (6 abporal, 6 equatorial, 6 interporal and 6 polar: Pinar et al. 2016) or, more rarely, 18 (6 abporal, 6 equatorial, 6 interporal; *S. laciniata*: Blackmore 1982).

***Capitula:*** involucre pubescent and glabrescent at fruiting, phyllaries in several series, outer phyllaries triangular or ovate, inner phyllaries narrower, oblong or lanceolate, 2–3 times longer than outer phyllaries, receptacle naked, florets yellow, > 20, equal to or longer than involucre.

***Achenes:*** 6–13 mm, with carpopodium, outer achenes slightly curved, with 10, rarely 5 ribs or roundish (two achene ribs *S. armeniaca* and *S. alpigena* can be elongated into small wings), glabrous or with scattered pubescence; achene wall with parenchyma continuous, above sclerenchyma and also well-expressed (continuous or discontinuous) in the principal ribs below sclerenchyma), sclerenchyma continuous, diversely orientated (outer layers orientated parallel to the achene axis, the inner ones obliquely or perpendicularly orientated), air cavities and tannins absent.

***Pappus:*** 5–14 mm, white or dirty white, bristles plumose and only apically scabrid.

##### Chromosome number.

x = 7, diploids.

##### Species.

(1) *Scorzonera alpigena* (K.Koch) Grossh.

(2) *Scorzonera armeniaca* (Boiss. & A.Huet) Boiss.

(3) *Scorzonera cana* (C.A.Mey.) Griseb.

(4) *Scorzonera grossheimii* Lipsch. & Vassilcz.

(5) *Scorzonera hieraciifolia* Hayek

(6) *Scorzonera kandavanica* Rech.f.

(7) *Scorzonera kirpicznikovii* Lipsch.

(8) *Scorzonera lachnostegia* (Woronow) Lipsch.

(9) *Scorzonera laciniata* L.

(10) *Scorzonera luristanica* Rech.f.

(11) *Scorzonera meshhedensis* (Rech.f.) Rech.f.

(12) *Scorzonera meyeri* (K.Koch) Lipsch. (Fig. 16F)

(13) *Scorzonera persepolitana* Boiss.

(14) *Scorzonera radicosa* Boiss.

(15) *Scorzonera schischkinii* Lipsch. & Vassilcz.

(16) *Scorzonera songorica* (Kar. & Kir.) Lipsch. & Vassilcz.

##### Distribution area.

**Africa**: DZ; EG; ES (Canary Islands); LY; MA; TN. **Asia-Temperate**: AF; AM; AZ; CN; CY; GE; GR; IL; IQ; IR; JO; KG; KZ; LB; RU (Asiatic part); SY; TJ; TR; TM; UZ. **Asia-Tropical**: IN; PK. **Europe**: all countries.

*Scorzonera laciniata* is introduced in **Australasia** (AU); **North America** (US); **South America** (AR).

The following species may also belong to the *Podospermum* clade based on their morphological characters: *Scorzonera grigoraschvilii* (Sosn.) Lipsch., *S. idae* (Sosn.) Lipsch., *S. lipschitzii* (Kuth.) Czerep.

#### Scorzonera albicaulis

Taxon classificationPlantaeAsteralesAsteraceae

clade

850D3E8E-61F8-5682-AD30-BA07D25B7EBE

= Achyroseris Sch.Bip., Nov. Actorum Acad. Caes. Leop.-Carol. Nat. Cur. 21: 165. 1845. Type: Achyroseris macrosperma (Turcz. ex DC.) Sch.Bip. (= S. albicaulis Bunge). Note: Schultz (1845) based his segregation of Achyroseris from Scorzonera on the “paleate receptacle” in A. macrosperma. This is, however, clearly erroneous, because a paleate (bracteate) receptacle is not present in any member of the Scorzonerinae; at most, the receptacle can be slightly hairy in some members of the phylogenetically distant Gelasia.
= Scorzonera sect. Piptopogon C.A.Mey., Bull. Soc. Imp. Naturalistes Moscou 21(3): 97. 1848.
≡ Scorzonera subg. Piptopogon (C.A.Mey.) C.Díaz & Blanca, Anales Jard. Bot. Madrid 43: 330. 1987. Type: Scorzonera macrosperma Turcz. ex DC. (= S. albicaulis Bunge)
= Scorzonera sect. Turkestanicae Lipsch. in Bobrov & Tzvelev, Fl. URSS 29: 720. 1964. Type: Scorzonera turkestanica Franch.

##### Diagnostic features.

Perennial herbs and subshrubs; pollen with 24 lacunae; achenes beaked; pappus dirty yellow, caducous.

##### Description.

***Habit, life form, subterranean parts:*** perennial herbs and subshrubs with a taproot and often with caudex.

***Leaves:*** rosulate and cauline, numerous, usually sessile (rosulate leaves petiolate in *S. franchetii*), semi-amplexicaule, linear to narrowly oblong, more often lanceolate, entire or crisp, glabrous or slightly pubescent.

***Stem, synflorescence:*** stem solitary or several, usually leafy but bracteate in *S. acanthoclada* and *S. racemosa*, capitula terminal and solitary or numerous, in spiciform or corymbiform synflorescence.

***Pollen:*** echinolophate, tricolporate and each colpus divided into 2 lacunae; with 24 (6 abporal, 6 equatorial, 6 interporal, 6 polar) lacunae (Poddubnaya-Arnoldi et al. 1934; Nazarova 1997).

***Capitula:*** pubescent and often glabrescent, phyllaries in several series, outer phyllaries 1/2–1/3 as long as inner ones, triangular-ovate, the inner phyllaries triangular to lanceolate, receptacle glabrous, capitula with 4–12 florets, yellow, pink or orange (*S. transiliensis*), 1.5–2 times exceeding the involucre.

***Achenes:*** 7–45 mm, straight, without carpopodium, with more or less expressed beak, 10- or rarely 5-ribbed, papillate; achene wall with parenchyma well-expressed and represented by collenchyma-like cells, then present only as subepidermal continuous layers or sometimes parenchyma absent or discontinuous in the rib areas, insular in principal ribs below sclerenchyma and in secondary ribs above sclerenchyma or absent, air cavities absent, sclerenchyma present as layers with a gap in the principal ribs or continuous sclerenchymatous layers, its fibres orientated parallel to the fruit axis, tannins absent.

***Pappus:*** 6–28 mm, yellowish, bristles plumose below and scabrid in upper part.

##### Chromosome number.

x = 7, diploids.

##### Species.

(1) *Scorzonera acanthoclada* Franch.

(2) *Scorzonera albicaulis* Bunge

(3) *Scorzonera baldschuanica* Lipsch.

(4) *Scorzonera bracteosa* C.Winkl.

(5) *Scorzonera crassicaulis* Rech.f.

(6) *Scorzonera franchetii* Lipsch.

(7) *Scorzonera graminifolia* L.

(8) *Scorzonera petrovii* Lipsch.

(9) *Scorzonera racemosa* Franch.

(10) *Scorzonera tragopogonoides* Regel & Schmalh.

(11) *Scorzonera transiliensis* Popov

(12) *Scorzonera turkestanica* Franch.

(13) *Scorzonera virgata* DC.

##### Distribution area.

**Africa**: MA. **Asia-Temperate**: AF; RU (Asian part); CN; IR; KG; KP; KR; KZ; MN; TM; TJ; UZ. **Asia-Tropical**: IN; PK. **Europe**: ES; PT.

*Scorzonera alaica* Lipsch. may also belong to this clade based on morphological features.

#### Scorzonera purpurea

Taxon classificationPlantaeAsteralesAsteraceae

clade

A3291AE4-CE61-5239-8369-AED7B5136CA9

Scorzonera sect. Purpurea Lipsch., Fragm. Monogr. Gen. Scorzonera 2: 104. 1939. Type (Art. 10.8, see Turland et al. 2018): Scorzonera purpurea L.

##### Diagnostic features.

Perennial herbs; caudex with blackish-brown fibrous leaf sheath residues; leaves entire; florets purple or pink; achene with carpopodium.

##### Description.

***Habit, life form, subterranean parts:*** perennial herbs with caudex covered with fibrous leaf sheath residues and taproot.

***Leaves:*** leaves linear or lanceolate, sessile, glabrous or basally pubescent, rosulate leaves often as long as stem; cauline leaves much shorter.

***Stem, synflorescence:*** stems usually solitary or rarely several stems present, mostly not branched, glabrous or scarcely pubescent, capitula solitary.

***Pollen:*** echinolophate, tricolporate and each colpus divided into 2 lacunae; with 18 (6 abporal, 6 equatorial, 6 interporal) lacunae (Blackmore 1982).

***Capitula:*** involucre almost glabrous or slightly pubescent mainly in lower part, receptacle glabrous, flat, involucre in several series, outer phyllaries triangular, inner phyllaries 2–3 times longer than outer ones, lanceolate, florets more than 12, exceeding the involucre up to two times, pink or purple.

***Achenes:*** 6–15 mm, with carpopodium, straight, with 5 (sometimes slightly verrucose) ribs or flattened-triangular, glabrous or papillate: achene wall with parenchyma scarcely represented by one or several layers above and below sclerenchyma, of two types (mechanical parenchyma and such with thin-walled), air cavities absent, sclerenchyma continuous, of equal thickness throughout, sclerenchymatous fibres orientated parallel to the achene axis, tannins absent.

***Pappus:*** 7–13 mm, dirty white, bristles plumose in lower part and apically scabrid, 5–10 of them clearly longer than other bristles.

##### Chromosome number.

x = 7, diploids.

##### Species.

(1) *Scorzonera purpurea* L.

(2) *Scorzonera rosea* Waldst. & Kit.

(3) *Scorzonera rhodantha* Hausskn.

##### Distribution area.

**Asia-Temperate**: KZ; RU (South Siberia). **Asia-Tropical**: IN; PK. **Europe**: AL; AT; BA; BG; CZ; DE; FR; GR; HR; HU; IT; MD; ME; MK; PL; RO; RS; RU; SI; SK; UA.

### Species of unclear position

*Scorzonera renzii* (not carpologically studied) is sister to the *Scorzonera purpurea* clade in our nrITS tree and forms a polytomy with the *Scorzonera purpurea* and *Podospermum* clades in the plastid DNA tree. The spiciform synflorescences and other morphological features (tall stem, graminoid rosulate leaves without residues; pollen with 24 lacunae) rather indicate an affinity to the *S. albicaulis* clade.

*Scorzonera angustifolia* (pollen with 24 lacunae) and *S. rupicola* show some affinity to the *S. albicaulis* clade, but the second is resolved in the nrITS tree as sister to the polytomy including the *S. purpurea* and *S. albicaulis* clades, whereas the first is the third element of that polytomy.

### Excluded names

Scorzonera sect. Pentachlamys DC., Prodr. 7: 125. 1838, including two species from Nepal, *S. bupleuroides* D.Don, Prodr. Fl. Nepal.: 162. 1825 and *S. roylei* DC., Prodr. 7: 125. 1838, do not refer to *Scorzonera* or one of the genera segregated here. The holotype of *S. roylei* in G-DC (G00498633) is a species of *Tragopogon*. Original material of *S. bupleuroides* seems to be lost and the species is hardly a member of *Scorzonera* or one of its segregates as was already concluded by Lipschitz (1939).

## Supplementary Material

XML Treatment for Tourneuxia

XML Treatment for Gelasia

XML Treatment for Epilasia

XML Treatment for Tragopogon

XML Treatment for Geropogon

XML Treatment for Lipschitzia

XML Treatment for Pterachaenia

XML Treatment for Koelpinia

XML Treatment for Ramaliella

XML Treatment for Pseudopodospermum

XML Treatment for Takhtajaniantha

XML Treatment for Scorzonera

XML Treatment for Scorzonera

XML Treatment for Podospermum

XML Treatment for Scorzonera albicaulis

XML Treatment for Scorzonera purpurea

## Supplementary materials

Supplementary material 1 Figure S1. Evolution of carpological characters in the Scorzonerinae reconstructed with parsimony with Mesquite version 3.5 using the nrDNA tree Data type: morphological data Explanation note: For character-state coding, see Results; the matrix is given in Table 2. https://binary.pensoft.net/file/370365This dataset is made available under the Open Database License (http://opendatacommons.org/licenses/odbl/1.0/). The Open Database License (ODbL) is a license agreement intended to allow users to freely share, modify, and use this Dataset while maintaining this same freedom for others, provided that the original source and author(s) are credited.Maxim A. Zaika, Norbert Kilian, Katy Jones, Anastasiya A. Krinitsina, Maya V. Nilova, Anna S. Speranskaya, Alexander P. Sukhorukov
