# Supplementary figures and images for: Scorzonera sensu lato (Asteraceae, Cichorieae) – taxonomic reassessment in the light of new molecular phylogenetic and carpological analyses
Source: PhytoKeys. 2020 Jan 8;137:1–85. doi: 10.3897/phytokeys.137.46544 (PMC6962254; doi:10.3897/phytokeys.137.46544)

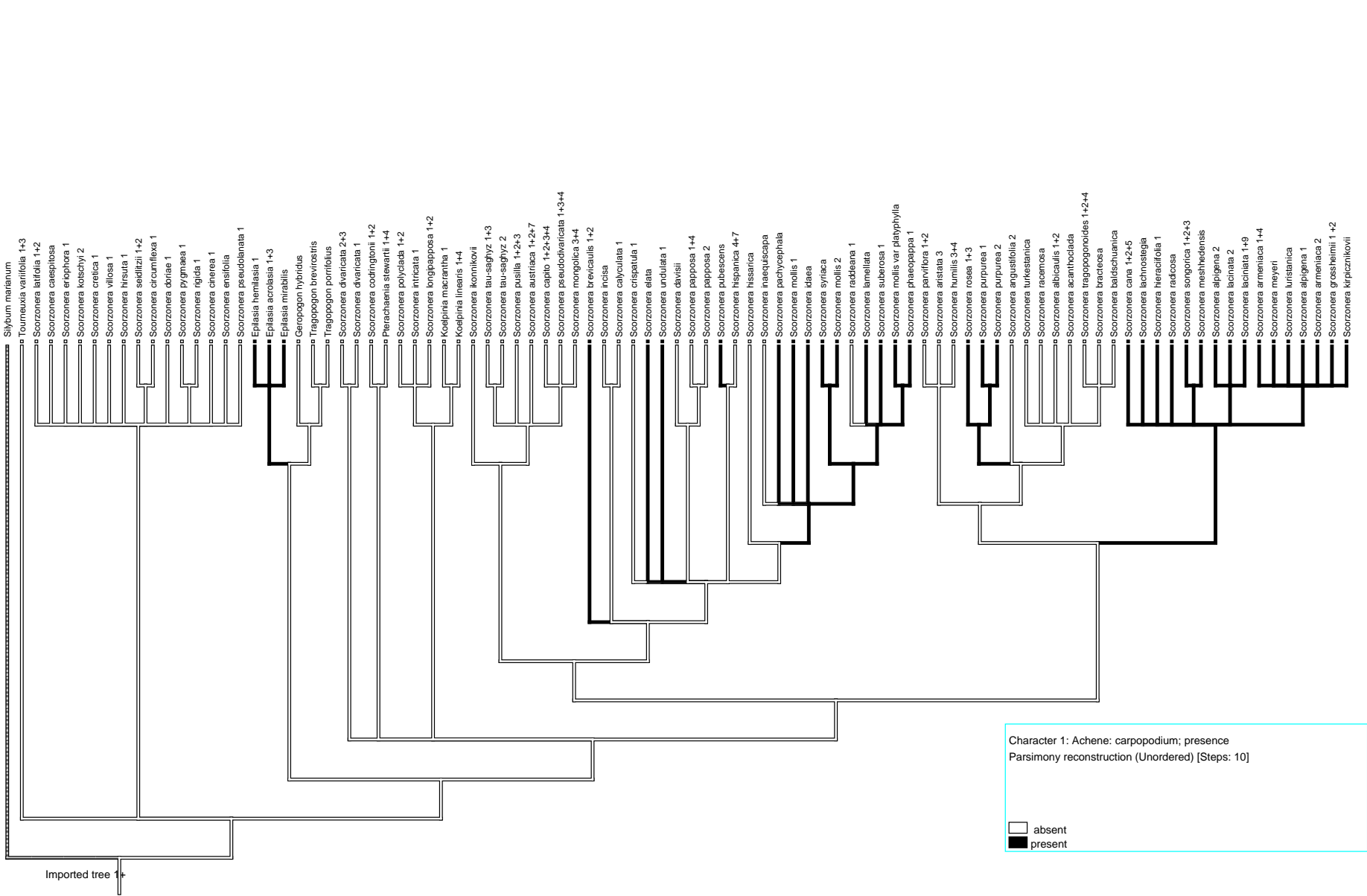

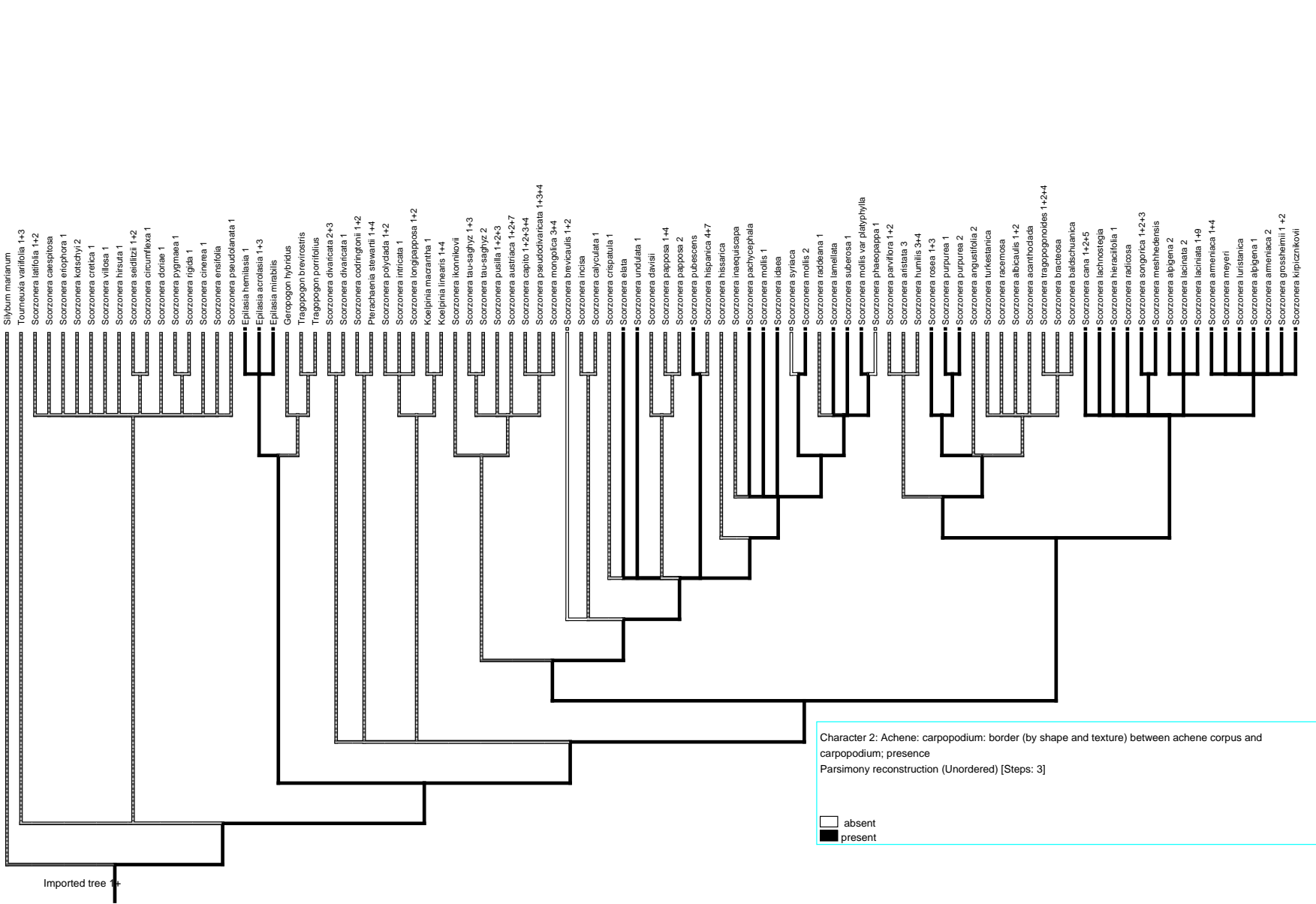

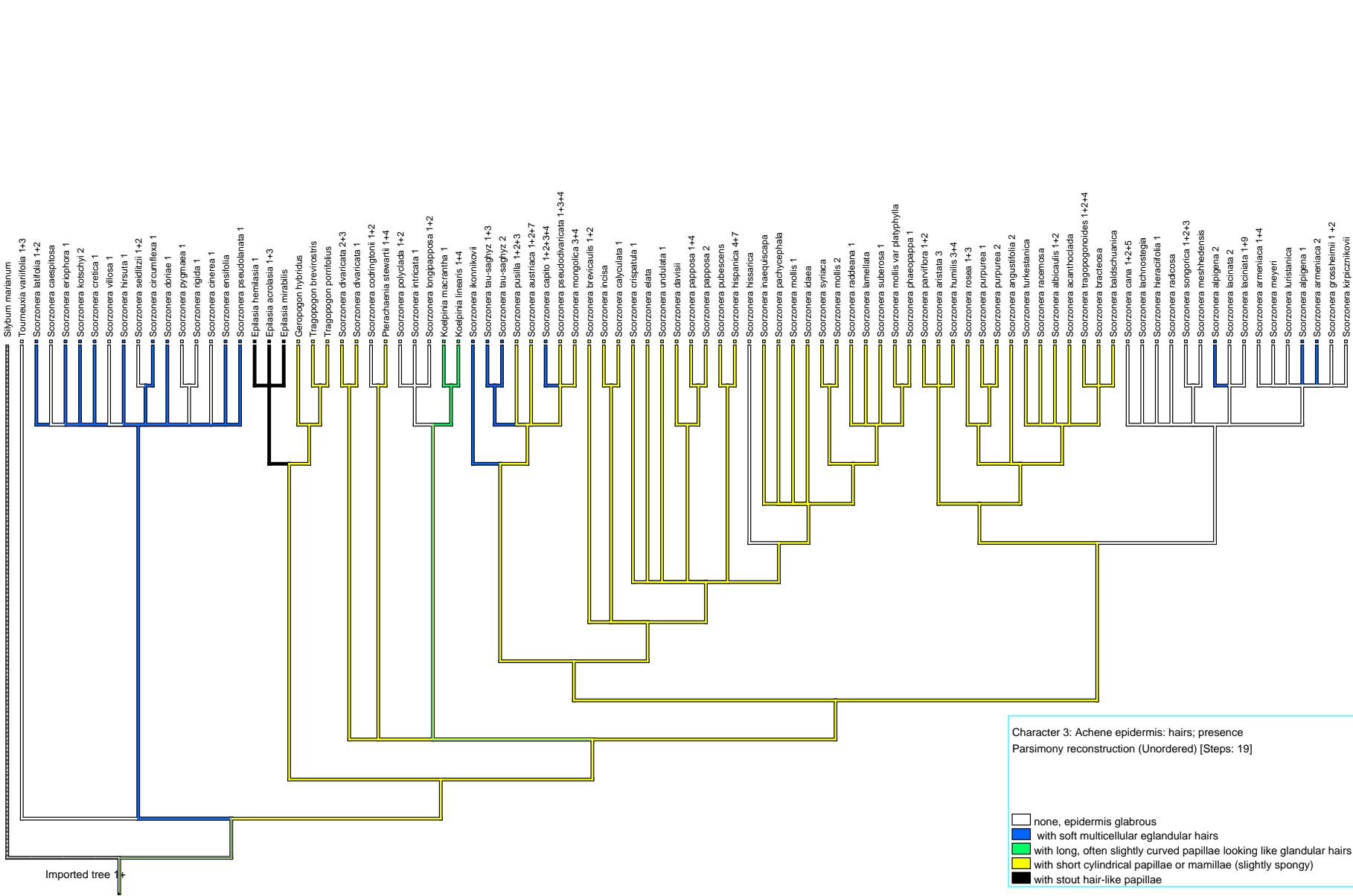

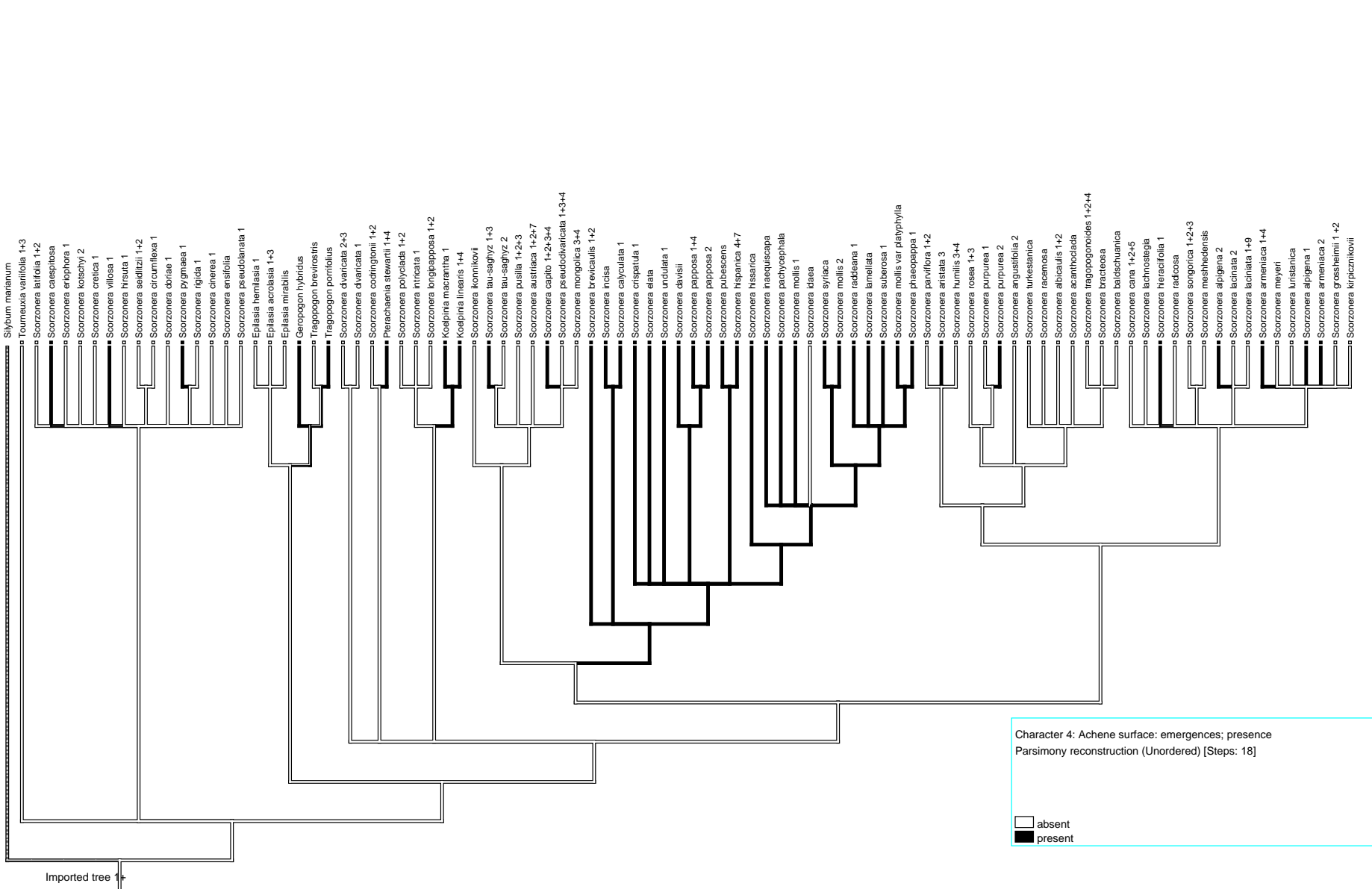

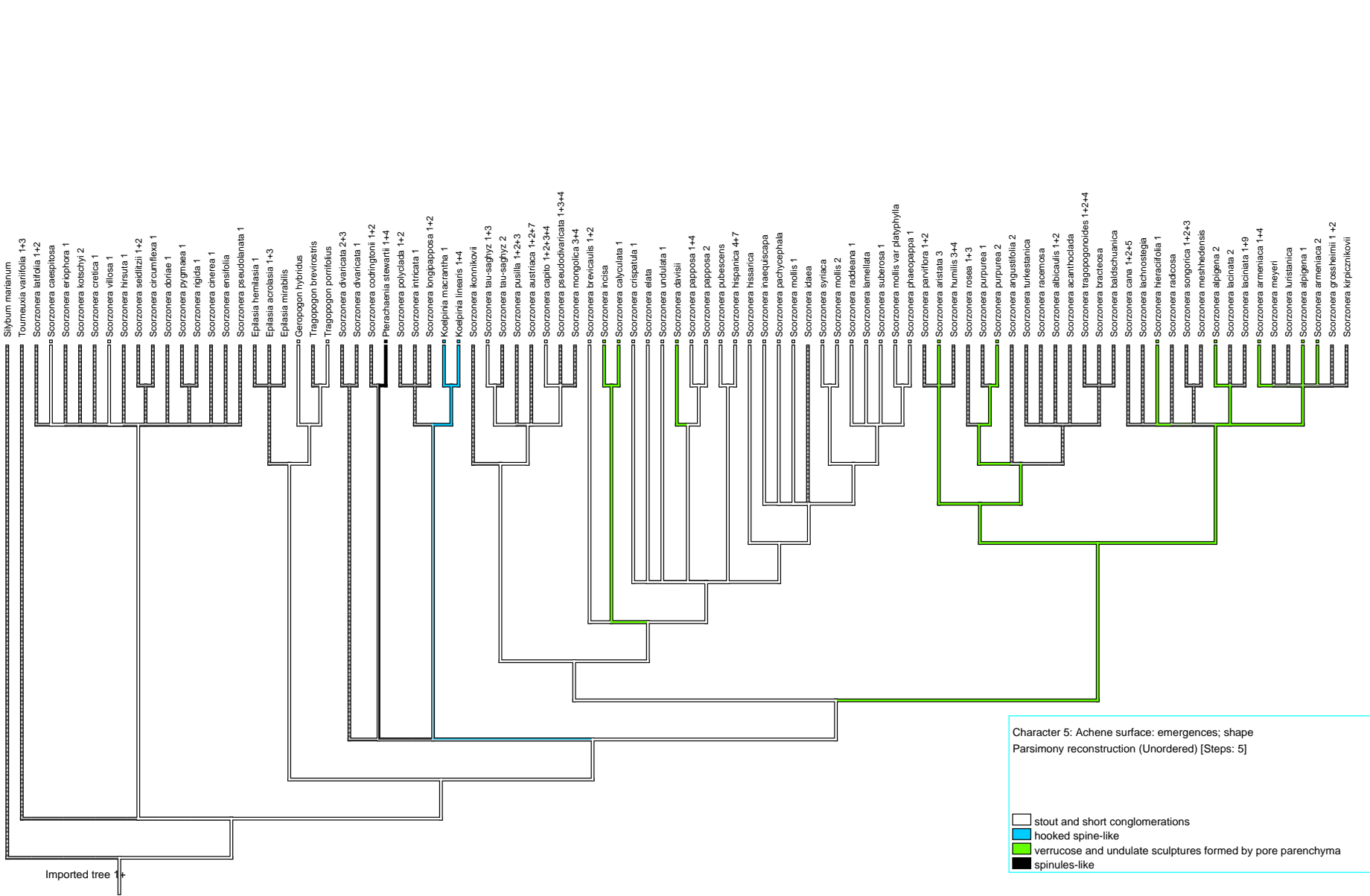

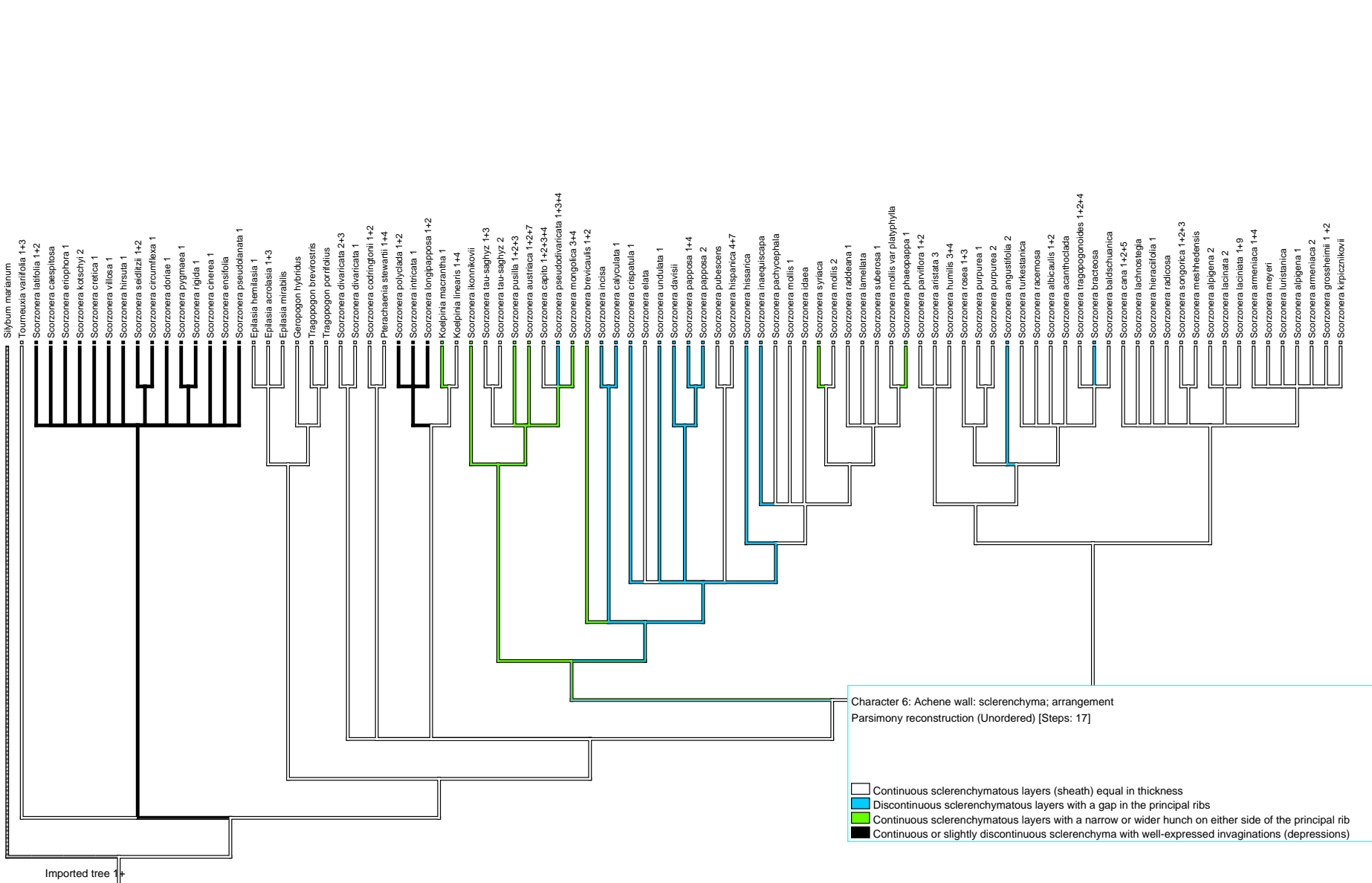

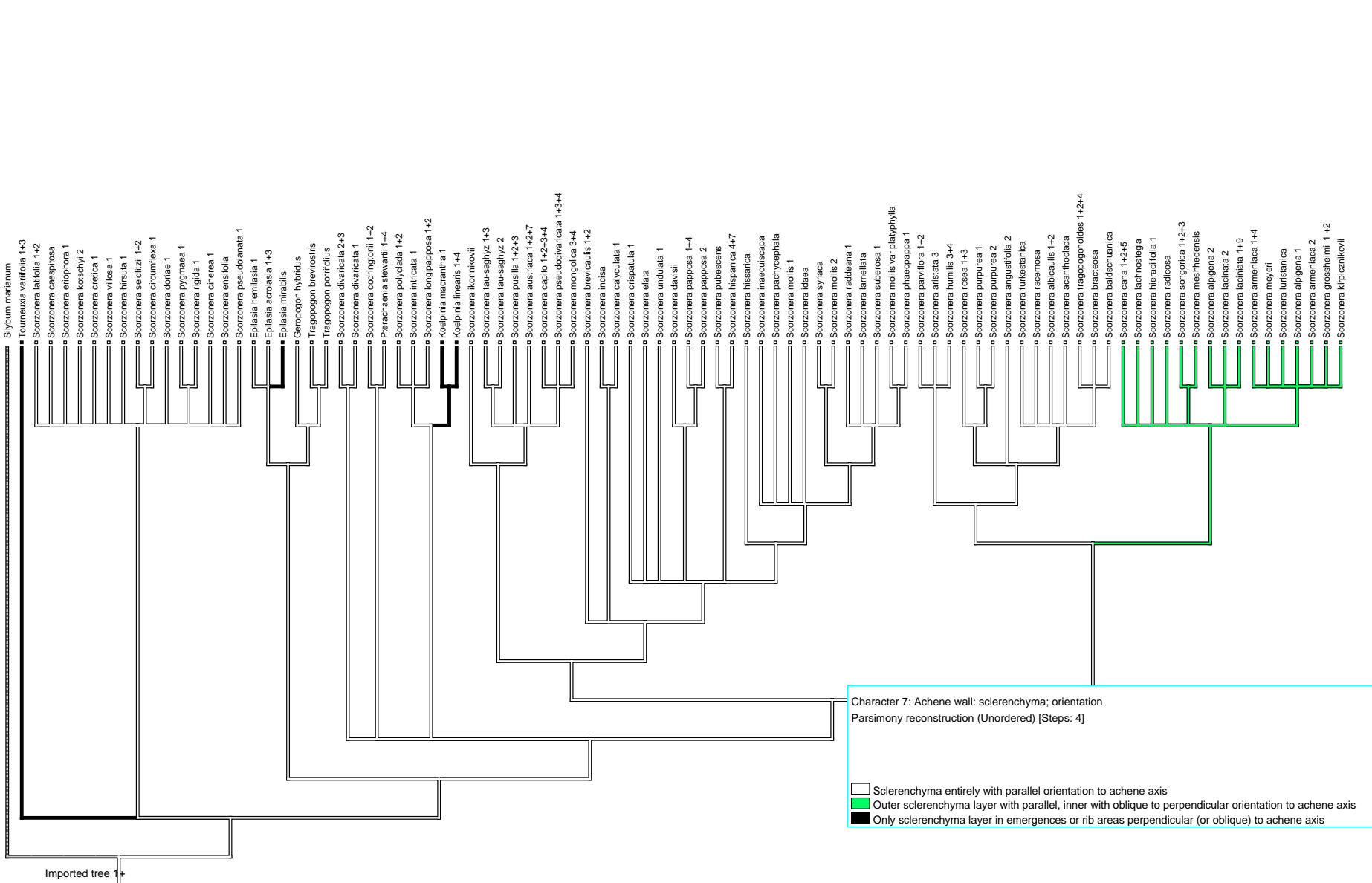

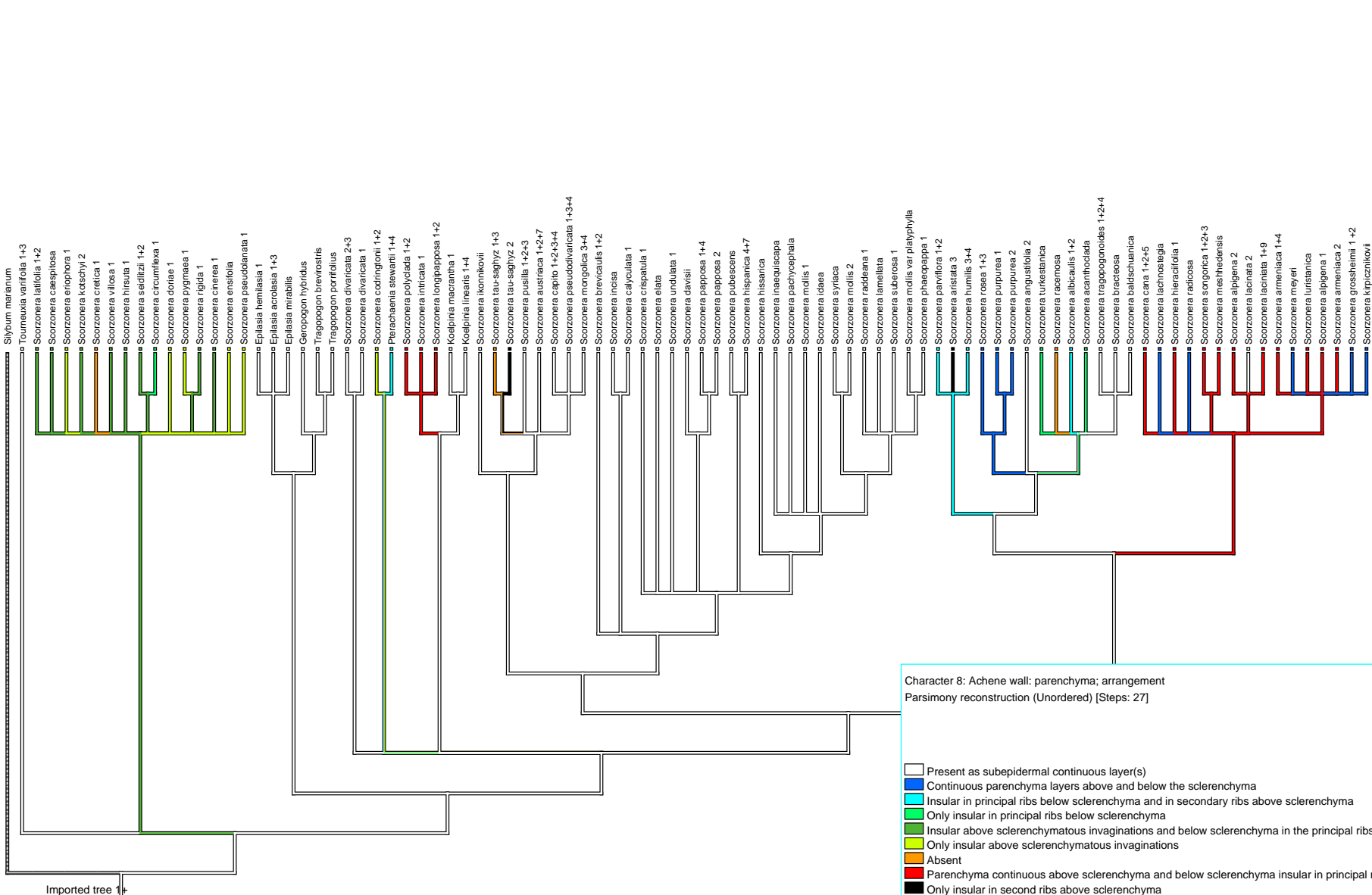

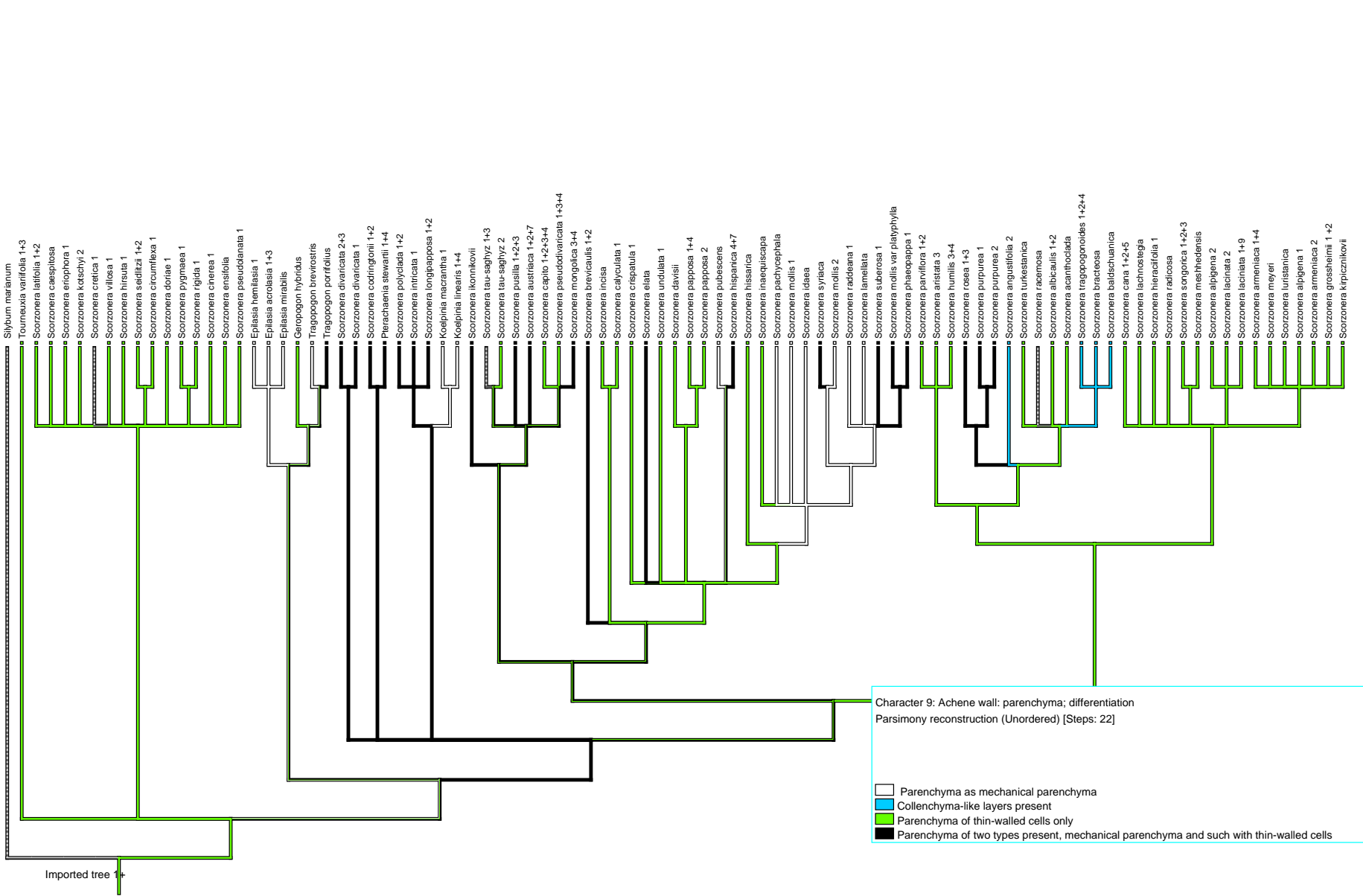

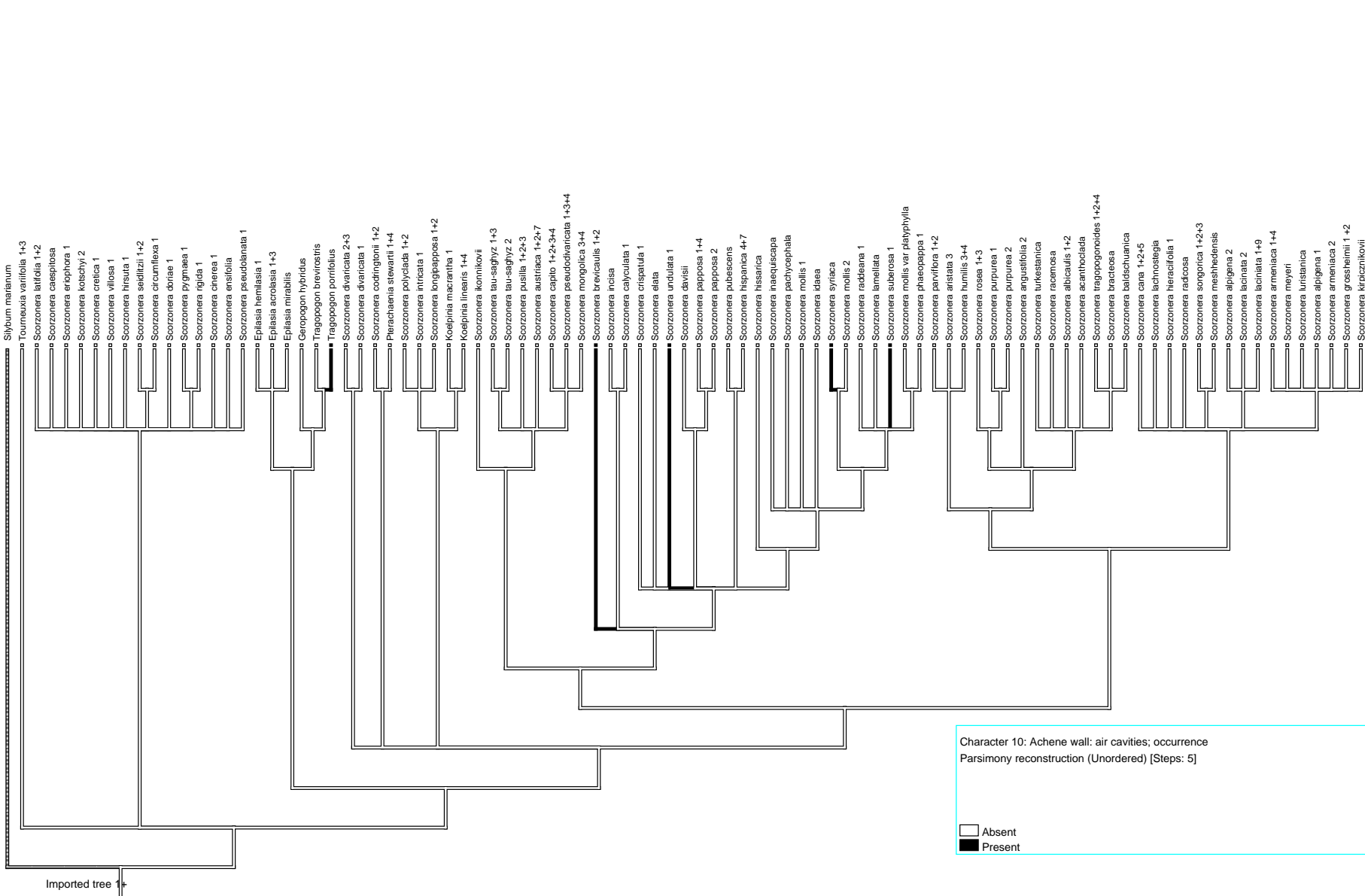

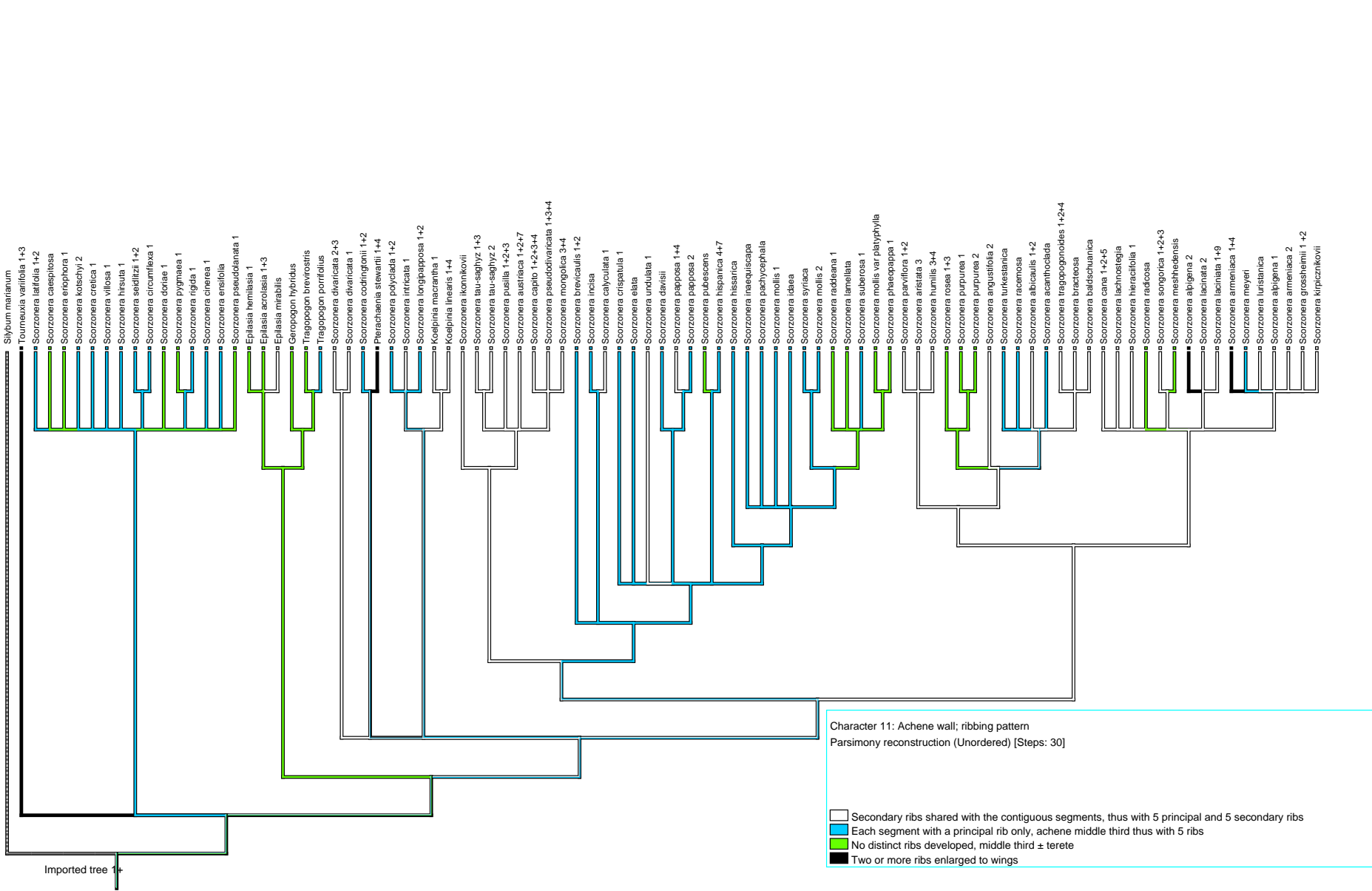

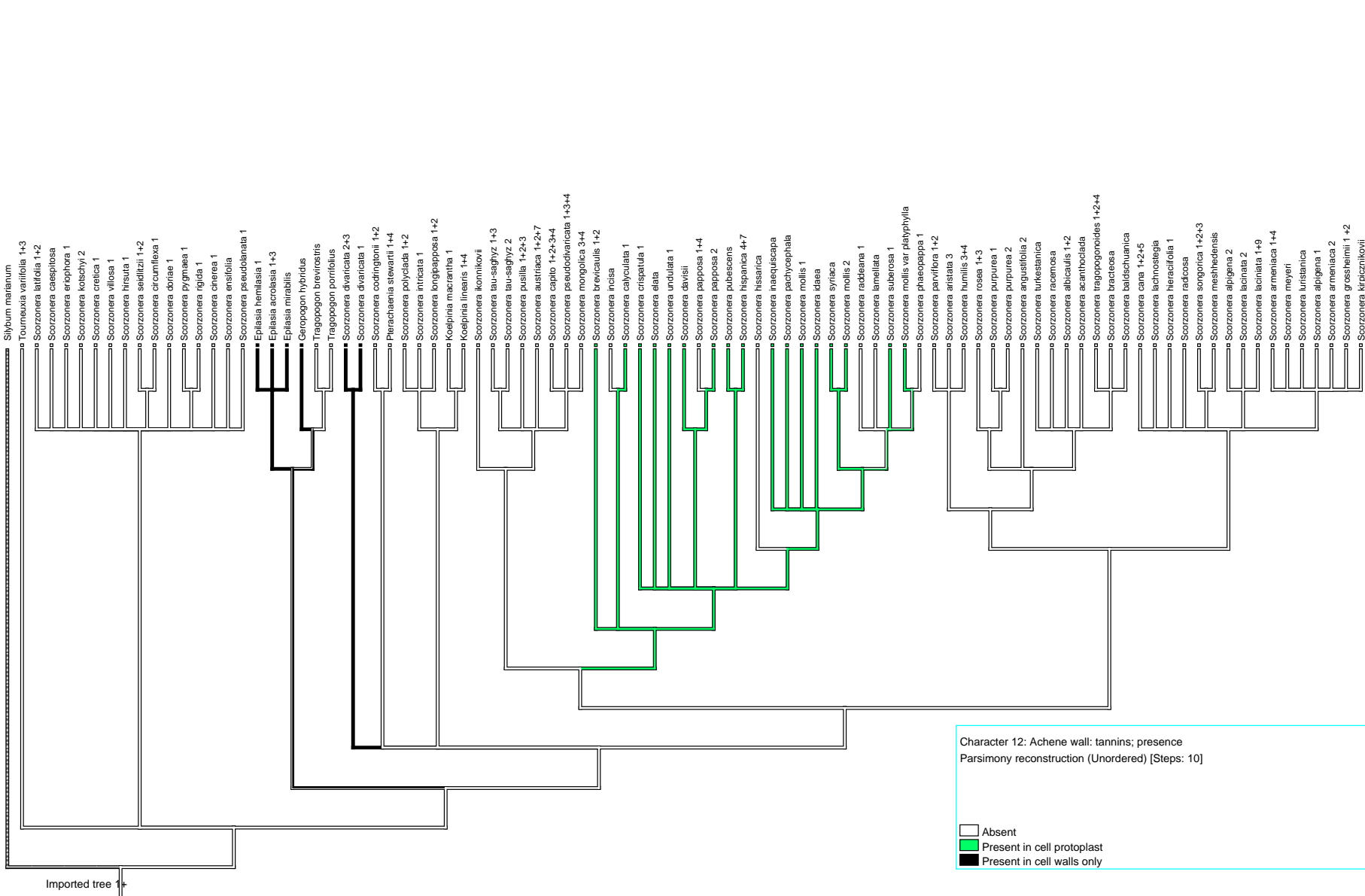

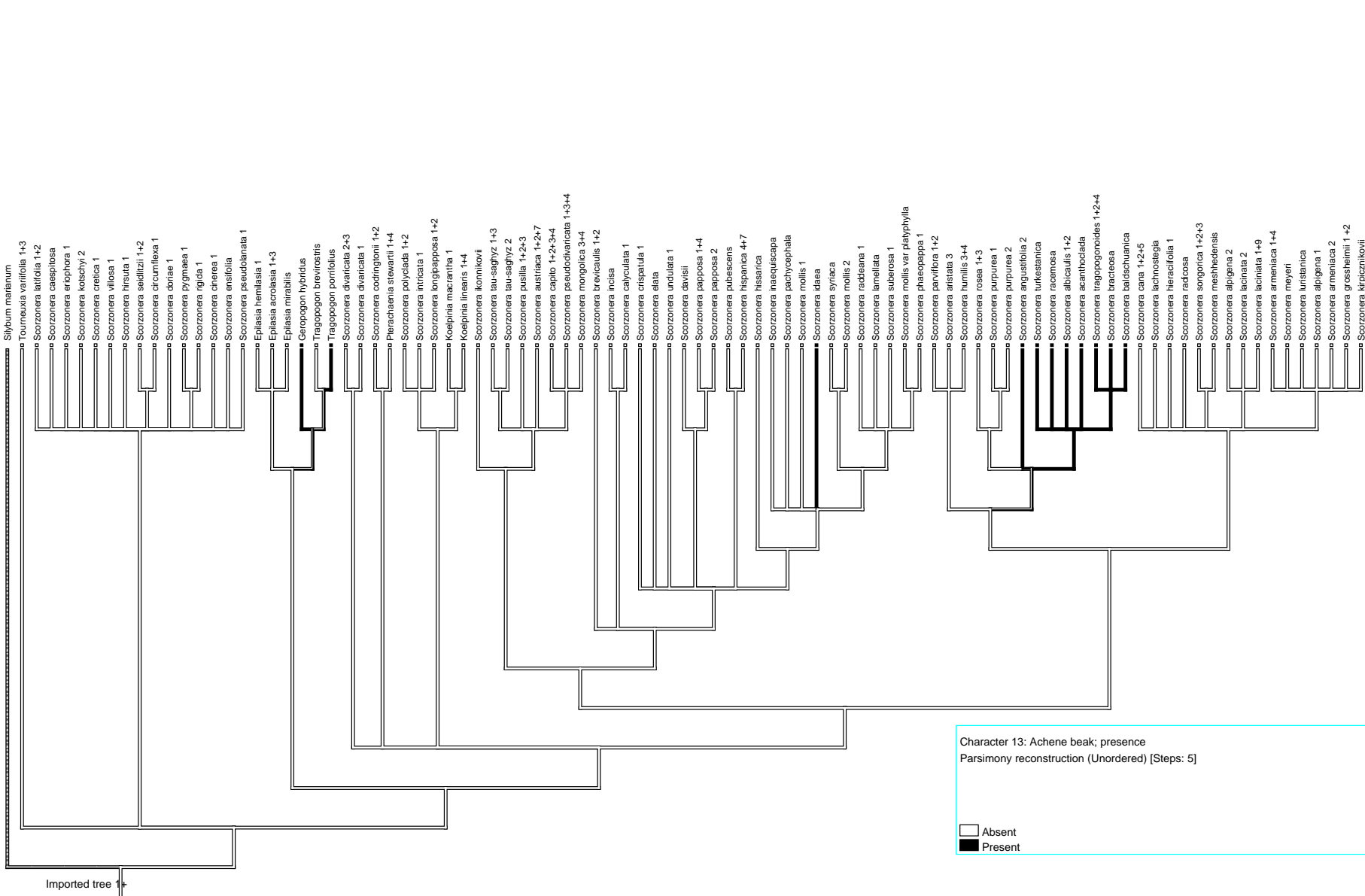

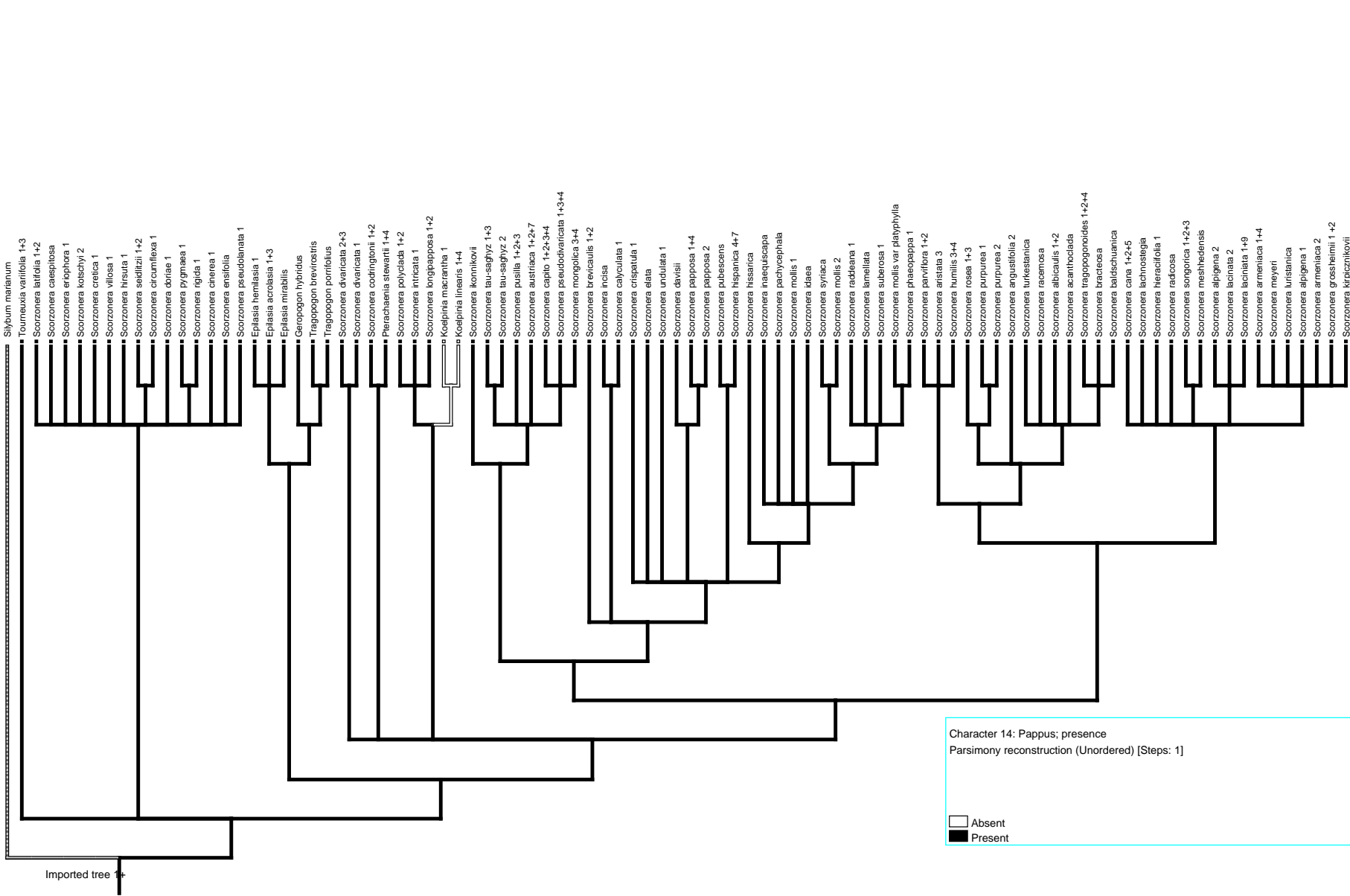

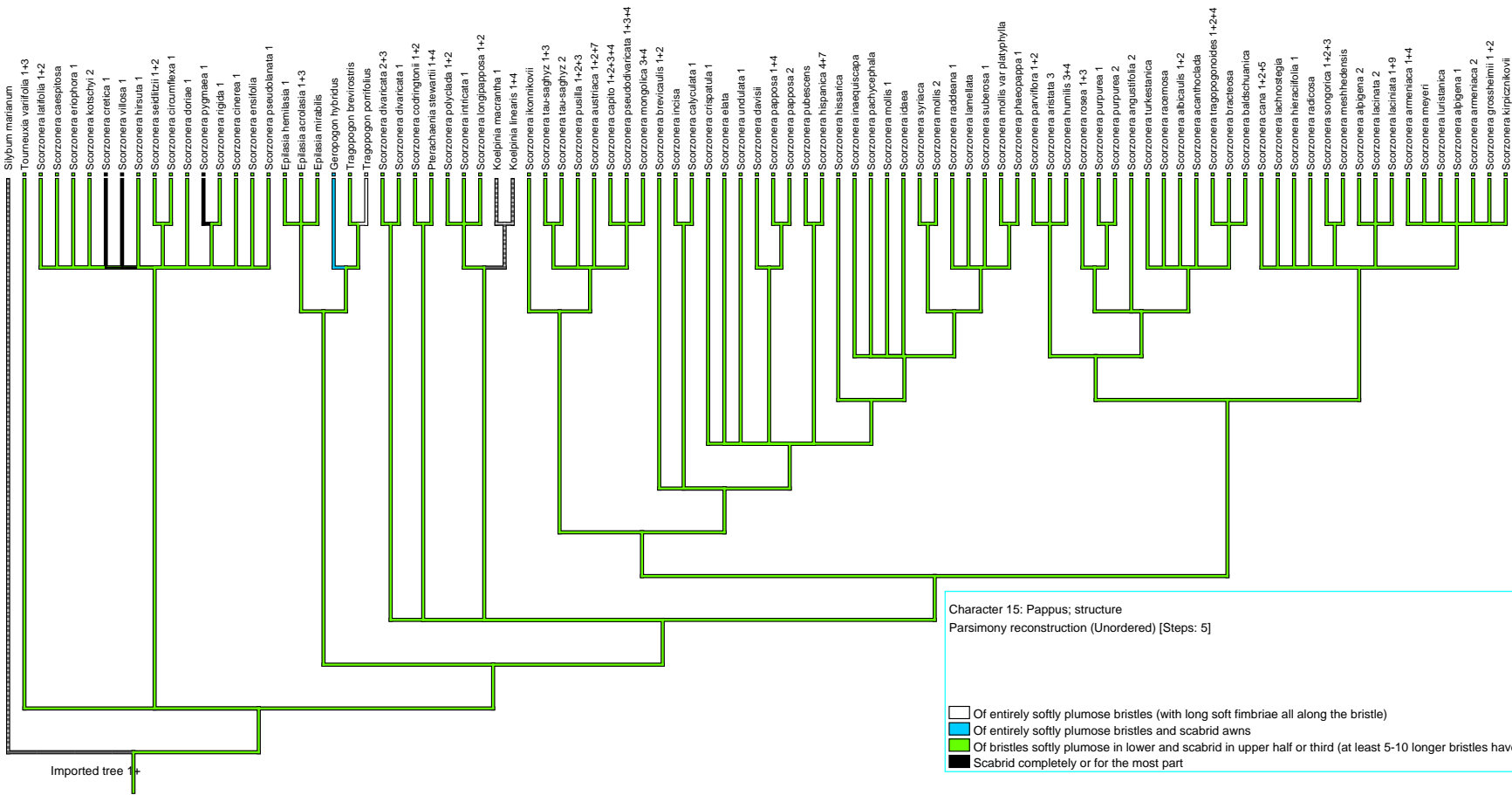

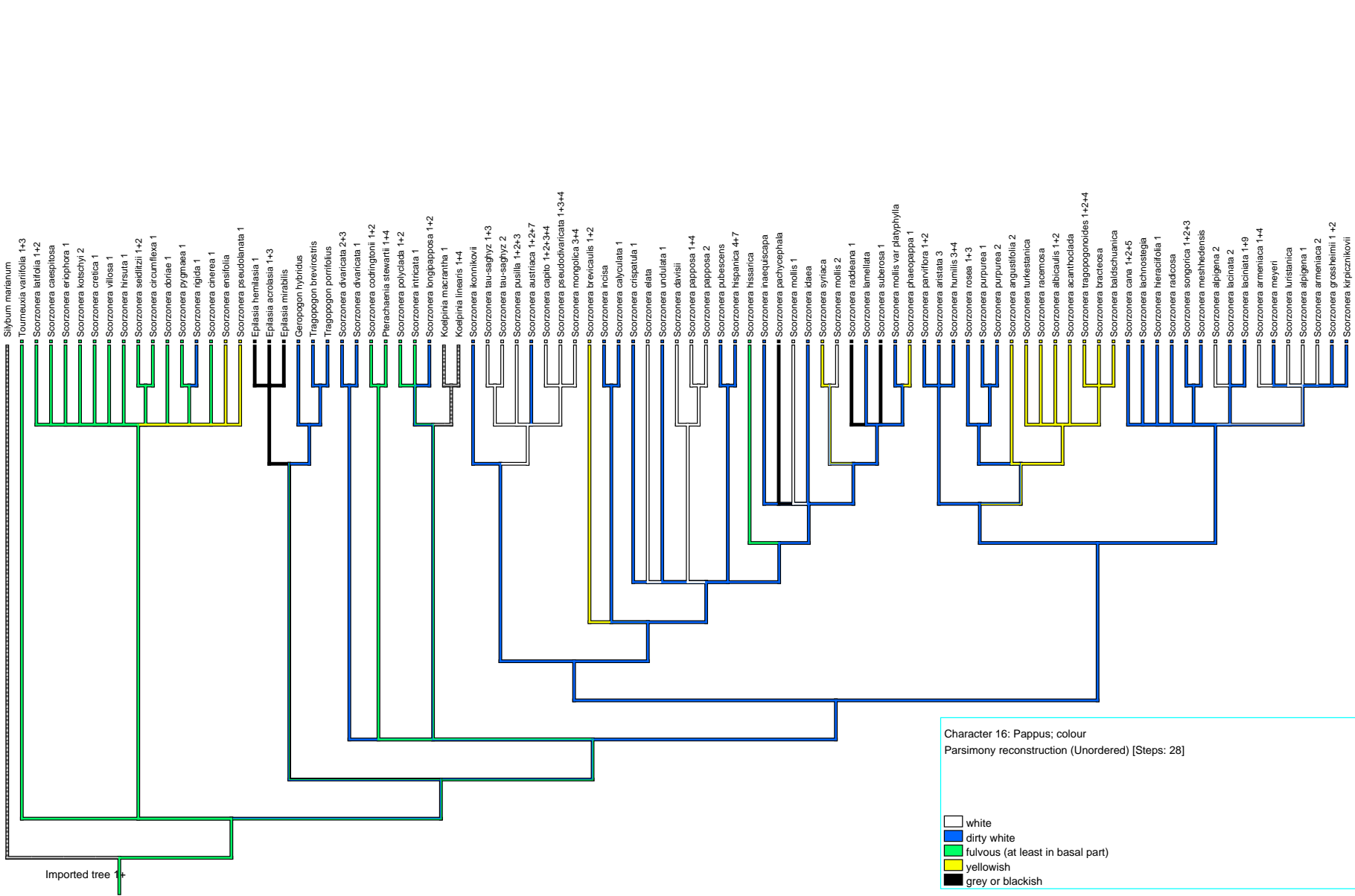

Supplement: Supplementary material 1 [file phytokeys-137-001-s001.pdf]
